# Prediction of Breast Cancer Risk Based on Profiling With Common Genetic Variants

**DOI:** 10.1093/jnci/djv036

**Published:** 2015-04-02

**Authors:** Nasim Mavaddat, Paul D. P. Pharoah, Kyriaki Michailidou, Jonathan Tyrer, Mark N. Brook, Manjeet K. Bolla, Qin Wang, Joe Dennis, Alison M. Dunning, Mitul Shah, Robert Luben, Judith Brown, Stig E. Bojesen, Børge G. Nordestgaard, Sune F. Nielsen, Henrik Flyger, Kamila Czene, Hatef Darabi, Mikael Eriksson, Julian Peto, Isabel dos-Santos-Silva, Frank Dudbridge, Nichola Johnson, Marjanka K. Schmidt, Annegien Broeks, Senno Verhoef, Emiel J. Rutgers, Anthony Swerdlow, Alan Ashworth, Nick Orr, Minouk J. Schoemaker, Jonine Figueroa, Stephen J. Chanock, Louise Brinton, Jolanta Lissowska, Fergus J. Couch, Janet E. Olson, Celine Vachon, Vernon S. Pankratz, Diether Lambrechts, Hans Wildiers, Chantal Van Ongeval, Erik van Limbergen, Vessela Kristensen, Grethe Grenaker Alnæs, Silje Nord, Anne-Lise Borresen-Dale, Heli Nevanlinna, Taru A. Muranen, Kristiina Aittomäki, Carl Blomqvist, Jenny Chang-Claude, Anja Rudolph, Petra Seibold, Dieter Flesch-Janys, Peter A. Fasching, Lothar Haeberle, Arif B. Ekici, Matthias W. Beckmann, Barbara Burwinkel, Frederik Marme, Andreas Schneeweiss, Christof Sohn, Amy Trentham-Dietz, Polly Newcomb, Linda Titus, Kathleen M. Egan, David J. Hunter, Sara Lindstrom, Rulla M. Tamimi, Peter Kraft, Nazneen Rahman, Clare Turnbull, Anthony Renwick, Sheila Seal, Jingmei Li, Jianjun Liu, Keith Humphreys, Javier Benitez, M. Pilar Zamora, Jose Ignacio Arias Perez, Primitiva Menéndez, Anna Jakubowska, Jan Lubinski, Katarzyna Jaworska-Bieniek, Katarzyna Durda, Natalia V. Bogdanova, Natalia N. Antonenkova, Thilo Dörk, Hoda Anton-Culver, Susan L. Neuhausen, Argyrios Ziogas, Leslie Bernstein, Peter Devilee, Robert A. E. M. Tollenaar, Caroline Seynaeve, Christi J. van Asperen, Angela Cox, Simon S. Cross, Malcolm W. R. Reed, Elza Khusnutdinova, Marina Bermisheva, Darya Prokofyeva, Zalina Takhirova, Alfons Meindl, Rita K. Schmutzler, Christian Sutter, Rongxi Yang, Peter Schürmann, Michael Bremer, Hans Christiansen, Tjoung-Won Park-Simon, Peter Hillemanns, Pascal Guénel, Thérèse Truong, Florence Menegaux, Marie Sanchez, Paolo Radice, Paolo Peterlongo, Siranoush Manoukian, Valeria Pensotti, John L. Hopper, Helen Tsimiklis, Carmel Apicella, Melissa C. Southey, Hiltrud Brauch, Thomas Brüning, Yon-Dschun Ko, Alice J. Sigurdson, Michele M. Doody, Ute Hamann, Diana Torres, Hans-Ulrich Ulmer, Asta Försti, Elinor J. Sawyer, Ian Tomlinson, Michael J. Kerin, Nicola Miller, Irene L. Andrulis, Julia A. Knight, Gord Glendon, Anna Marie Mulligan, Georgia Chenevix-Trench, Rosemary Balleine, Graham G. Giles, Roger L. Milne, Catriona McLean, Annika Lindblom, Sara Margolin, Christopher A. Haiman, Brian E. Henderson, Fredrick Schumacher, Loic Le Marchand, Ursula Eilber, Shan Wang-Gohrke, Maartje J. Hooning, Antoinette Hollestelle, Ans M. W. van den Ouweland, Linetta B. Koppert, Jane Carpenter, Christine Clarke, Rodney Scott, Arto Mannermaa, Vesa Kataja, Veli-Matti Kosma, Jaana M. Hartikainen, Hermann Brenner, Volker Arndt, Christa Stegmaier, Aida Karina Dieffenbach, Robert Winqvist, Katri Pylkäs, Arja Jukkola-Vuorinen, Mervi Grip, Kenneth Offit, Joseph Vijai, Mark Robson, Rohini Rau-Murthy, Miriam Dwek, Ruth Swann, Katherine Annie Perkins, Mark S. Goldberg, France Labrèche, Martine Dumont, Diana M. Eccles, William J. Tapper, Sajjad Rafiq, Esther M. John, Alice S. Whittemore, Susan Slager, Drakoulis Yannoukakos, Amanda E. Toland, Song Yao, Wei Zheng, Sandra L. Halverson, Anna González-Neira, Guillermo Pita, M. Rosario Alonso, Nuria Álvarez, Daniel Herrero, Daniel C. Tessier, Daniel Vincent, Francois Bacot, Craig Luccarini, Caroline Baynes, Shahana Ahmed, Mel Maranian, Catherine S. Healey, Jacques Simard, Per Hall, Douglas F. Easton, Montserrat Garcia-Closas

**Affiliations:** **Affiliations of authors:** Centre for Cancer Genetic Epidemiology, Department of Public Health and Primary Care, University of Cambridge, Cambridge, UK (NM, PDPP, KM, MKB, QW, JD, RL, JBr, DFE); Centre for Cancer Genetic Epidemiology, Department of Oncology, University of Cambridge, Cambridge, UK (PDPP, JT, AMD, MS, CL, CB, SA, MM, CSH, DFE); Division of Genetics and Epidemiology, The Institute of Cancer Research, London, UK (MNB, ASw, MJS); Copenhagen General Population Study, Herlev Hospital, Copenhagen University Hospital, Copenhagen, Denmark (SEB, BGN, SFN); Department of Clinical Biochemistry, Herlev Hospital, Copenhagen University Hospital, Copenhagen, Herlev, Denmark (SEB, BGN, SFN); Faculty of Health and Medical Sciences, Copenhagen University Hospital, Copenhagen, Herlev, Denmark (SEB, BGN); Department of Breast Surgery, Herlev Hospital, Copenhagen University Hospital, Copenhagen, Herlev, Denmark (HF); Department of Medical Epidemiology and Biostatistics, Karolinska Institutet, Stockholm, Sweden (KC, HD, ME, KH, PHa); Department of Non-communicable Disease Epidemiology, London School of Hygiene and Tropical Medicine, London, UK (JP, IdSS, FD); Breakthrough Breast Cancer Research Centre, The Institute of Cancer Research, London, UK (NJ, AA, NO, MGC); Netherlands Cancer Institute, Antoni van Leeuwenhoek hospital, Amsterdam, the Netherlands (MKS, AB, SV, EJR); Division of Breast Cancer Research, Institute of Cancer Research, London, UK (ASw); Division of Cancer Epidemiology and Genetics, National Cancer Institute, Rockville, MD (JF, SJC, LB, ASi, MD); Department of Cancer Epidemiology and Prevention, M. Sklodowska-Curie Memorial Cancer Center and Institute of Oncology, Warsaw, Poland (JLis); Department of Laboratory Medicine and Pathology, Mayo Clinic, Rochester, MN (FJC); Department of Health Sciences Research, Mayo Clinic, Rochester, MN (JEO, CV, VSP, SS); Vesalius Research Center, VIB, Leuven, Belgium (DL); Laboratory for Translational Genetics, Department of Oncology, University of Leuven, Leuven, Belgium (DL); Department of General Medical Oncology, University Hospitals Leuven, and Department of Oncology, KU Leuven, Leuven, Belgium (HW); Department of Radiation Oncology, University Hospital Gasthuisberg, Leuven, Belgium (EVL); Department of Radiology, University Hospital Gasthuisberg, Leuven, Belgium (CVO); Department of Genetics, Institute for Cancer Research, Oslo University Hospital, Radiumhospitalet, Oslo, Norway (VK, GGA, SN, ALBD); Institute of Clinical Medicine, University of Oslo, Oslo, Norway (VK, ALBD); Department of Clinical Molecular Biology, University of Oslo, Oslo, Norway (VK); Department of Obstetrics and Gynecology, University of Helsinki and Helsinki University Central Hospital, Helsinki, HUS, Finland (HN, TAM); Department of Clinical Genetics, University of Helsinki and Helsinki University Central Hospital, Helsinki, HUS, Finland (KA); Department of Oncology, University of Helsinki and Helsinki University Central Hospital, Helsinki, HUS, Finland (CB); Division of Cancer Epidemiology, German Cancer Research Center, Heidelberg, Germany (JCC, AR, PS, UE); Department of Cancer Epidemiology/Clinical Cancer Registry, University Clinic Hamburg-Eppendorf, Hamburg, Germany (DFJ); University Breast Center Franconia, Department of Gynecology and Obstetrics, University Hospital Erlangen, Friedrich-Alexander University Erlangen-Nuremberg, Comprehensive Cancer Center Erlangen-EMN, Erlangen, Germany (PAF, LH, MWB); David Geffen School of Medicine, Department of Medicine Division of Hematology and Oncology, University of California at Los Angeles, CA (PAF); Institute of Human Genetics, University Hospital Erlangen, Friedrich Alexander University Erlangen-Nuremberg, Erlangen, Germany (ABE); Department of Obstetrics and Gynecology, University of Heidelberg, Heidelberg, Germany (BB, FM, ASc, CSohn, RY); Molecular Epidemiology Group, German Cancer Research Center, Heidelberg, Germany (BB, RY); National Center for Tumor Diseases, University of Heidelberg, Heidelberg, Germany (FM, ASc); University of Wisconsin Carbone Cancer Center, Madison, WI (ATD, PN); Cancer Prevention Program, Fred Hutchinson Cancer Research Center, Seattle, WA (PN); Geisel School of Medicine at Dartmouth, Hanover, NH (LT); Division of Population Sciences, Moffitt Cancer Center & Research Institute, Tampa, FL (KE); Program in Genetic Epidemiology and Statistical Genetics, Harvard T.H. Chan School of Public Health, Boston, MA (DJH, SL, PK); Department of Epidemiology, Harvard T.H. Chan School of Public Health, Boston, MA (DJH, SL, RMT, PK); Channing Division of Network Medicine, Department of Medicine, Brigham and Women’s Hospital and Harvard Medical School, Boston, MA (RMT); Section of Cancer Genetics, Institute of Cancer Research, London, UK (NR, CT, AR, SS); Human Genetics Division, Genome Institute of Singapore, Singapore (JLi, JLiu); Human Genotyping-CEGEN Unit, Human Cancer Genetics Programme, Spanish National Cancer Research Centre, Madrid, Spain (JBe, AGN, GP, MRA, NA, DH); Centro de Investigación en Red de Enfermedades Raras, Valencia, Spain (JBe); Servicio de Oncología Médica, Hospital Universitario La Paz, Madrid, Spain (MPZ); Servicio de Cirugía General y Especialidades, Hospital Monte Naranco, Oviedo, Spain (JIAP); Servicio de Anatomía Patológica, Hospital Monte Naranco, Oviedo, Spain (PM); Department of Genetics and Pathology, Pomeranian Medical University, Szczecin, Poland (AJ, JLu, KJB, KD); Department of Obstetrics and Gynaecology, Hannover Medical School, Hannover, Germany (NVB, TD, PS, TWPS, PHi); Department of Radiation Oncology, Hannover Medical School, Hannover, Germany (NVB, MB, HC); NN Alexandrov Research Institute of Oncology and Medical Radiology, Minsk, Belarus (NNA); Department of Epidemiology, University of California Irvine, Irvine, CA (HAC, AZ); Department of Population Sciences, Beckman Research Institute of City of Hope, Duarte, CA (SLN, LB); Department of Human Genetics and Department of Pathology, Leiden University Medical Center, Leiden, the Netherlands (PD); Department of Surgical Oncology, Leiden University Medical Center, Leiden, the Netherlands (RAEMT); Department of Clinical Genetics, Leiden University Medical Center, Leiden, the Netherlands (CJvA); Sheffield Cancer Research, Department of Oncology, University of Sheffield, UK (AC, MWRR); Academic Unit of Pathology, Department of Neuroscience, University of Sheffield, Sheffield, UK (SSC); Institute of Biochemistry and Genetics, Ufa Scientific Center of Russian Academy of Sciences, Ufa, Russia (EK, MB, ZT); Department of Genetics and Fundamental Medicine of Bashkir State University, Ufa, Russia (EK, DP); Division of Gynaecology and Obstetrics, Technische Universität München, Munich, Germany (AMe); Center for Hereditary Breast and Ovarian Cancer, University Hospital Cologne, Cologne, Germany (RKS); Center for Integrated Oncology, University Hospital Cologne, Cologne, Germany (RKS); Center for Molecular Medicine Cologne, University of Cologne, Cologne, Germany (RKS); Institute of Human Genetics, University Heidelberg, Heidelberg, Germany (CSutter); National Institute of Health and Medical Research, Center for Research in Epidemiology and Population Health, U1018, Environmental Epidemiology of Cancer, Villejuif, France (PG, TT, FM, MS); University Paris-Sud, Villejuif, France (PG, TT, FM, MS); Unit of Molecular Bases of Genetic Risk and Genetic Testing, Department of Preventive and Predictive Medicine, Fondazione IRCCS Istituto Nazionale Tumori, Milan, Italy (PR); IFOM, Fondazione Istituto FIRC di Oncologia Molecolare, Milan, Italy (PP, VP); Unit of Medical Genetics, Department of Preventive and Predictive Medicine, Fondazione IRCCS Istituto Nazionale Tumori, Milan, Italy (SM); Cogentech Cancer Genetic Test Laboratory, Milan, Italy (VP); Centre for Epidemiology & Biostatistics, Melbourne School of Population and Global Health, University of Melbourne, Melbourne, Victoria, Australia (JLH, CA, GGG, RLM); Department of Pathology, University of Melbourne, Melbourne, Victoria, Australia (HT, MCS); Dr. Margarete Fischer-Bosch-Institute of Clinical Pharmacology, Stuttgart, Germany, for the GENICA Network (HB); University of Tübingen, Tübingen, Germany, for the GENICA Network (HB); German Cancer Consortium, German Cancer Research Center (DKFZ), Heidelberg, Germany (HBra, HBre, AKD); Institute for Prevention and Occupational Medicine of the German Social Accident Insurance, Institute of the Ruhr-Universität Bochum (IPA), Germany, for the GENICA Network (TB); Department of Internal Medicine, Evangelische Kliniken Bonn gGmbH, Johanniter Krankenhaus, Bonn, Germany, for the GENICA Network (YDK); Molecular Genetics of Breast Cancer, German Cancer Research Center, Heidelberg, Germany, for the GENICA Network (UH); Institute of Human Genetics, Pontificia Universidad Javeriana, Bogota, Colombia (DT); Frauenklinik der Stadtklinik Baden-Baden, Baden-Baden, Germany (HUU); Division of Molecular Genetic Epidemiology, German Cancer Research Center, Heidelberg, Germany (AF); Center for Primary Health Care Research, University of Lund, Malmö, Sweden (AF); Division of Cancer Studies, Kings College London, Guy’s Hospital, London, UK (EJS); Wellcome Trust Centre for Human Genetics and Oxford Biomedical Research Centre, University of Oxford, Oxford, UK (IT); Clinical Science Institute, University Hospital Galway, Galway, Ireland (MJK, NM); Ontario Cancer Genetics Network, Lunenfeld-Tanenbaum Research Institute of Mount Sinai Hospital, Toronto, Ontario, Canada (ILA, GG); Department of Molecular Genetics, University of Toronto, Toronto, Ontario, Canada (ILA); Prosserman Centre for Health Research, Lunenfeld-Tanenbaum Research Institute, Mount Sinai Hospital, Toronto, Ontario, Canada (JAK); Division of Epidemiology, Dalla Lana School of Public Health, University of Toronto, Toronto, Ontario, Canada (JAK); Department of Laboratory Medicine and Pathobiology, University of Toronto, Toronto, Ontario, Canada (AMM); Laboratory Medicine Program, University Health Network, Toronto, Ontario, Canada (AMM); Department of Genetics, QIMR Berghofer Medical Research Institute, Brisbane, Australia, for the Australian Ovarian Cancer Study Group (GCT); Peter MacCallum Cancer Center, Melbourne, Victoria, Australia, for kConFab Investigators and the Australian Ovarian Cancer Study Group; Westmead Millenium Institute for Medical Research, University of Sydney, Sydney, NSW, Australia (RB, CC); Western Sydney and Nepean Blue Mountains Local Health Districts, Sydney, Australia (RB); Cancer Epidemiology Centre, Cancer Council Victoria, Melbourne, Victoria, Australia (GGG, RLM); Anatomical Pathology, The Alfred Hospital, Melbourne, Victoria, Australia (CM); Department of Molecular Medicine and Surgery, Karolinska Institutet, Stockholm, Sweden (AL); Department of Oncology - Pathology, Karolinska Institutet, Stockholm, Sweden (SM); Department of Preventive Medicine, Keck School of Medicine, University of Southern California, Los Angeles, CA (CAH, BEH, FS); Epidemiology Program, University of Hawaii Cancer Center, Honolulu, HI (LLM); Department of Obstetrics and Gynecology, University of Ulm, Ulm, Germany (SWG); Department of Medical Oncology, Family Cancer Clinic, Erasmus MC Cancer Institute, Rotterdam, the Netherlands (MJH, AH, CS); Department of Clinical Genetics, Erasmus University Medical Center, Rotterdam, the Netherlands (AMWvdO); Department of Surgical Oncology, Family Cancer Clinic, Erasmus MC Cancer Institute, Rotterdam, the Netherlands (LBK); Australian Breast Cancer Tissue Bank, Westmead Millennium Institute, University of Sydney, Sydney, NSW, Australia, for the ABCTB Investigators (JC); Division of Genetics, Hunter Area Pathology Service and University of Newcastle, Callaghan, NSW, Australia (RSc); School of Medicine, Institute of Clinical Medicine, Pathology and Forensic Medicine (AMa, VMK, JMH); Cancer Center of Eastern Finland, University of Eastern Finland, Kuopio, Finland (AMa, VMK, JMH); Imaging Center, Department of Clinical Pathology, Kuopio University Hospital, Kuopio, Finland (AMa, VMK, JMH); Cancer Center, Kuopio University Hospital, Kuopio, Finland, and Jyvaskyla Central Hospital, Jyvaskyla, Finland (VK); Division of Clinical Epidemiology and Aging Research, German Cancer Research Center, Heidelberg, Germany (HB, VA, AKD); Saarland Cancer Registry, Saarbrücken, Germany (CStegmaier); Laboratory of Cancer Genetics and Tumor Biology, Department of Clinical Chemistry and Biocenter Oulu, University of Oulu, Northern Finland Laboratory Centre NordLab, Oulu, Finland (RW, KP); Department of Oncology, Oulu University Hospital, University of Oulu, Oulu, Finland (AJV); Department of Surgery, Oulu University Hospital, University of Oulu, Oulu, Finland (MG); Clinical Genetics Service, Department of Medicine, Memorial Sloan-Kettering Cancer Center, New York, NY (KO, JV, MR, RRM); Clinical Genetics Research Lab, Department of Cancer Biology and Genetics, Memorial Sloan-Kettering Cancer Center, New York, NY (KO, JV); Department of Molecular and Applied Biosciences, Faculty of Science and Technology, University of Westminster, London, UK (MDw, RSw, KAP); Department of Medicine, McGill University, Montreal, Quebec, Canada (MSG); Division of Clinical Epidemiology, McGill University Health Centre, Royal Victoria Hospital, Montreal, Quebec, Canada (MSG); Département de médecine sociale et préventive, Département de santé environnementale et santé au travail, Université de Montréal, Montreal, Quebec, Canada (FL); Cancer Genomics Laboratory for Genomics Centre, Centre Hospitalier Universitaire de Québec Research Centre and Laval University, Québec City, Quebec, Canada (MDu, JS); Faculty of Medicine, University of Southampton, UK (DME, WJT, SR); Cancer Prevention Institute of California, Fremont, CA (EMJ); Department of Health Research and Policy Stanford University School of Medicine Stanford CA (EMJ, ASW); Molecular Diagnostics Laboratory, IRRP, National Centre for Scientific Research “Demokritos”, Aghia Paraskevi Attikis, Athens, Greece (DY); Department of Molecular Virology, Immunology and Medical Genetics, Comprehensive Cancer Center, The Ohio State University, Columbus, OH (AET); Department of Cancer Prevention and Control, Roswell Park Cancer Institute, Buffalo, NY (SY); Division of Epidemiology, Department of Medicine, Vanderbilt Epidemiology Center, Vanderbilt-Ingram Cancer Center, Vanderbilt University School of Medicine, Nashville, TN (WZ, SLH); McGill University and Génome Québec Innovation Centre, Montréal, Québec, Canada (DCT, DV, FB).

## Abstract

**Background:**

Data for multiple common susceptibility alleles for breast cancer may be combined to identify women at different levels of breast cancer risk. Such stratification could guide preventive and screening strategies. However, empirical evidence for genetic risk stratification is lacking.

**Methods:**

We investigated the value of using 77 breast cancer-associated single nucleotide polymorphisms (SNPs) for risk stratification, in a study of 33 673 breast cancer cases and 33 381 control women of European origin. We tested all possible pair-wise multiplicative interactions and constructed a 77-SNP polygenic risk score (PRS) for breast cancer overall and by estrogen receptor (ER) status. Absolute risks of breast cancer by PRS were derived from relative risk estimates and UK incidence and mortality rates.

**Results:**

There was no strong evidence for departure from a multiplicative model for any SNP pair. Women in the highest 1% of the PRS had a three-fold increased risk of developing breast cancer compared with women in the middle quintile (odds ratio [OR] = 3.36, 95% confidence interval [CI] = 2.95 to 3.83). The ORs for ER-positive and ER-negative disease were 3.73 (95% CI = 3.24 to 4.30) and 2.80 (95% CI = 2.26 to 3.46), respectively. Lifetime risk of breast cancer for women in the lowest and highest quintiles of the PRS were 5.2% and 16.6% for a woman without family history, and 8.6% and 24.4% for a woman with a first-degree family history of breast cancer.

**Conclusions:**

The PRS stratifies breast cancer risk in women both with and without a family history of breast cancer. The observed level of risk discrimination could inform targeted screening and prevention strategies. Further discrimination may be achievable through combining the PRS with lifestyle/environmental factors, although these were not considered in this report.

Breast cancer is the most common cancer among Western women, with approximately 1.67 million cases diagnosed annually worldwide (1). Strategies such as endocrine risk–reducing medication and early detection by breast cancer screening can reduce the burden of disease but have disadvantages including side effects, overdiagnosis, and increased cost (2–4). Stratification of women according to the risk of developing breast cancer could improve risk reduction and screening strategies by targeting those most likely to benefit (5–8).

Both genetic and lifestyle factors are implicated in the aetiology of breast cancer. Women with a history of breast cancer in a first-degree relative are at approximately two-fold higher risk than women without a family history (9). Rare high-risk mutations particularly in the *BRCA1* and *BRCA2* genes explain less than 20% of the two-fold familial relative risk (FRR) (10) and account for a small proportion of breast cancer cases in the general population. Low frequency variants conferring intermediate risk, such as those in *CHEK2*, *ATM*, and *PALB2*, explain 2% to 5% of the FRR. Genome-wide association studies (GWAS) have led to the discovery of multiple common, low-risk variants (single nucleotide polymorphisms [SNPs]) associated with breast cancer risk (11), many of which are differentially associated by estrogen receptor (ER) status (12,13). Recently, new risk-associated variants have been identified in a large-scale replication study conducted by the Breast Cancer Association Consortium (BCAC) as part of the Collaborative Oncological Gene-Environment Study (COGS). SNPs were genotyped in over 40 000 breast cancer cases and 40 000 control women, using a custom array (iCOGS). This experiment increased the number of SNPs robustly associated with breast cancer from 27 to more than 70 and identified additional variants specific to ER-negative breast cancer (14–17).

Risks conferred by SNPs are not sufficiently large to be useful in risk prediction individually. However, the combined effect of multiple SNPs could achieve a degree of risk discrimination that is useful for population-based programmes of breast cancer prevention and early detection (8,18). In this report, we investigated the value of using all 77 breast cancer susceptibility loci identified to date for risk stratification. Previous studies of polygenic risk have assumed a log-additive model for combining SNPs; however, this assumption needs to be evaluated empirically. We first assessed whether interaction between SNP pairs could influence the joint contribution of genetic factors on disease risk by testing for all possible pair-wise interactions between SNPs. We then constructed polygenic risk scores (PRSs) to capture the combined effects of the 77 SNPs on overall breast cancer risk, as well as on the risk of ER-positive and ER-negative disease separately. We estimated absolute risks of developing breast cancer for different levels of the PRS, accounting for the competing risk of mortality from other causes. Effect sizes were confirmed in one large study (pKARMA) that was not part of any SNP discovery set. We discuss the degree of breast cancer risk stratification obtained in women with and without a family history of breast cancer.

## Methods

### Study Subjects and Genotyping

Study participants for the primary analyses (set 1) were 89 049 women of European origin participating in 41 studies in BCAC. All studies were approved by the relevant institutional review boards, and all individuals gave written informed consent. Samples were genotyped using a custom Illumina iSelect array (iCOGS) comprising 211 155 SNPs (15). For some analyses, a further 72 014 women in BCAC genotyped for the relevant SNPs in earlier experiments were included (set 2). For PRS analyses (67 054 women), studies that oversampled breast cancer cases with a family history (21 995 women) were excluded. Supplementary Tables 1–3 (available online) show study designs and numbers of breast cancer cases and control women included.

Analyses were based primarily on variants reported to be associated (at *P* < 5x10^-8^) by COGS or previous publications, with either breast cancer overall or ER-negative disease. SNPs and regions included are summarized in Supplementary Table 4 (available online).

### Statistical Methods

Tests for pair-wise SNP*SNP interactions (departures from a multiplicative model) were carried out using logistic regression, with breast cancer as the outcome. The two SNPs were each coded as a categorical variable (ie, fitting a separate parameter for heterozygous and risk-allele homozygous genotypes), while the interaction term (SNP1*SNP2) was included as continuous covariate. All analyses were adjusted for study and seven principal components (PC) to account for population substructure (15). Additional interaction tests used are described in the Supplementary Methods (available online).

To investigate the association between breast cancer risk and the combined effects of 77 SNPs, a PRS was derived for each individual using the formula:

$$\text{PRS}=\beta_{\text{1}}x_{\text{1}}+\beta_{\text{2}}x_{\text{2}}+\ldots \;\beta_{\text{k}}x_{\text{k}}\ldots +\beta_{n}x_{n}$$

where *β*_k_ is the per-allele log odds ratio (OR) for breast cancer associated with the minor allele for SNP *k*, and *x*_*k*_ the number of alleles for the same SNP (0, 1, or 2), and n = 77 is the total number of SNPs. Thus, the PRS summarizes the combined effect of the SNPs, ignoring departures from a multiplicative model (18). SNPs and corresponding odds ratios used in derivation of PRSs are summarized in Supplementary Table 4 (available online).

Logistic regression models were used to estimate the odds ratios for breast cancer by percentile of the PRS, with the middle quintile category (40^th^ to 60^th^ percentile) as the reference. Observed odds ratios for breast cancer by percentile of the PRS were compared with predicted odds ratios under a multiplicative polygenic model of inheritance. Modification of the PRS by age or by family history of breast cancer in a first-degree relative was evaluated by fitting additional interaction terms in the model. All tests of statistical significance were two-sided. The thresholds for statistical significance are indicated below.

The absolute risk of overall breast cancer, ER-positive and ER-negative breast cancer for individuals in each risk category, was calculated taking into account the competing risk of dying from other causes apart from breast cancer. Approximate confidence limits for the absolute risk were derived from the variance-covariance matrix of the log (relative risk) parameters in the logistic regression analysis. Detailed methods are provided in Supplementary Methods (available online).

## Results

### Pairwise Multiplicative SNP*SNP Interaction Analyses

Data on 46 450 breast cancer cases and 42 599 controls from 41 studies were included in the interaction analyses (Supplementary Table 3, available online). There was no strong evidence for interaction between any particular SNP pair after Bonferroni correction (Supplementary Tables 5–6, available online). Plots of expected vs observed log_10_ *P* values for SNP*SNP interaction tests showed slight departure from the null hypothesis of multiplicative effects (Supplementary Figure 1, A and B, available online), and the number of statistically significant interactions with *P*_interaction_ values of less than .01 was larger than expected by chance (Table 1). To investigate whether there was an excess of synergistic or antagonistic interactions, the direction of the interaction term relative to the main effects was examined for SNP pairs with *P*_interaction_ values of less than .01. For case-control analyses, 47% of interactions were synergistic and 53% antagonistic, and for case-only analyses 53% were synergistic and 46% antagonistic. These proportions were not statistically significantly different from the null expectation (*P* > .05). Meta-analysis of SNP*SNP interaction test results from the iCOGS dataset with those from 72 014 additional women in BCAC yielded similar results (Supplementary Table 7, available online). Given that no SNP pair showed strong evidence for departure from the multiplicative model, subsequent analyses were based on a PRS that included the main effects of SNPs but no SNP*SNP interaction terms.

**Table 1.** Observed and expected numbers of statistically significant pair-wise tests for SNP*SNP interaction at *P* < .01†

Type of breast cancer	Case-control analyses			Case-only analyses‡		
	OBS	OBS/EXP	*P*§	OBS	OBS/EXP	*P*
All SNPs||	n = 3080 SNP pairs			n = 3028 SNP pairs		
All breast cancers	44	1.43	.01	45	1.49	.01
ER-positive	43	1.40	.02	39	1.29	.07
ER-negative	35	1.13	.25	37	1.22	.13
Unlinked SNPs¶	n = 2556 SNP pairs			n = 2522 SNP pairs		
All breast cancers	35	1.37	.04	36	1.43	.02
ER-positive	38	1.49	.01	34	1.35	.05
ER-negative	30	1.17	.21	30	1.19	.19

† 46 450 breast cancer cases and 42 599 control women were included in the analysis of all breast cancers. 27 074 breast cancer cases were included in the analysis of ER-positive disease and 7413 breast cancer cases were included in the analysis of ER-negative disease.

n = number of single nucleotide polymorphsism (SNP) pairs tested; OBS = number of tests observed with *P*_interaction_ < .01; OBS/EXP = number of tests observed with *P*_interaction_ < .01 divided by the number of positive tests expected by chance, given the number of SNP pairs tested; SNP = single nucleotide polymorphism.

‡ Only results of SNP pairs not strongly associated in the control population (*P*_interaction_ > .01 in control-only analyses) were included in the counts.

§ *P* value for difference between observed and expected numbers of tests, assuming each test is independent and that, under the null hypothesis, the observed number of statistically significant tests follows a poisson distribution. The statistical test was two-sided.

**||** Some SNPs were linked, as described in the Supplementary Methods (available online).

¶ Only the most statistically significant SNP from each group of linked SNPs were included in these analyses.

**Table 2.** Odds ratio for family history of breast cancer in first-degree relatives: unadjusted and adjusted by PRS and stratified by age

Age group	Unadjusted by PRS	Adjusted by PRS	% attenuation†
	OR* (95% CI)	OR (95% CI)	
All subjects	1.81 (1.69 to 1.93)	1.68 (1.56 to 2.86)	12.6%
<40 y	2.90 (2.07 to 4.07)	2.76 (1.96 to 3.89)	4.6%
40–60 y	1.88 (1.71 to 2.08)	1.72 (1.56 to 1.90)	14.1%
≥60 y	1.63 (1.47 to 1.82)	1.53 (1.37 to 1.70)	13.0%

* Odds ratio for developing breast cancer for women with a family history of breast cancer in a first-degree relative compared with women without a family history, adjusting for study and seven principal components. 21 865 breast cancer cases and 15 830 control women provided family history information. CI = confidence intervals; PRS = polygenic risk score; OR = odds ratio.

† Percent attenuation on log scale.

### Association Between PRS and Breast Cancer Risk

**Figure 1.**
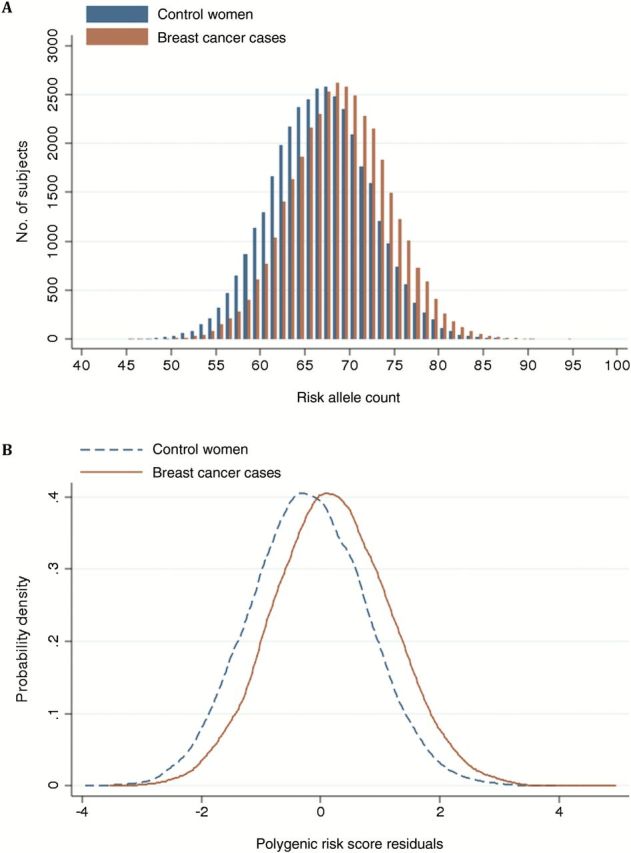
**Distribution of the number of breast cancer risk alleles (A**) and polygenic risk score residuals after adjusting the polygenic risk score (PRS) for study and seven principal components **(B)**, in 33 673 breast cancer cases and 33 381 control women of European origin. The PRS approximated a normal distribution in both breast cancer cases and control women. The mean PRS was 0.69 for breast cancer cases and 0.49 for control women. PRS residuals are standardized Pearson’s residuals calculated after regression of the score on seven principal components.

**Figure 2.**
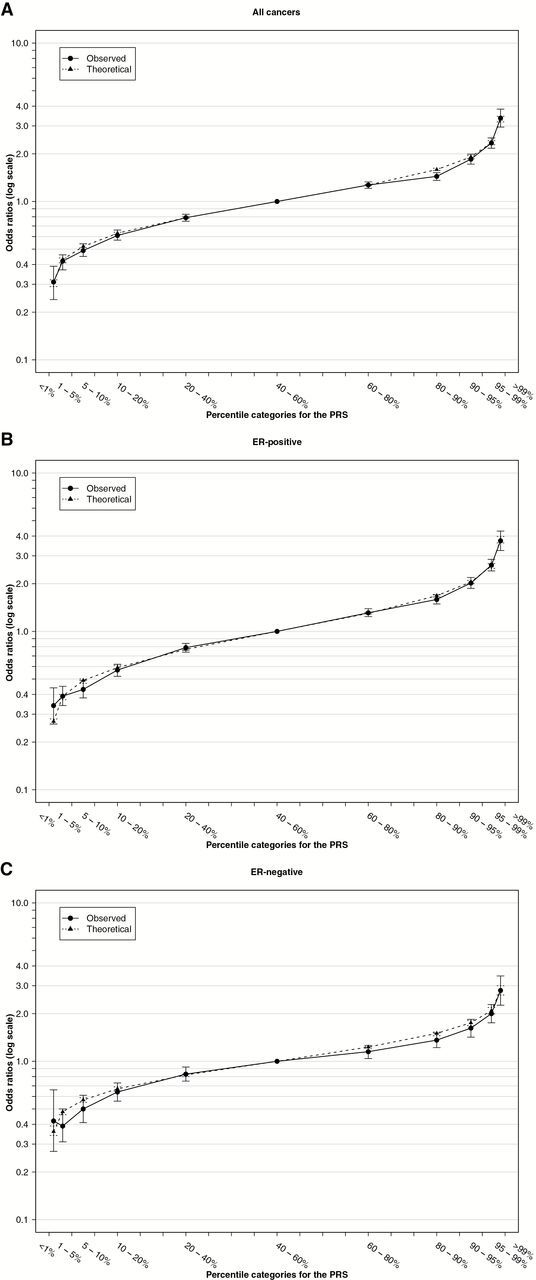
Association between the polygenic risk score (PRS) and breast cancer risk in women of European origin for **(A)** all breast cancers, **(B)** estrogen receptor (ER)–positive disease, and **(C)** ER-negative disease. Odds ratios are for different percentiles of the PRS relative to the middle quintile (40% to 60%) of the PRS. Odds ratios and 95% confidence intervals are shown. **Regular lines** denote the observed estimates, and **dotted lines** the theoretical estimates under a multiplicative polygenic model with a standard deviation of the PRS of 0.45 for all breast cancer, 0.50 for ER-positive breast cancer, and 0.38 for ER-negative breast cancer, as derived from the estimated effect sizes and allele frequencies/haplotype frequencies for each locus. PRS = polygenic risk score.

As predicted by the polygenic, multiplicative model, the number of breast cancer risk alleles and the 77-SNP PRS approximated a normal distribution for both breast cancer cases and control women (Figure 1). The odds ratios for developing breast cancer by percentiles of the PRS, compared with women in the middle quintile (40^th^ to 60^th^ percentile) are shown in Figure 2A. The observed odds ratios were similar to the odds ratios predicted under a polygenic multiplicative model; the 95% confidence interval (CI) included the predicted odds ratio at all points except the 80^th^ to 90^th^ percentile (Figure 2A; Supplementary Table 8, available online). For women in the lowest 1% of the PRS distribution, the estimated odds ratio compared with women in the middle quintile was 0.32 (95% CI = 0.25 to 0.40). By contrast, for women in the highest 1% of the PRS distribution, the estimated OR compared with women in the middle quintile was 3.36 (95% CI = 2.95 to 3.83, *P* = 7.5x10^-74^). When PRS were derived separately for ER-positive and ER-negative disease, the corresponding odds ratios were 3.73 (95% CI = 3.24 to 4.30) and 2.80 (95% CI = 2.26 to 3.46), respectively (Figure 2, B and C). The log OR per unit standard deviation of the PRS was 0.44 (95% CI = 0.42 to 0.46) for overall breast cancer, 0.49 (95% CI = 0.47 to 0.51) for ER-positive, and 0.37 (95% CI = 0.34 to 0.40) for ER-negative disease (Table 3). A validation analysis including only one large study (pKARMA) that was not part of any SNP discovery analyses found similar odds ratio estimates to those in the remaining studies, except for the 60% to 80% and 90% to 95% categories, for which estimates were higher in pKARMA (Table 4; Supplementary Table 9, available online). The log OR per unit SD was also similar for pKARMA alone (log OR per unit SD = 0.4).

**Table 3.** Association between PRS and breast cancer risk in different age groups

	All breast cancers	ER-positive disease	ER-negative disease
Age group*	log OR† (95% CI)	log OR (95% CI)	log OR (95% CI)
All ages	0.44 (0.42 to 0.46)	0.49 (0.47 to 0.51)	0.37 (0.34 to 0.40)
<40 y	0.46 (0.38 to 0.53)	0.56 (0.47 to 0.65)	0.48 (0.36 to 0.59)
40–49 y	0.46 (0.42 to 0.50)	0.53 (0.48 to 0.57)	0.36 (0.29 to 0.43)
50–59 y	0.48 (0.45 to 0.51)	0.54 (0.50 to 0.57)	0.37 (0.32 to 0.43)
≥60 y	0.41 (0.38 to 0.43)	0.44 (0.41 to 0.47)	0.36 (0.31 to 0.42)
	Interaction OR‡ (95% CI)	Interaction OR (95% CI)	Interaction OR (95% CI)
Interaction between PRS and age	0.98 (0.96 to 0.99)	0.97 (0.95 to 0.98)	0.94 (0.91 to 1.00)
*P*_interaction_	.005	1.08x10^-5^	.06

* Age of breast cancer cases (age at diagnosis) and control women (age at interview). CI = confidence intervals; PRS = polygenic risk score; log OR = log odds ratio.

† log OR for association between the PRS coded as a continuous variable and breast cancer risk (per unit SD of the PRS)

‡ OR per 10 years for interaction between PRS and age.

**Table 4.** Validation analyses in the pKARMA study*

Percentile of PRS, %	All studies in iCOGS excluding pKARMA	pKARMA only
	OR† (95% CI)	OR (95% CI)
<1	0.29 (0.23 to 0.37)	0.48 (0.28 to 0.83)
>1–5	0.42 (0.37 to 0.47)	0.48 (0.36 to 0.63)
5–10	0.55 (0.50 to 0.61)	0.58 (0.45 to 0.74)
10–20	0.65 (0.60 to 0.70)	0.68 (0.57 to 0.81)
20–40	0.80 (0.76 to 0.85)	0.81 (0.71 to 0.94)
40–60	1 (referent)	1 (referent)
60–80	1.18 (1.12 to 1.24)	1.35 (1.19 to 1.54)
80–90	1.48 (1.39 to 1.57)	1.56 (1.34 to 1.82)
90–95	1.69 (1.56 to 1.82)	2.05 (1.70 to 2.47)
95–99	2.20 (2.03 to 2.38)	2.12 (1.73 to 2.59)
>99	2.81 (2.43 to 3.24)	3.06 (2.16 to 4.34)

* Comparison of effect sizes (odds ratios) by percentile of the polygenic risk score (PRS) in pKARMA (not included in the discovery set) and in all other studies (included in the discovery set). The pKARMA study comprises 4553 breast cancer cases and 5537 control women. Only single nucleotide polymorphisms (SNPs) that reached genome-wide statistical significance in a meta-analysis of iCOGS and previous combined genome-wide association studies were included in the risk score, and the effect sizes for each SNP were estimated using iCOGS database minus pKARMA (Supplementary Table 9, available online). PRS = polygenic risk score; OR = odds ratio.

† Odds ratios are for different percentiles of the polygenic PRS relative to the middle quintile (40% to 60%) of the PRS.

The associations between PRS and breast cancer in different age groups are summarized in Table 3 and Supplementary Figure 2 (available online). There was a statistically significant interaction between PRS and age, the association between PRS and breast cancer risk decreasing with age (Table 3).

A family history of breast cancer in one or more affected first-degree relatives was reported by 18.5% of breast cancer cases and 11.1% of control women. The odds ratio for family history was attenuated from 1.81 to 1.68 (12.6% attenuation) after adjusting for the PRS (Table 2). At younger ages (<40 years), there was less attenuation (from 2.90 to 2.76, 4.6% attenuation) (Table 2). The joint effects of the PRS and family history were largely consistent with a multiplicative model (*P*_interaction_ = .34 for the interaction between the PRS and family history; data not shown); however, we observed a stronger effect of family history for women at the lowest 1% of the PRS (Supplementary Table 10, available online).

The discriminative accuracy of the PRS, as measured by the C-statistic, was 0.622 (95% CI = 0.619 to 0.627); discrimination was similar when restricted to pKARMA alone, with an area under the curve of 0.615 (95% CI = 0.608 to 0.616) (data not shown).

### Absolute Risks of Developing Breast Cancer by Levels of PRS

**Figure 3.**
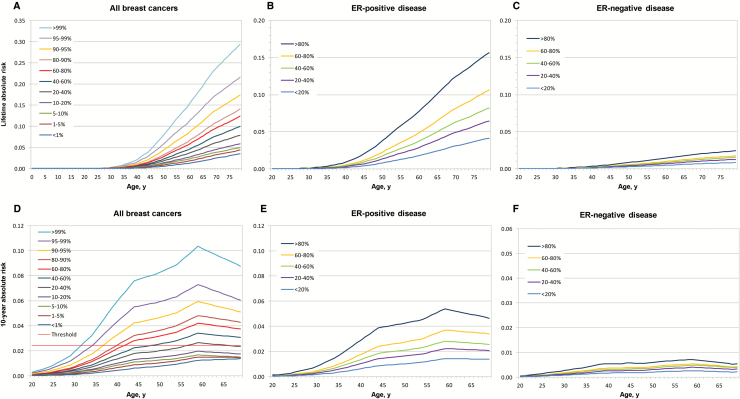
Cumulative and 10-year absolute risks of developing breast cancer for women of European origin by percentiles of the polygenic risk score (PRS). Cumulative absolute risk of developing breast cancer for **(A)** all breast cancers, **(B)** estrogen receptor (ER)–positive disease, and **(C)** ER-negative disease by percentiles of the PRS; and 10-year absolute risk of developing breast cancer for **(D)** all breast cancers, **(E)** ER-positive disease, and **(F)** ER-negative disease. Note different scales and PRS categories in the different panels. The **red line** shows the 2.4% risk threshold corresponding to the risk for women age 47 years who were eligible for screening, calculated as described in the Supplementary Methods (available online). Absolute risks were calculated using the PRS relative risks estimated as described in the Supplementary Methods (available online), and breast cancer incident rates and mortality from other causes obtained from the UK National Office for Statistics. For subtype-specific disease, the absolute risk for women in a particular PRS category for ER-positive disease and another PRS category for ER-negative disease were calculated. Information on proportions of tumors by ER status was obtained from the West Midlands Registry.

**Figure 4.**
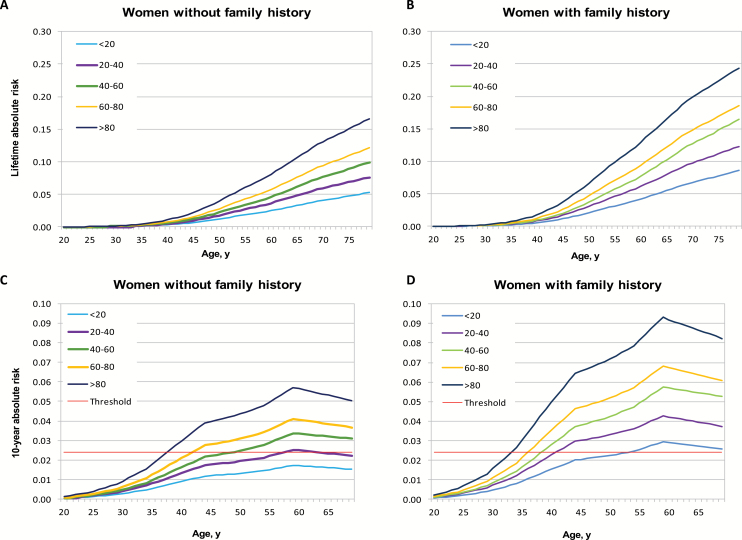
Cumulative and 10-year absolute risks of developing breast cancer for women of European origin with and without a family history of breast cancer by percentiles of the polygenic risk score (PRS). Cumulative absolute risk of developing breast cancer for women **(A)** without a family history and **(B)** with a family history, and 10-year absolute risk of developing breast cancer for women **(C)** without a family history, and **(D)** with a family history of breast cancer by percentiles of the PRS. The **red line** shows the 2.4% risk threshold corresponding to the risk for women age 47 years who were eligible for screening. Absolute risks were calculated using PRS relative risks estimated as described in Methods, and breast cancer incident rates and mortality from other causes obtained from the UK National Office for Statistics.

The estimated risk of developing breast cancer by age 80 years for women in the lowest and highest 1% of the PRS was 3.5% (95% CI = 2.6% to 4.4%) and 29.0% (95% CI = 24.9% to 33.5%), respectively (Figure 3A). For the lowest and highest quintiles of the PRS, the risk was 5.3% (95% CI = 5.1% to 5.7%) and 17.2% (95% CI = 16.1% to 18.1%), respectively (data not shown). The corresponding risks of developing ER-positive disease were 4.1% and 15.7% for women in the lowest and highest quintiles, respectively, of the ER-positive PRS (averaged over all ER-negative PRS categories), whereas the highest lifetime risk for ER-negative disease was 2.4% (women in the highest quintile of ER-negative PRS and average ER-positive risk) (Figure 3). Lifetime risk of breast cancer for women in the lowest and highest quintiles of the PRS were 5.2% and 16.6% for a woman without family history and 8.6% and 24.4% for a woman with a first-degree family history of breast cancer (Figure 4).

We estimated the 10-year absolute risk of breast cancer at different ages and evaluated the age at which women at different levels of the PRS reach a threshold of 2.4%, which corresponds to the average 10-year risk of breast cancer for women age 47 years. This threshold was reached at 32 years for women whose PRS is above the 99th percentile of the PRS, and 57 years for women in the 20th to 40th percentiles of the PRS, and was never reached for women in lower percentiles (Figure 3D). As expected, lifetime risks were higher, and the ages at which the 2.4% threshold was reached were lower for women with a family history of breast cancer (Figure 4).

## Discussion

In this report, we evaluated the degree of breast cancer risk stratification that can be attained in women of European ancestry using data for 77 common genetic variants, summarized as a PRS. Our results show that the PRS stratifies breast cancer risk in women without family history and refines genetic risk in women with a family history of breast cancer.

The PRS we used (sum of the minor alleles weighted by the per-allele log OR) is the most efficient, assuming that SNP odds ratios combine multiplicatively (ie, no interactions on a log-additive scale) (18). Evaluation of pairwise SNP interactions showed that this was a reasonable assumption. Although no individual interactions could be established, we observed an excess of multiplicative interactions at *P* less than .01. This could be the result of underlying population stratification not accounted for by principal components adjustment or reflect the presence of multiple interactions too weak to be established individually. A recent study also found no evidence for interactions among SNPs with weaker evidence for main effects (19). Although we did not test for higher order interactions among SNPs, consistency between empirical and predicted odds ratios assuming multiplicative effects suggests that across all possible multiway interactions the overall effect is close to multiplicative.

The 77-SNP PRS was associated with a larger effect than previously reported for a 10-SNP PRS (20). For example, our odds ratio for breast cancer for women in the highest compared with the middle quintile was 1.82 (95% CI = 1.73 to 1.90) vs 1.44 (95% CI = 1.35 to 1.53) for the 10-SNP PRS (20). A potential concern is that the PRS was constructed using iCOGS data that were, in part, the basis for discovery of many of the loci. This could lead to some upward bias in the odds ratio estimates (winner’s curse); however, analyses based on a large study (pKARMA) that was not part of any discovery set obtained similar estimates indicating that any winner’s curse effect is likely to be small.

There has been little evidence of differences by age in the per-allele odds ratio for individual SNPs. However, we observed a small but statistically significant decrease in odds ratio for PRS with increasing age. As expected, the odds ratio for family history was reduced after adjustment for the PRS. This attenuation (~12.6%) was consistent with the estimated fraction of the two-fold FRR explained by the 77-SNPs under a polygenic risk model (15). The joint effects of PRS and family history were consistent with a multiplicative model. A stronger FRR was observed for women at the lowest percentile of the PRS, but this was based on small numbers and requires confirmation. The degree of attenuation of the family history odds ratio was lower below age 40 years, as a result of the higher FRR at young ages, suggesting that rarer genetic variants may be more important at young ages.

We calculated the absolute risk of developing breast cancer for women at different levels of genetic risk according to the PRS. The lifetime risk for women below the first and above the 99^th^ percentile of the PRS was 3.5% (95% CI = 2.6% to 4.4%) and 29.0% (95% CI = 24.9% to 33.5%), respectively. UK NICE guidelines recommend enhanced surveillance for women with a family history with lifetime risk of developing breast cancer over 17% (21). Figure 3 indicates that the PRS alone could identify approximately 8% of all women in the UK population at this level of risk, regardless of family history or other risk factors; approximately 17% of all breast cancer cases in the population would be expected to occur among these women. By contrast, the low absolute risk of breast cancer among women at the lowest end of the risk distribution raises the possibility that such women might be recommended more limited surveillance. Women at different levels of the PRS reach the same 10-year risk threshold at different ages, supporting the notion that using SNP profiles rather than age alone as a criterion to offer routine mammographic screening could lead to more effective screening programs (6). The utility of such an approach would, however, depend on the acceptability of risk-based surveillance, together with health economic considerations.

Prediction of subtype-specific breast cancer should also be informative for prevention (4). Recently updated NICE guidelines include recommendations to use endocrine treatments (tamoxifen and raloxifene) for primary prevention of breast cancer for women at moderate to high risk (21). These guidelines are based on risk of overall breast cancer for women with a family history of breast cancer. However, because these drugs prevent only ER-positive tumours, risk estimates incorporating the ER-positive PRS could better define the subset of women most likely to benefit. Our sample was derived from studies in Europe, North America, and Australia and restricted to women of European origin. While the results should be widely applicable in these populations, additional studies will be required to develop and validate genetic profiles for other populations, in particular Asian and African populations, where SNP associations, background incidence rates and distribution of tumour characteristics are substantially different.

Our analysis summarized family history in terms of a single binary variable, but familial risk of breast cancer also depends on the number of affected and unaffected relatives and their ages. Risk prediction algorithms that combine full family history data with a polygenic component perform better than simpler models (22). It is possible to incorporate the current PRS into family-history based models for breast cancer, such BOADICEA, to improve genetic risk prediction (23).

The COGS project includes the largest set of breast cancer studies with both phenotype and genotype information, and our analysis utilized by far the largest number of SNPs with confirmed associations with breast cancer, including all SNPs discovered to date. Further refinement of the risk stratification should be possible through incorporating additional SNPs exhibiting evidence for association, but not at formal genome-wide statistical significance, together with variants in genes conferring intermediate or high risk (15).

The risk discrimination provided by the genetic profile, summarised in the PRS and family history, should be further improved by combining, with lifestyle risk factors, benign breast disease, and mammographic density (24,25,28). Although we did not consider lifestyle factors explicitly in this dataset, other large studies have found no good evidence for interactions between common susceptibility SNPs and lifestyle factors for breast cancer, suggesting that SNPs generally combined multiplicatively (26,27). Darabi et al. (25) estimated a C-statistic of 0.60 for lifestyle risk factors including mammographic density. By comparison, we estimated the C-statistic for the PRS to be 0.62. Assuming that the multiplicative model is correct, the C-statistic would increase to 0.66 with the addition of the lifestyle risk factors. If modifiable risk factors and the PRS act multiplicatively, targeting public health interventions to women at higher genetic risk should result in a larger absolute risk reduction. For example, the decision to prescribe hormone replacement therapy might be guided by the PRS (28). Similar considerations would apply to risk-reducing interventions such as preventive medication and oophorectomy.

Some limitations of this study should be noted. Although the study was extremely large, the numbers of breast cancer cases and control women were still too limited to provide precise estimates of relative risks in the extremes of the PRS (for example, the highest 1%). Numbers were also limited to explore the effects at very young ages, and estimates were less precise for ER-negative disease. There was heterogeneity among the studies, both in population and design, but we saw no evidence of heterogeneity in SNP odds ratios among studies, suggesting that the estimates should be broadly applicable. Oversampling for family history could have led to a bias in the odds ratios by PRS, and for this reason we excluded studies that were sampled on the basis of family history. Finally, we were not able to consider lifestyle/environmental risk factors in our model, as data on all of these risk factors were not consistently available across all studies. Interactions between the PRS and environmental factors will need to be explicitly tested for in future studies.

In previous reports, improvement in risk discrimination by genomic profiling over that conferred by known risk factors was not substantial (24,29), although better discrimination was obtained for certain subgroups of women (30,31). Previous analyses, however, were based on a much smaller set of SNPs than included in this report. This study provides precise empirical estimates of the combined effects of multiple SNPs and the level of risk stratification possible. These estimates may inform the debate on public health utility and implementation of the PRS in clinical practice. Our work suggests that the PRS, particularly when used in combination with other risk factors, could help identify subsets of women at different levels of risk, for whom management would differ. The PRS may facilitate early detection of cancers in younger women and, importantly, identify individuals at risk of specific subtypes of breast cancer. Finally, there is potential for a stronger impact in modifying environmental factors in women at higher risk of breast cancer. Prospective analyses of the 77 SNP PRS, in combination with other risk factors, will be required to validate the overall accuracy of risk prediction. Such a comprehensive risk prediction algorithm could provide a powerful basis for stratified breast cancer prevention programs.

## Funding

This work was supported by Cancer Research-UK (grant numbers C1287/A10118, C1287/A12014) and the European Community’s Seventh Framework Programme (223175 [HEALTH-F2-2009–223175]) (COGS). Genotyping of the iCOGS array was funded by the European Union (HEALTH-F2-2009–223175), Cancer Research UK (C1287/A10710), the Canadian Institutes of Health Research (CIHR) for the “CIHR Team in Familial Risks of Breast Cancer” program, and the Ministry of Economic Development, Innovation and Export Trade of Quebec (PSR-SIIRI-701). This work was also supported by Breakthrough Breast Cancer funding (to MGC). Analysis was supported in part by the National Institutes of Health Post-Genome Wide Association initiative (1U19CA148065 (DRIVE) and 1U19CA148537 (ELLIPSE)). Laboratory infrastructure was funded by Cancer Research UK (C8197/A10123). This work was also supported by the Government of Canada through Genome Canada and the Canadian Institutes of Health Research and the Ministère de l’enseignement supérieur, de la recherche, de la science et de la technologie du Québec through Génome Québec for the PERSPECTIVE project. Breast Cancer Association Consortium meetings were funded by the European Union European Cooperation in Science and Technology (COST) programme (BM0606).

The Australian Breast Cancer Family Study (ABCFS), Northern California Breast Cancer Family Registry (NC-BCFR) and Ontario Familial Breast Cancer Registry (OFBCR) studies were supported by the US National Cancer Institute (UM1 CA164920). The ABCFS was also supported by the National Health and Medical Research Council of Australia, the New South Wales Cancer Council, the Victorian Health Promotion Foundation (Australia), and the Victorian Breast Cancer Research Consortium. JLH is a National Health and Medical Research Council (NHMRC) Australia Fellow and a Victorian Breast Cancer Research Consortium Group Leader. MCS is an NHMRC Senior Research Fellow and a Victorian Breast Cancer Research Consortium Group Leader. JLH and MCS are both group leaders of the Victoria Breast Cancer Research Consortium. The content of this manuscript does not necessarily reflect the views or policies of the US National Cancer Institute or any of the collaborating centers in the Breast Cancer Family Registry (BCFR), nor does mention of trade names, commercial products, or organizations imply endorsement by the US Government or the BCFR.

The Amsterdam Breast Cancer Study (ABCS) was supported by the Dutch Cancer Society (NKI 2007–3839; 2009 4363) and BBMRI-NL, which is a Research Infrastructure financed by the Dutch government (NWO 184·021·007).

The Australian Breast Cancer Tissue Bank (ABCTB) study was supported by the National Health and Medical Research Council of Australia, The Cancer Institute New South Wales and the National Breast Cancer Foundation.

The Bavarian Breast Cancer Cases and Controls (BBCC) study was partly funded by ELAN-Fond of the University Hospital of Erlangen.

The British Breast Cancer Study (BBCS) was funded by Cancer Research UK and Breakthrough Breast Cancer and acknowledges National Health Service funding to the National Institutes for Health Research Biomedical Research Centre, and the National Cancer Research Network (NCRN). The BBCS GWAS received funding from The Institut National de Cancer.

The Breast Cancer In Galway Genetic Study (BIGGS) was supported by National Institutes for Health Research Comprehensive Biomedical Research Centre, Guy’s & St.Thomas’ NHS Foundation Trust in partnership with King’s College London, United Kingdom (ES), and the Oxford Biomedical Research Centre (IT).

The Breast Cancer Study of the University Clinic Heidelberg (BSUCH) was supported by the Dietmar-Hopp Foundation, the Helmholtz Society, and the German Cancer Research Center (DKFZ).

The CECILE Breast Cancer Study (CECILE) was funded by Fondation de France, Institut National du Cancer (INCa), Ligue Nationale contre le Cancer, Ligue contre le Cancer Grand Ouest, Agence Nationale de Sécurité Sanitaire (ANSES), Agence Nationale de la Recherche (ANR).

The Copenhagen General Population Study (CGPS) was supported by the Chief Physician Johan Boserup and Lise Boserup Fund, the Danish Medical Research Council, and Herlev Hospital.

The Spanish National Cancer Centre Breast Cancer Study (CNIO-BCS) was supported by the Genome Spain Foundation, the Red Temática de Investigación Cooperativa en Cáncer, and by grants from the Asociación Española Contra el Cáncer and the Fondo de Investigación Sanitario (PI11/00923, PI081120).

The California Teachers Study (CTS) was initially supported by the California Breast Cancer Act of 1993 and the California Breast Cancer Research Fund (contract 97-10500) and is currently funded through the National Institutes of Health (R01 CA77398). The CTS study was also funded by the Lon V. Smith Foundation (LVS39420) to HAC. Collection of cancer incidence data was supported by the California Department of Public Health as part of the statewide cancer reporting program mandated by California Health and Safety Code Section 103885.

For the DietCompLyf Breast Cancer Survival Study (DBCSS) the University of Westminster curated the DietCompLyf database, created by and funded by Against Breast Cancer Registered Charity No. 1121258. The University of Westminster’s Against Breast Cancer Research Unit acknowledges funding from the charity Against Breast Cancer (Registered Charity Number 1121258).

The Esther Breast Cancer Study (ESTHER) was supported by a grant from the Baden Württemberg Ministry of Science, Research and Arts. Additional cases were recruited in the context of the VERDI study, which was supported by a grant from the German Cancer Aid (Deutsche Krebshilfe).

The Familial Breast Cancer Study (FBCS) study was supported by funds from Cancer Research UK (C8620/A8372, C8620/A8857), a US Military Acquisition (ACQ) Activity, an Era of Hope Award (W81XWH-05-1-0204), and the Institute of Cancer Research UK. CT is funded by a Medical Research Council (UK) Clinical Research Fellowship. The FBCS acknowledges National Health Service (NHS) funding to the Royal Marsden / Institute of Cancer Research National Institutes for Health Research (NIHR) Specialist Cancer Biomedical Research Centre.

The German Consortium for Hereditary Breast & Ovarian Cancer (GC-HBOC) was supported by Deutsche Krebshilfe (107 352).

Gene Environment Interaction and Breast Cancer in Germany (GENICA) was funded by the Federal Ministry of Education and Research (BMBF) Germany (01KW9975/5, 01KW9976/8, 01KW9977/0, 01KW0114), the Robert Bosch Foundation, Stuttgart, Deutsches Krebsforschungszentrum (DKFZ), Heidelberg, Institute for Prevention and Occupational Medicine of the German Social Accident Insurance (IPA), Bochum, and the Department of Internal Medicine, Evangelische Kliniken Bonn gGmbH, Johanniter Krankenhaus, Bonn, Germany.

The Genetic Epidemiology Study of Breast Cancer by Age 50 (GESBC) study was supported by the Deutsche Krebshilfe e. V. (70492) and the German Cancer Research Center (DKFZ).

The Hannover Breast Cancer Study (HABCS) was supported by an intramural grant from Hannover Medical School.

The Helsinki Breast Cancer Study (HEBCS) was financially supported by the Helsinki University Central Hospital Research Fund, Academy of Finland (grant number 266528), the Finnish Cancer Society, the Nordic Cancer Union, and the Sigrid Juselius Foundation.

The Hannover-Minsk Breast Cancer Study (HMBCS) was supported by a grant from the Friends of Hannover Medical School and by the Rudolf Bartling Foundation.

The Hannover-Ufa Breast Cancer Study (HUBCS) was supported by a grant from the German Federal Ministry of Research and Education (RUS08/017) and by the Ministry of Education and Science of the Russian Federation (number 14.574.21.0026, agreement dated June 17, 2014, a unique identifier agreement RFMEFI57414X0026), the Russian Foundation for Basic Research (14-04-31169 mol_a) and State task of the Ministry of Education and Science of the Russian Federation (310-14).

The Karolinska Breast Cancer Study (KARBAC) was supported by the regional agreement on medical training and clinical research (ALF) between Stockholm County Council and Karolinska Institutet, the Swedish Cancer Society, the Gustav V. Jubilee foundation, and the Bert von Kantzows foundation.

The Kuopio Breast Cancer Project (KBCP) was supported by the special Government Funding (EVO) of Kuopio University Hospital grants, Cancer Fund of North Savo, the Finnish Cancer Organizations, the Academy of Finland, and by the strategic funding of the University of Eastern Finland.

The kConFab study was supported by the National Breast Cancer Foundation and previously by the National Health and Medical Research Council, the Queensland Cancer Fund, the Cancer Councils of New South Wales, Victoria, Tasmania, and South Australia, and the Cancer Foundation of Western Australia. The kConFab Clinical Follow Up Study was funded by the National Health and Medical Research Council (145684, 288704, 454508). RB was a Cancer Institute NSW Fellow.

The AOCS study was supported by the United States Army Medical Research and Materiel Command (DAMD17-01-1-0729), the Cancer Council of Tasmania, the Cancer Foundation of Western Australia and the National Health and Medical Research Council (199600), and a National Health and Medical Research Council grant to GCT.

The Leuven Multidisciplinary Breast Centre (LMBC) study is supported by the “Stichting tegen Kanker” (232–2008, 196–2010) and by the FWO and the KULPFV/10/016-SymBioSysII to DL.

The Mammary Carcinoma Risk Factor Investigation (MARIE) study was supported by the Deutsche Krebshilfe e.V. (70-2892-BR I, 106332, 108253, 108419), the Hamburg Cancer Society, the German Cancer Research Center (DKFZ), and the Federal Ministry of Education and Research (BMBF) Germany (01KH0402).

The Milan Breast Cancer Study Group (MBCSG) was supported by grants from the Italian Association for Cancer Research (AIRC) and by funds from the Italian citizens who allocated the 5/1000 share of their tax payment in support of the Fondazione IRCCS Istituto Nazionale Tumori, according to Italian laws (INT-Institutional strategic projects “5x1000”).

The Mayo Clinic Breast Cancer Study (MCBCS) was supported by the National Institutes of Health (CA122340, CA128978), a National Institutes of Health Specialized Program of Research Excellence (SPORE) in Breast Cancer (CA116201), the Breast Cancer Research Foundation, the Komen Race for the Cure, and by a generous gift from the David F. and Margaret T. Grohne Family Foundation and the Ting Tsung and Wei Fong Chao Foundation.

The Melbourne Collaborative Cohort Study (MCCS) cohort recruitment was funded by VicHealth and Cancer Council Victoria. The MCCS was further supported by Australian National Health and Medical Research Council (209057, 251553, 504711) and by infrastructure provided by Cancer Council Victoria.

The Multi-ethnic Cohort (MEC) was supported by the National Institutes of Health (CA63464, CA54281, CA098758, and CA132839).

The Memorial Sloan-Kettering Cancer Center (MSKCC) was supported by grants from the Breast Cancer Research Foundation and the Robert and Kate Niehaus Clinical Cancer Genetics Initiative.

The work of Montreal Gene-Environment Breast Cancer Study (MTLGEBCS) was supported by the Quebec Breast Cancer Foundation, the Canadian Institutes of Health Research for the “CIHR Team in Familial Risks of Breast Cancer” program (CRN-87521), and the Ministry of Economic Development, Innovation and Export Trade (PSR-SIIRI-701). MG received an Investigator Award from the CIHR and a Health Scholar Award from the Fonds de la recherche en santé du Québec. J Simard is chairholder of the Canada Research Chair in Oncogenetics.

The Norwegian Breast Cancer Study (NBCS) was supported by grants from the Norwegian Research council (155218/V40, 175240/S10 to ALBD, FUGE-NFR 181600/V11) to VNK and a Swizz Bridge Award to ALBD.

The Nashville Breast Health Study (NBHS) was supported by National Institutes of Health (grant R01CA100374).

The Nurses Health Study (NHS) was funded by National Institutes of Health (PO1 CA87969), and this project was (in part) supported by the Genetic Associations and Mechanisms in Oncology (GAME-ON) Network (U19 CA148065).

The Oulu Breast Cancer Study (OBCS) was supported by research grants from the Finnish Cancer Foundation, the Academy of Finland, the University of Oulu, and the Oulu University Hospital.

The Leiden University Medical Centre Breast Cancer Study (ORIGO) was supported by the Dutch Cancer Society (RUL 1997-1505) and the Biobanking and Biomolecular Resources Research Infrastructure (BBMRI-NL CP16).

The NCI Polish Breast Cancer Study (PBCS) was supported by the Intramural Research Programs of the Division of Cancer Epidemiology and Genetics and the Center for Cancer Research of the National Cancer Institute.

The Karolinska Mammography Project for Risk Prediction of Breast Cancer (pKARMA) study was supported by Märit and Hans Rausings Initiative Against Breast Cancer and Cancer Risk Prediction Center, a Linneus Centre (contract 70867902) financed by the Swedish Research Council.

The Prospective Study of Outcomes in Sporadic Versus Hereditary Breast Cancer (POSH) study was supported by Cancer Research UK (A7572, A11699, C22524).

The Rotterdam Breast Cancer Study (RBCS) was funded by the Dutch Cancer Society (DDHK 2004–3124, DDHK 2009–4318).

The Singapore and Sweden Breast Cancer Study (SASBAC) was supported by the Agency for Science, Technology and Research of Singapore (A*STAR), the National Institutes of Health, and the Susan G. Komen Breast Cancer Foundation.

The Sheffield Breast Cancer Study (SBCS) was supported by Yorkshire Cancer Research (S295, S299, S305PA) and the Sheffield Experimental Cancer Medicine Centre.

The Southern Community Cohort Study (SCCS) is funded by National Institutes of Health (R01 CA092447). The Arkansas Central Cancer Registry is fully funded by a grant from the National Program of Cancer Registries, Centers for Disease Control and Prevention (CDC).

Study of Epidemiology and Risk factors in Cancer Heredity (SEARCH) was funded by a programme grant from Cancer Research UK (C490/A10124), the UK National Institute for Health Research Biomedical Research Centre at the University of Cambridge, and a Cancer Research UK grant (C8197/A10123) to AMD.

The Städtisches Klinikum Karlsruhe Deutsches Krebsforschungszentrum Study (SKKDKFZS) was supported by the Deutsches Krebsforschungszentrum (DKFZ).

The IHCC-Szczecin Breast Cancer Study (SZBCS) was supported by grant (PBZ_KBN_122/P05/2004).

The Triple Negative Breast Cancer Consortium Study (TNBCC) was supported by the National Institutes of Health (CA128978) and the National Institutes of Health Specialized Program of Research Excellence in Breast Cancer (CA116201), the Breast Cancer Research Foundation, a generous gift from the David F. and Margaret T. Grohne Family Foundation and the Ting Tsung and Wei Fong Chao Foundation, the Stefanie Spielman Breast Cancer fund and the Ohio State University (OSU) Comprehensive Cancer Center, DBBR (a CCSG Share Resource by National Institutes of Health Grant P30 CA016056), the European Union (European Social Fund – ESF) and Greek national funds through the Operational Program “Education and Lifelong Learning” of the National Strategic Reference Framework (NSRF) - Research Funding Program of the General Secretariat for Research & Technology: ARISTEIA.

The DEMOKRITOS study was supported by a Hellenic Cooperative Oncology Group research grant (HR R_BG/04) and the Greek General Secretary for Research and Technology (GSRT) Program, Research Excellence II, funded at 75% by the European Union.

The OSU study was funded by the Stefanie Spielman fund and the OSU Comprehensive Cancer Center.

The Roswell Park Cancer Institute (RPCI) study was supported by RPCI DataBank and BioRepository (DBBR), a Cancer Center Support Grant Shared Resource (P30 CA016056-32).

The UCIBCS was supported by the National Institutes of Health, National Cancer Institute grant CA-58860 and the Lon V Smith Foundation grant LVS-39420.

The UK Breakthrough Generations Study (UKBGS) was funded by Breakthrough Breast Cancer and the Institute of Cancer Research (ICR). ICR acknowledges NHS funding to the Royal Marsden Hospital/ICR National Institutes for Health Research Biomedical Research Centre.

The US Three State Study (US3SS) was supported by Massachusetts (R01CA47305 to KME), Wisconsin (R01 CA47147 to PAN), and New Hampshire (R01CA69664 to LTE) centers and Intramural Research Funds of the National Cancer Institute, Department of Health and Human Services.

The US Radiologic Technologists Study (USRT) was funded by the Intramural Research Program of the Division of Cancer Epidemiology and Genetics, National Cancer Institute, National Institutes of Health, US Department of Health and Human Services.

Biological sample preparation for several studies was conducted at the Epidemiology Biospecimen Core Lab, supported in part by the Vanderbilt-Ingram Cancer Center (P30 CA68485).

## Supplementary Material

Supplementary Data

## Notes

Author contributions: NM, DFE, and MG analyzed data relating to this manuscript and drafted the initial manuscript. DFE coordinated the BCAC and led the iCOGS genotyping. PH led the COGS collaboration. KM performed statistical analyses of the iCOGS data, with assistance from JT. JD provided bioinformatics support. MKB and QW coordinated the BCAC database. The remaining authors led individual studies and contributed to the design of the study, data collection, and revising the manuscript.

The sponsors had no role in the study design, in the collection, analysis, or interpretation of data, in the writing of the report, or in the decision to submit the paper for publication.

We extend our thanks to the many women who generously took part in these studies. We also thank all the researchers, nurses, clinicians, technicians, and administrative staff who have enabled this work to be carried out. In particular, we thank: Andrew Lee, Ed Dicks, and the staff of the Centre for Genetic Epidemiology Laboratory, the staff of the CNIO genotyping unit, Sylvie LaBoissière and Frederic Robidoux and the staff of the McGill University and Génome Québec Innovation Centre, the staff of the Copenhagen DNA laboratory, and Julie M. Cunningham, Sharon A. Windebank, Christopher A. Hilker, Jeffrey Meyer and the staff of Mayo Clinic Genotyping Core Facility.

The authors would also like to thank the West Midlands Cancer Intelligence Unit (WMCIU) for providing data on breast cancer incidence by ER status for 2010. As of 1st April 2013, WMCIU are part of Public Health England.

The ABCFS study thanks Maggie Angelakos, Judi Maskiell, and Gillian Dite.

The OFBCR study thanks Teresa Selander and Nayana Weerasooriya.

The ABCS study acknowledges Sanquin Research.

ABCTB Investigators include Christine Clarke, Rosemary Balleine, Robert Baxter, Stephen Braye, Jane Carpenter, Jane Dahlstrom, John Forbes, Soon Lee, Debbie Marsh, Adrienne Morey, Nirmala Pathmanathan, Rodney Scott, Allan Spigelman, Nicholas Wilcken, and Desmond Yip.

The BBCC study thanks Sonja Oeser, Silke Landrith, and Matthias Rübner.

The BBCS study thanks Eileen Williams, Elaine Ryder-Mills, and Kara Sargus.

The BIGGS study thanks Niall McInerney, Gabrielle Colleran, Andrew Rowan, and Angela Jones.

The BSUCH study thanks Peter Bugert and the Medical Faculty, Mannheim.

The CGPS study thanks the staff and participants of the Copenhagen General Population Study and Dorthe Uldall Andersen, Maria Birna Arnadottir, Anne Bank, and Dorthe Kjeldgård Hansen for excellent technical assistance. The Danish Breast Cancer Group (DBCG) is acknowledged for tumour information.

The CNIO-BCS study acknowledges the support of Charo Alonso and the Human Genotyping-CEGEN Unit (CNIO).

The CTS Steering Committee includes Leslie Bernstein, James Lacey, Sophia Wang, Huiyan Ma, Yani Lu, Jane Sullivan-Halley, and Jessica Clague DeHart at the Beckman Research Institute of the City of Hope, Dennis Deapen, Rich Pinder, Eunjung Lee, and Fred Schumacher at the University of Southern California, Pam Horn-Ross, Christina Clarke Dur, Peggy Reynolds, and David Nelson at the Cancer Prevention Institute of California, and Hannah Park at the University of California Irvine.

The FBCS study thanks the Wellcome Trust Case Control Consortium (see the WTCCC website for a full list of contributing investigators).

The HEBCS study thanks Kirsimari Aaltonen, Karl von Smitten, Sofia Khan, Tuomas Heikkinen, and Irja Erkkilä for their help with data and samples.

The HMBCS study thanks Johann H. Karstens and oncological centers in Belarus.

The KBCP study thanks Eija Myöhänen and Helena Kemiläinen.

kConFab/AOCS thanks Heather Thorne, Eveline Niedermayr, the AOCS Management Group (D. Bowtell, G. Chenevix-Trench, A. deFazio, D. Gertig, A. Green, P. Webb), and the ACS Management Group (A. Green, P. Parsons, N. Hayward, P. Webb, D. Whiteman).

The LMBC study thanks Gilian Peuteman, Dominiek Smeets, Thomas Van Brussel, and Kathleen Corthouts.

The MARIE study thanks Alina Vrieling, Katharina Buck, Ursula Eilber, Muhabbet Celik, and Sabine Behrens.

The MBCSG study acknowledges Bernard Peissel, Daniela Zaffaroni, Giulietta Scuvera, and Jacopo Azzolini of the Fondazione IRCCS Istituto Nazionale dei Tumori (INT); Bernardo Bonanni, Angela Maniscalco, Alessandra Rossi, Monica Barile, and Irene Feroce of the Istituto Europeo di Oncologia (IEO) and Loris Bernard and the personnel of the Cogentech Cancer Genetic Test Laboratory.

The MSKCC study thanks Marina Corines and Lauren Jacobs.

The MTLGEBCS study acknowledges the assistance of Lesley Richardson and Marie-Claire Goulet in conducting the study and would like to Martine Tranchant (Cancer Genomics Laboratory, CRCHUQ), Marie-France Valois, Annie Turgeon, and Lea Heguy (McGill University Health Center, Royal Victoria Hospital; McGill University) for DNA extraction, sample management, and skillful technical assistance.

The OBCS study thanks Saila Kauppila, Meeri Otsukka, and Kari Mononen.

The ORIGO study thanks E. Krol-Warmerdam and J. Blom for patient accrual, administering questionnaires, and managing clinical information.

The OSU study thanks Robert Pilarksi and Charles Shapiro, instrumental in the formation of the Breast Cancer Tissue Bank, and the Human Genetics Sample Bank for processing of samples. OSU Columbus area control specimens were provided by the Ohio State University’s Human Genetics Sample Bank.

The PBCS study thanks Dr. Mark Sherman of the National Cancer Institute (Bethesda, MD), Dr. Neonila Szeszenia-Dabrowska of the Nofer Institute of Occupational Medicine (Lodz, Poland), Dr. Witold Zatonski of the Department of Cancer Epidemiology and Prevention, the M. Sklodowska-Curie Cancer Center and Institute of Oncology (Warsaw, Poland), and Pei Chao and Michael Stagner from Information Management Services (Sliver Spring, MD) for their valuable contributions to the study.

The RBCS study thanks Petra Bos, Jannet Blom, Ellen Crepin, Anja Nieuwlaat, Annette Heemskerk, the Erasmus MC Family Cancer Clinic.

The SBCS study thanks Sue Higham, Helen Cramp, and Dan Connley.

Data on SCCS breast cancer cases used in this publication were provided by the Alabama Statewide Cancer Registry, Kentucky Cancer Registry (Lexington, KY, Tennessee Department of Health (Office of Cancer Surveillance), Florida Cancer Data System, North Carolina Central Cancer Registry, (North Carolina Division of Public Health), Georgia Comprehensive Cancer Registry, Louisiana Tumor Registry, Mississippi Cancer Registry, South Carolina Central Cancer Registry, Virginia Department of Health (Virginia Cancer Registry), Arkansas Department of Health (Cancer Registry). Data on SCCS cancer cases from Mississippi were collected by the Mississippi Cancer Registry, which participates in the National Program of Cancer Registries (NPCR) of the Centers for Disease Control and Prevention. The contents of this publication are solely the responsibility of the authors and do not necessarily represent the official views of the CDC or the Mississippi Cancer Registry.

The SEARCH study thanks the SEARCH and EPIC-Norfolk teams.

The TNBCC study thanks the Human Genetics Sample Bank for processing of samples and providing OSU Columbus area control samples.

Consortia Membership

Australian Ovarian Cancer Study Group

David D. Bowtell, Adele C. Green, Georgia Chenevix-Trench, Anna deFazio, Dorota Gertig, Penelope M. Webb.

The Breast and Ovarian Cancer Susceptibility Collaboration (BOCS)

A. Ardern-Jones, J. Adlard, M. Ahmed, G. Attard, K. Bailey, E. Bancroft, C. Bardsley, D. Barton, J. Barwell, L. Baxter, R. Belk, J. Berg, B. Bernhard, T. Bishop, L. Boyes, N. Bradshaw, A. F. Brady, S. Brant, C. Brewer, G. Brice, G. Bromilow, C. Brooks, A. Bruce, B. Bulman, L. Burgess, J. Campbell, N. Canham, B. Castle, R. Cetnarskyj, C. Chapman, O. Claber, N. Coates, T. Cole, A. Collins, J. Cook, S. Coulson, G. Crawford, D. Cruger, C. Cummings, L. D’Mello, R. Davidson, L. Day, L. de Silva, B. Dell, C. Dolling, A. Donaldson, H. Dorkins, F. Douglas, S. Downing, S. Drummond, C. Dubras, J. Dunlop, S. Durrell, D. Eccles, C. Eddy, M. Edwards, E. Edwards, J. Edwardson, R. Eeles, I. Ellis, F. Elmslie, G. Evans, B. Gibbens, C. Gardiner, N. Ghali, C. Giblin, S. Gibson, S. Goff, S. Goodman, D. Goudie, L. Greenhalgh, J. Greer, H. Gregory, D. Halliday, R. Hardy, C. Hartigan, T. Heaton, A. Henderson, C. Higgins, S. Hodgson, T. Holt, T. Homfray, D. Horrigan, C. Houghton, R. S. Houlston, L. Hughes, V. Hunt, L. Irvine, L. Izatt, C. Jacobs, S. James, M. James, L. Jeffers, I. Jobson, W. Jones, M.J. Kennedy, S. Kenwrick, C. Kightley, C. Kirk, L. Kirk, E. Kivuva, K. Kohut, M. Kosicka-Slawinska, A. Kulkarni, A. Kumar, F. Lalloo, N. Lambord, C. Langman, P. Leonard, S. Levene, S. Locker, P. Logan, M. Longmuir, A. Lucassen, V. Lyus, A. Magee, A. Male, S. Mansour, D. McBride, E. McCann, V. McConnell, M. McEntagart, C. McKeown, L. McLeish, D. McLeod, A. Melville, L. Mercer, C. Mercer, Z. Miedzybrodzka, A. Mitra, P. J. Morrison, V. Murday, A. Murray, K. Myhill, J. Myring, E. O’Hara, J. Paterson, P. Pearson, G. Pichert, K. Platt, M. Porteous, C. Pottinger, S. Price, L. Protheroe, S. Pugh, O. Quarrell, K. Randhawa, C. Riddick, L. Robertson, A. Robinson, V. Roffey-Johnson, M. Rogers, S. Rose, S. Rowe, A. Schofield, N. Rahman, S. Saya, G. Scott, J. Scott, A. Searle, S. Shanley, S. Sharif, A. Shaw, J. Shaw, J. Shea-Simonds, L. Side, J. Sillibourne, K. Simon, S. Simpson, S. Slater, S. Smalley, K. Smith, L. Snadden, K. Snape, J. Soloway, Y. Stait, B. Stayner, M. Steel, C. Steel, H. Stewart, D. Stirling, M. Thomas, S. Thomas, S. Tomkins, H. Turner, E. Tyler, A. Vandersteen, E. Wakeling, F. Waldrup, L. Walker, C. Watt, S. Watts, A. Webber, C. Whyte, J. Wiggins, E. Williams, L. Winchester.

GENICA Network

Dr. Margarete Fischer-Bosch-Institute of Clinical Pharmacology, Stuttgart, and University of Tübingen, Germany: HB, Wing-Yee Lo, Christina Justenhoven; German Cancer Consortium (DKTK) and German Cancer Research Center (DKFZ): HB: Department of Internal Medicine, Evangelische Kliniken Bonn gGmbH, Johanniter Krankenhaus, Bonn, Germany: YDK, Christian Baisch; Institute of Pathology, Medical Faculty University of Bonn, Germany: Hans-Peter Fischer; Molecular Genetics of Breast Cancer, Deutsches Krebsforschungszentrum (DKFZ), Heidelberg, Germany: UH; Institute for Prevention and Occupational Medicine of the German Social Accident Insurance, Institute of the Ruhr University Bochum (IPA), Bochum, Germany: TB, Beate Pesch, Sylvia Rabstein, Anne Lotz; Institute of Occupational Medicine and Maritime Medicine, University Medical Center Hamburg-Eppendorf, Germany: Volker Harth.

HEBON

Netherlands Cancer Institute, Amsterdam: Senno Verhoef, Martijn Verheus, Laura J. van’t Veer, Flora E. van Leeuwen; Erasmus Medical Center, Rotterdam: Margriet Collée, Maartje J. Hooning, Madeleine, M. A. Tilanus-Linthorst, Caroline Seynaeve; Leiden University Medical Center, Leiden: Christi J. van Asperen, Juul T. Wijnen, Rob A. Tollenaar; Radboud University Nijmegen Medical Center, Nijmegen: Marjolijn J. Ligtenberg; University Medical Center Utrecht, Utrecht: Margreet G. Ausems; Amsterdam Medical Center: Cora M. Aalfs, Theo A. van Os; VU University Medical Center, Amsterdam: Johan J. P. Gille, Quinten Waisfisz; University Hospital Maastricht, Maastricht: Cees E. van Roozendaal, Marinus J. Blok; University Medical Center Groningen University: Jan C. Oosterwijk, Annemarie H van der Hout, Marian J. Mourits; the Netherlands Foundation for the detection of hereditary tumours, Leiden: Hans F. Vasen.

kConFab*

David Amor, Lesley Andrews, Yoland Antill, Shane Armitage, Rosemary Balleine, Agnes Bankier, Patti Bastick, John Beilby, Barbara Bennett, Ian Bennett, Anneke Blackburn, Michael Bogwitz, Meagan Brennan, Melissa Brown, Michael Buckley, Matthew Burgess, Jo Burke, Phyllis Butow, Ian Campbell, Alice Christian, Georgia Chenevix-Trench, Christine Clarke, Alison Colley, Dick Cotton, Bronwyn Culling, Margaret Cummings, Sarah-Jane Dawson, Anna DeFazio, Martin Delatycki, Rebecca Dickson, Alexander Dobrovic, Tracy Dudding, Ted Edkins, Stacey Edwards, Gelareh Farshid, Susan Fawcett, Georgina Fenton, Michael Field, James Flanagan, Peter Fong, John Forbes, Stephen Fox, Juliet French, Clara Gaff, Mac Gardner, Mike Gattas, Graham Giles, Grantley Gill, Jack Goldblatt, Sian Greening, Scott Grist, Eric Haan, Marion Harris, Stewart Hart, Nick Hayward, Sue Healey, Louise Heiniger, John Hopper, Clare Hunt, Paul James, Mark Jenkins, Rick Kefford, Alexa Kidd, Belinda Kiely, Judy Kirk, James Kollias, Jessica Koehler, Serguei Kovalenko, Sunil Lakhani, Jennifer Leary, Geoff Lindeman, Lara Lipton, Liz Lobb, Graham Mann, Deborah Marsh, Bettina Meiser, Roger Milne, Gillian Mitchell, Shona O’Connell, Nick Pachter, Briony Patterson, Lester Peters, Kelly Phillips, Melanie Price, Lynne Purser, Tony Reeve, Edwina Rickard, Bridget Robinson, Barney Rudzki, Elizabeth Salisbury, Christobel Saunders, Joe Sambrook, Jodi Saunus, Robyn Sayer, Clare Scott, Elizabeth Scott, Rodney Scott, Adrienne Sexton, Raghwa Sharma, Andrew Shelling, Peter Simpson, Melissa Southey, Amanda Spurdle, Graeme Suthers, Pamela Sykes, Jessica Taylor, Ella Thompson, Heather Thorne, Sharron Townshend, Alison Trainer, Kathy Tucker, Janet Tyler, Jane Visvader, Logan Walker, Paul Waring, Robin Ward, Bev Warner, Rachael Williams, Ingrid Winship, Mary Ann Young. *Peter MacCallum Cancer Center, Melbourne, Australia.
