# Supplementary material for: Prediction of Breast Cancer Risk Based on Profiling With Common Genetic Variants
Source: J Natl Cancer Inst. 2015 Apr 2;107(5):djv036. doi: 10.1093/jnci/djv036 (PMC4754625; doi:10.1093/jnci/djv036)
Supplement: Supplementary Data [file supp_107_5_djv036__index.html]

Prediction of Breast Cancer Risk Based on Profiling With Common Genetic Variants — Supplementary Data 

# Prediction of Breast Cancer Risk Based on Profiling With Common Genetic Variants

## Supplementary Data

Data files

**Files in this Data Supplement:**

- Supplementary Data - Supplementary Data
