# Supplementary material for: Prediction of Breast Cancer Risk Based on Profiling With Common Genetic Variants
Source: J Natl Cancer Inst. 2015 Apr 2;107(5):djv036. doi: 10.1093/jnci/djv036 (PMC4754625; doi:10.1093/jnci/djv036)
Supplement: Supplementary Data [file supp_djv036_jnci_JNCI_14_0479_s01.docx]

# Supplementary Methods

## Study subjects and genotyping

Study participants for the primary analyses (set 1) were 89,049 women of European origin participating in 41 studies in the Breast Cancer Association Consortium (BCAC) (<http://www.srl.cam.ac.uk/consortia/bcac/index.html>, **Supplementary Tables 1-3**). The majority of studies were population or hospital-based case-control studies conducted in Australia, Europe, and North America.

Samples were genotyped using a custom Illumina iSelect array (iCOGS) comprising 211,155 SNPs designed in collaboration with three other consortia in COGS (1). For the analysis of SNP*SNP interactions, we included a further set of 72,014 women in BCAC genotyped for the relevant SNPs in earlier genotyping experiments (set 2) (2;3). For SNP*SNP analyses both familial and non-familial cases were included and tumours were invasive breast cancers, in-situ cancers or cancers of unknown invasiveness. For PRS analyses, studies that oversampled cases with a family history were excluded, since such studies yield biased estimates of the risks associated with genetic variants (4), and analyses were limited to invasive breast cancer. Ancestry was determined for women genotyped using the iCOGS array by computing identity by state followed by multidimensional scaling (1). Ethnicity was self-reported for other BCAC participants. Information on family history was primarily self-reported, but confirmation through cancer registration was possible for some studies. ER-status of the tumours was determined primarily from medical records, followed by centralised immunohistochemical analyses of whole sections and tissue microarrays as described previously (5).

Analyses were based primarily on variants reported to be associated at a genome-wide level of significance (taken as *P*<5x10-8) in the COGS experiment or a previous publication, either for breast cancer overall or for ER-negative breast cancer. We included two variants, rs1045485 in *CASP8* and rs2380205 on chromosome 10, that were reported to be associated with breast cancer, although the evidence for association with breast cancer is now weaker (1). We also included four additional variants identified through fine-mapping of the 5p12 (*TERT*) and 11q13 regions, that were associated with breast cancer at *P*<0·0001 in conditional analyses adjusting for the other known variants (6;7). For the SNP*SNP analysis, we included two additional variants (rs2981582 in FGFR2, rs614367 at 11q13) that were associated with risk and extensively genotyped in BCAC samples not genotyped using iCOGS, even though these were replaced by more strongly associated SNPs genotyped on iCOGS. The SNPs and regions included are summarised in **Supplementary Table 4**. Genotyping and quality control for the iCOGS chip (1) and previous genotyping phases in BCAC (2;8-12) have been described previously. All SNPs were genotyped directly, apart from rs78540526 and rs75915166 in *CCND1* that were imputed in the COGS dataset using IMPUTE2 v2 and the 1000 Genomes data ([1000 Genomes Phase I integrated variant set](https://webmail.medschl.cam.ac.uk/owa/redir.aspx?C=I62o1vuPrk6Xkt4VBaXqIKs2No3FVtAIYtfytMF6y0aVuMY5B_vyaRc0d4COFxHwXNJdLHU6jo8.&URL=http%3a%2f%2fmathgen.stats.ox.ac.uk%2fimpute%2fdata_download_1000G_phase1_integrated.html)) as a reference panel (13).

## Statistical Methods

### SNP*SNP interaction

Tests for pair-wise SNP*SNP multiplicative interactions were carried out using logistic regression, with breast cancer as the outcome. Each of the two SNPs was coded as a categorical variable (i.e. fitting a separate parameter for heterozygous and risk-allele homozygous genotypes), while the interaction terms (SNP1*SNP2) were included as continuous variables. All analyses were adjusted for study and seven principal components (PCs) to account for population sub-structure (1). Additional analyses were carried out with SNP main effects coded as continuous variables. As an alternative approach, we obtained parameter estimates for each study separately, and derived an overall test of interaction using a fixed effects meta-analysis.

To improve statistical power, case-only analyses were also conducted. The null hypothesis for case-only analyses is that there is no association between the two SNPs in cases. The test is only a valid test for multiplicative interaction if the two SNPs are not associated in the general population. This assumption was assessed in control-only analyses. Multinomial logistic regression was used for these analyses, with one SNP as the outcome and the other as the explanatory variable, adjusted by study and PCs, using the mlogit command in Stata. The parameter estimates were constrained such that the association was log-additive (i.e. such that the parameter estimate for homozygotes for the alternative allele of the outcome SNP was twice that of the heterozygotes, relative to the reference allele homozygotes), leading to a single interaction parameter and hence a 1df test of interaction.

We also carried out pairwise SNP*SNP analyses using both set 1 and set 2 (for a subset of SNPs) and combining the test of interaction results using a fixed-effects meta-analysis.

Probability-probability plots for observed vs expected log10p-values were constructed for the case-control analyses, and for case-only and control-only analyses after excluding SNP pairs strongly associated at p-value thresholds of p<0.0001 or p<0.01 in control-only analyses.

Since the number of tests was large (~3,000), interactions would need to have been significant p<1.7x10-5 to achieve an experiment-wise p<0.05 after correcting for multiple testing. Analyses were undertaken to determine the power of the tests for a representative subset of SNP pairs. Given the large sample size, the power to achieve this level of significance was, for most samples, good for an interaction effects size of 1.2, but poor for an effect size of 1.05. Example power calculations are shown in **Supplementary Table 11**. Power analyses were undertaken using the online program QUANTO (14;15).

### Association between PRS and breast cancer risk

To investigate the association between breast cancer risk and the combined effects of all 77 SNPs, a PRS was derived for each individual using the formula:

PRS = *β**x**β**x* *βkxk*+*βnxn*

Where *β*k is the per-allele log odds ratio (OR) for breast cancer associated with the minor allele for SNP *k*, and *xk* the number of alleles for the same SNP (0, 1 or 2), and n=77 is the total number of SNPs. Thus, the PRS was derived under the assumption that the ORs combine multiplicatively. Per-allele log OR estimates were obtained using logistic regression adjusting for study and seven principal components. For these estimations studies that oversampled cases with a family history were excluded. Log ORs were estimated individually for each SNP separately, except for sets of SNPs that were in the same region (*CCND1*, *ESR1*, *TERT*). For these SNP sets ORs were estimated from analyses in which all the SNPs were included in the regression. The same set of SNPs was used for to obtain PRSs separately for ER-positive and ER-negative disease, as from most SNPs small sample sizes precluded definitive identification of subtype-specific SNPs. However, SNP ORs estimated separately for each tumour subtype were used to derive the subtype-specific PRSs. The set of SNPs and the corresponding ORs used in the derivation of PRSs are shown in **Supplementary Table 4**.

Twenty-seven of the SNPs included in these analyses were previously reported in other studies, 24 of which included a subset of the samples in this report. The remaining SNPs were discovered through analyses based in part on the iCOGS dataset. To address potential for upward bias in effect size estimates, we performed a separate analysis using data from one large study (pKARMA) that was not part of any discovery analyses. For this analysis, we used only SNPs that reached genome-wide significance excluding pKARMA (**Supplementary Table 9**).

Logistic regression models were then used to estimate the odd ratios for breast cancer by percentile of the PRS. In these analyses, the middle quintile category (40-60th percentile) was used as the reference.

For some analyses the PRS was used as a continuous variable. In order to standardise the results and aid interpretation, we also calculated the logOR per unit standard deviation of the PRS. This was obtained by multiplying the effect size associated with the PRS in the logistic regression by the standard deviation of the normally distributed PRS in controls.

We evaluated modification of the PRS by age (defined as age at diagnosis for cases and age at interview for controls, and coded as a continuous variable, or categorised in four age groups: < 40, 40-49, 50-59 and >=60 years old), as well as modification by the presence or absence of family history of breast cancer in a first degree relative, by fitting additional interaction terms in the model. The OR for the interaction term was standardised by dividing by the standard deviation of the normally distributed PRS in controls.

All analyses were carried out using Stata v11. (StataCorp. 2009. *Stata Statistical Software: Release 11*. College Station, TX: StataCorp LP)**.**

### Comparison of the observed and predicted effect of the PRS on breast cancer risk under a multiplicative polygenic model of inheritance

Given a large number of unlinked loci each conferring a small effect, the PRS will have a distribution in the population (at birth) that is normal N(μ,σ), with mean β 

$$\mu=2\sum_{k} p_{k}\beta_{k}$$

and variance σ given by:

$$\sigma^{2}=\sum_{k} \sigma_{k}^{2}= 2\sum_{k} p_{k}q_{k}\beta_{k}^{2}$$

Where *pk* is the population minor allele frequency of the SNP k, *qk*=1-*pk*.

By definition under the logistic model (assuming a log-additive model with per-allele OR *βk*), the allele frequency in cases, *pk*′ is given by

$${p_{k}}^{'}=\frac{p_{k}e^{\beta_{k}}}{q_{k}+p_{k}e^{\beta_{k}}}$$

Where ${p^{'}}_{k}$is the allele frequency in cases.

Under a simple polygenic model, in which the risk of disease is due to a large number of alleles of small effect, combining multiplicatively, the distribution of the PRS in cases will also be normal, with mean:

$$\mu^{'}=2\sum_{k} {p^{'}}_{k}\beta_{k}=2\sum_{k} \left( \frac{p_{k}e^{\beta_{k}}}{q_{k}+p_{k}e^{\beta_{k}}} \right)\beta_{k}=2\sum_{k} p_{k}\beta_{k}+2\sum_{k} \left( \frac{p_{k}q_{k}\left( e^{\beta_{k}}-1 \right)}{q_{k}+p_{k}e^{\beta_{k}}} \right)\beta_{k}\approx\mu+ 2\sum_{k} p_{k}q_{k}\beta_{k}^{2} \approx\mu+ \sigma^{2}$$

and variance:

$$\sigma^{'2}= \sum_{k} {\sigma_{k}}^{2}=2\sum_{k} {p'}_{k}{q'}_{k}\beta_{k}^{2}\approx\sigma^{2}$$

The difference between the minor allele frequency in cases and in controls is:

$${p_{k}}^{'}-p_{k}=\frac{p_{k}{q_{k}(e}^{\beta_{k}}-1)}{q_{k}+p_{k}e^{\beta_{k}}}$$

Since ${p^{'}}_{k} \approx p_{k}$ and ${q^{'}}_{k} \approx q_{k}$ the approximation is valid when $\beta_{k}\approx0$.

Thus the PRS has approximately the same distribution in cases as controls but is shifted to the right by the variance σ2 (16).

Analogous formulae based on haplotypes were used for linked sets of SNPs using effect sizes and minor allele frequencies derived using the computer program *haplo.stats* (<http://mayoresearch.mayo.edu/mayo/research/biostat/schaid.cfm>).

This approximation can be used to derive theoretical predictions for the OR for individuals with PRS above or below a given threshold (or between two percentiles), vs the 40-60th percentile. Thus, the OR for individuals in the top η of the distribution, compared to individuals in the middle quintile (40-60th percentile) α, is given by:

$$\text{ }\frac{\alpha'\eta}{\eta'\alpha}=\frac{\alpha(1-\Phi(\Phi^{-1}(1-\eta)+\sigma))}{(1-\eta)\Phi(\Phi^{-1}(\alpha)+\sigma)}$$

Where$\text{ }\Phi$ is the cumulative distribution of a standard normal curve.

Note that the observed and predicted risks were calculated relative to the middle quintile (40-60th percentile) and not the population mean. To calculate risks relative to the population mean, these risks were divided by the weighted sum of the above odds ratios, OR

Ψ = Pj ORj


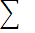

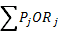


where $P_{j}$ Pj is the proportion of the population in bin j. For this SNP set Ψ was estimated to be 1.08.

**Confidence limits for theoretical polygenic risk score**

The confidence limits on the predicted risks depend on the variance of σ2. For 2 allele markers σ2­­ is given by:

$${var(\sigma}^{2})=4\sum_{k} p_{k}^{2}{q_{k}^{2}var(\beta}_{k}^{2})$$

Where ${var(\beta}_{k}^{2})$ is given by:

$${var(\beta}_{k}^{2})=2\tau_{k}^{4}+4{\beta_{k}^{2}\tau}_{k}^{2}$$

Where $\tau_{k}$ is the standard error of $\beta_{k}$

Providing $\tau_{k}\ll\beta_{k}$ (which will be true for highly significant SNPs) this approximates to

$${var(\beta}_{k}^{2})\approx4{\beta_{k}^{2}\tau}_{k}^{2}$$

For markers with more than two alleles (or haplotypes), the corresponding formula for σ2 ­is

$$\sigma^{2}=2\sum_{k} \sigma_{k}^{2}= 2\sum_{k} \left[ \sum_{j} p_{kj}\beta_{kj}^{2}-\left( \sum_{j} p_{kj}\beta_{kj} \right)^{2} \right]$$

Where now $\beta_{kj}$ is the log-relative risk for the jth allele of marker k, with baseline allele 0 having $\beta_{k0}=0$ and *pkj*­  is the corresponding frequency.

The variance of σ2 is then given by:

${var(\sigma}^{2})=4var(\sigma_{k}^{2}$)

Where

$$var(\sigma_{k}^{2})\approx4\sum_{j,l} \acute{\delta_{kj}}S_{kjl}\acute{\delta}_{kl}$$

Where *S*kjl­ is the covariance between the parameter estimates $\beta_{kj}$ and $\beta_{kl}$ for marker/haplotype k, and

$\acute{\delta}_{kj}=p_{kj}{(\beta}_{kj}- \sum_{j} p_{kj}\beta_{kj}$)

### Absolute risks of developing breast cancer by percentiles of the PRS

The absolute risk of breast cancer for individuals in each risk category was calculated taking into account the competing risk of dying from other causes apart from breast cancer. According to standard theory the overall incidence of breast cancer at age t, i(t) is equal to the density function divided by the survival function. These in turn expressed in terms of the survival probabilities for each PRS category:

Where n is the total number of risk categories, is the frequency of PRS category *g*,$\mu_{g}(t)$is the breast cancer incidence associated with PRS category g and $S_{g}(t-1)$ is the survival function associated with category *g*. The breast cancer incidence$\mu_{g}(t)$for each PRS category is derived from the baseline incidence $\mu_{o}(t)$and the corresponding effect sizeusing$\mu_{g}(t)=\mu_{0}(t)\exp(\beta_{g})$, so that:

Since the overall incidence rates are known, the baseline incidence rates can be derived iteratively starting from age 0 (17). For this study, we used 2010 breast cancer incidence rates for UK (18). Interaction between age and the PRS was accounted for in the model by including the interaction term (PRS*age) for each PRS category. The absolute risk (ARg(t)) of breast cancer in category *g*, taking into account the competing risk of dying from causes other than breast cancer was calculated by:

Where $S_{g}(t)$is the probability of being free of breast cancer to age *t* and $S_{m}(t)$the probability of surviving to age *t*, i.e. not dying from a cause other than from breast cancer. Approximate confidence limits for the absolute risk were derived from the variance-covariance matrix of the parameter estimates, under the constraint that the overall incidence rates of breast cancer should agree the population rates as noted above, by iterative application of the delta method.

The absolute risk of developing subtype specific disease was also calculated, constraining to the incidence of overall incidence of ER-negative and ER-positive disease in the UK (derived from the overall incidence of breast cancer from the UK population data, and the age-specific proportions of ER-negative and ER-positive tumours). Women are at risk of developing both ER-negative and ER-positive disease, therefore the absolute risks were calculated given that the individual has been free of breast cancer of any subtype. For example twenty five risk categories can be specified, according to quintiles of the risk score for ER-negative and quintiles of the risk score for ER-positive disease.

The probability of a woman developing breast cancer by any age t2, given she is alive and free of breast cancer at age t1, was calculated as: (AR(t2)-AR(t1))/(*S*g(t1)**S*m(t1))

The 2.4% threshold was calculated as the average 10-year absolute risk for a 47 year old woman in the UK based on 2006-2010 incidence and mortality rates (Office of National Statistics, <http://www.ons.gov.uk/>) (The age at which routine mammographic screening starts is currently 50 years, but this is being reduced to 47 years).

Mortality rates from all causes were derived from data on the number of individuals died in each age group and the total population in each age group (population census) in 2011 from the Office for National Statistics (http://www.ons.gov.uk/). Data on mortality from breast cancer was obtained from the CRUK website (<http://www.cancerresearchuk.org>). Estimates of the age-specific proportions of breast cancer by tumour subtype in the UK population were obtained from the West Midlands Cancer Intelligence Unit (http://www.wmciu.nhs.uk/).

# Supplementary Figures

## Supplementary Figure 1. Case-control analyses testing for pair-wise interaction between SNPS known to be associated with breast cancer at genome-wide significance levels

P-P plots show observed vs expected log10 p-values for (A) all breast cancers, (B) ER-positive disease, and (C) ER-negative disease.

**(A)**

**
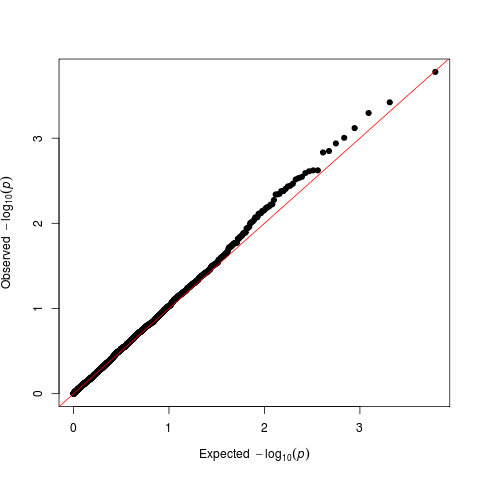
**

**(B)**

**
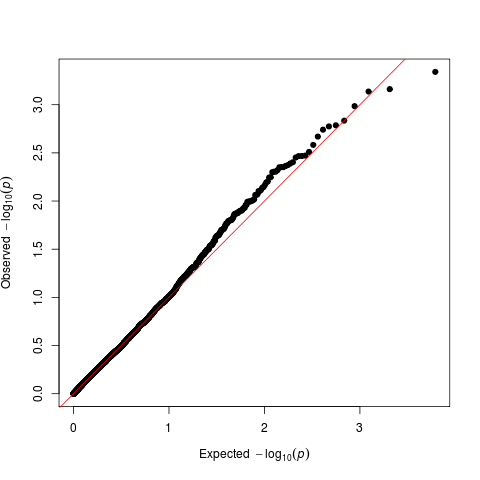
**

**(C)**

**
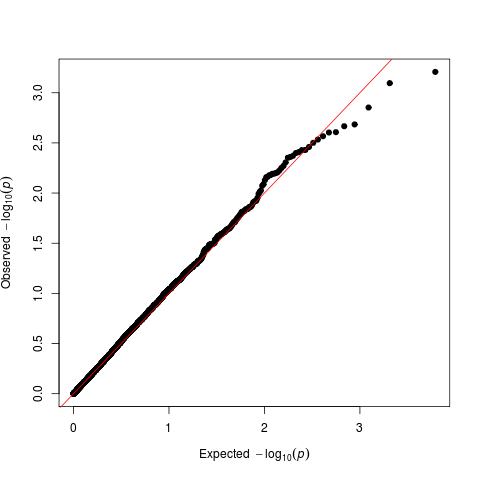
**

Supplementary Figure 2. Association between the Polygenic Risk Score (PRS) and breast cancer risk in different age groups.ORs are for different deciles of the PRS relative to the 50-59 percentile. Women were categorized by age at diagnosis for cases and age at interview for controls.

## Supplementary Table 1. Participating studies in SNP*SNP interaction analysis and PRS analysis

| **Study Acronym** | | **Study Name**  **(Reference)** | **Country** | **Recruitment base** | | |
| --- | --- | --- | --- | --- | --- | --- |
|  |  |  |  | **Cases** | **Controls** | |
| ABCFS | | Australian Breast Cancer Family Study (19) | Australia | Cancer registries in Victoria and New South Wales (1992-1999): all cases from Melbourne and Sydney diagnosed before age 40 plus a random sample of those diagnosed at ages 40-59. | | Identified between 1992 and 1999 from the electoral rolls in Melbourne and Sydney (enrolling to vote is compulsory); frequency matched to cases by age in-5 year categories. |
| ABCS | | Amsterdam Breast Cancer Study (20) | Netherlands | Breast cancer patients diagnosed before age 50 in 2003-2009 at the NKI-AVL; and (ABCS-F) All non-BRCA1/2 breast cancer cases from the family cancer clinic of the NKI-AVL tested in the period 1995-2009; all ages and diagnosed with breast cancer in 1965-2008. | | Population-based cohort of women recruited through the Sanquin blood bank, all ages. |
| ABCTB | | Australian Breast Cancer Tissue Bank | Australia | Newly diagnosed unselected cases from 32 hospitals in New South Wales from 2006 | | No controls |
| BBCC | | Bavarian Breast Cancer Cases and Controls (21) | Germany | Consecutive, unselected cases with invasive breast cancer recruited at the University Breast Centre, Franconia in Northern Bavaria from 2002-2010. | | Healthy women aged 55 or older with no diagnosis of cancer. Invited by a newspaper advertisement in Northern Bavaria between 2002 and 2010. |
| BBCS | | British Breast Cancer Study (22) | U.K. | (i) English & Scottish Cancer Registries: all breast cancer cases who developed a first primary before age 66 in 1971 or later and who subsequently developed a second primary cancer. (ii) Breast Cancer Clinics: all breast cancer cases who developed a first primary before age 71 in 1967 or later and who either subsequently developed a second primary or had at least two affected female first-degree relatives.  All recruited from 2001-2008. | | A friend, sister-in-law, daughter-in-law or other non-blood relative of cases, recruited from 2001-2008. |
| BIGGS | | Breast Cancer in Galway Genetic Study (23) | Ireland | Unselected cases recruited from University College Hospital Galway and surrounding hospitals in the West of Ireland since 2001 | | Women > 60 years with no personal history of any cancer and no family history of breast or ovarian cancer identified from retirement groups in the West of Ireland between 2001 and 2008. |
| BSUCH | | Breast Cancer Study of the University Clinic Heidelberg (24) | Germany | All cases diagnosed with breast cancer in 2007-2009 at the University Women`s Clinic Heidelberg. | | Female blood donors recruited in 2007- 2009 at the Institute of Transfusion Medicine & Immunology, Mannheim. |
| CECILE | | CECILE Breast cancer study (25) | France | All cases diagnosed with breast cancer in 2005-2007 among women <75 years of age residing in the *départements* of Ille-et-Vilaine and Côte d’Or. Cases were recruited from the main cancer treatment centre (Centre Eugène-Marquis in Rennes and Centre Georges-François-Leclerc in Dijon) and from other private or public hospitals in each area. | | General population control women residing in the same areas as the cases (Ille-et-Vilaine and Côte d’Or). Controls were frequency-matched to the cases by 5-year age groups. They were recruited in 2005-2007 using a random digit dialling procedure and quotas by socioeconomic status to reflect the distribution by SES of the population in each area. |
| CGPS | | Copenhagen General Population Study (26) | Denmark | Consecutive, incident cases from one hospital with centralized care for a population of 400,000 women in Copenhagen (2001-present). | | Women with no history of breast cancer residing in the same region as cases identified from the Copenhagen General Population Study (2003-2007). |
| CNIO-BCS | | Spanish National Cancer Centre Breast Cancer Study (27) | Spain | (i) consecutive breast cancer patients from three public hospitals, two in Madrid and one in Oviedo;  (ii) cases with at least one affected first degree relative recruited through the CNIO family cancer clinic in Madrid (2000-2005). | | Women attending the Menopause Research Centre, Madrid and female members of the College of Lawyers attending a free, targeted medical check-up in Madrid, all free of breast cancer and all in Madrid between 2000-2005. |
| CTS* | | California Teachers  Study (28) | USA | Nested case-control study conducted within a cohort of California educators (133,479) who were under age 80 years at baseline, had no prior history of invasive or *in situ* breast cancer. Cases are women newly diagnosed with a histologically confirmed invasive primary adenocarcinoma of the breast at age 80 years or younger from 1998 to 2008. | | Controls are a probability sample of at-risk cohort members, frequency matched to cases on age at baseline (5-year age groups), self-reported race/ethnicity (white, African American, Latina, Asian, other), and broad geographic region within California.  Controls were selected without replacement, using an assigned reference date. |
| DBCSS | | DietCompLyf Breast Cancer Survival Study | UK | Invasive primary breast cancer grade I-III, patients recruited 9 - 15 months after diagnosis, <age 75. Recruitment throughout UK. Patient first recruited on 18/2/97. Study joined NCRN in July 2004. Recruitment finished on 31/8/10 | | No controls |
| DEMOKRITOS* | | DEMOKRITOS (29) | Greece | Triple negative breast cancer cases enrolled from 1997-2010 in hospitals serving geographical areas of Greece, including Athens metropolitan area, Thessaloniki, Ioannina, Patras, and Crete (Chania), in collaboration with the Hellenic Cooperative Oncology Group (HECOG). | | Regional controls from Athens, Greece were population-based unaffected women of the same age range. |
|  | ESTHER | ESTHER Breast Cancer Study (30) | Germany | Breast cancer cases in all hospitals in the state of Saarland, from 2001-2003 (ESTHER) and 1996-1998 (VERDI). | | Random sample of women participating in a routine health check-up in Saarland, in 2000-2002; frequency matched to cases by age in-5 year categories. |
|  | FBCS | ICR Familial Breast Cancer Study (28;31) | UK | Index patients with breast cancer and at least 2 relatives affected with breast cancer. All White individuals collected in 1995-2005 throughout UK | | White individuals collected throughout UK from 1958 Birth Cohort Collection, an ongoing follow-up of persons born in UK in one week in 1958 |
|  | GC-HBOC | German Consortium for Hereditary Breast & Ovarian Cancer (32-34) | Germany | Index patients from German breast cancer families; BRCA1/2 mutation free, collected 1996-2007 via Institute of Human Genetics, University Heidelberg & Department of Gynaecology & Obstetrics, Cologne & Department of Gynaecology and Obstetrics at the Ludwig-Maximilians-University, Munich; Germany. | | Healthy, unrelated, ethnically matched female blood donors recruited in 2004 & 2007 by German Red Cross Blood Service of Baden-Württemberg-Hessen, Institute of Transfusion Medicine & Immunology, Mannheim. |
|  | GENICA | Gene Environment Interaction & Breast Cancer in Germany (35;36) | Germany | Incident breast cancer cases enrolled at hospitals  in the Greater Bonn area between 2000-2004. | | Random address sample selected in 2001-2004 from 31 population registries in the greater Bonn area; frequency matched to cases on year of birth in 5-year categories. |
|  | GESBC | Genetic Epidemiology Study of Breast Cancer by Age 50 (37) | Germany | All incident cases diagnosed <50 years of age in 1992-5 in two regions: Rhein-Neckar-Odenwald and Freiburg, by surveying the 38 clinics serving these regions. | | Selected from random lists of residents of the study regions supplied by population registries; two controls were selected for each case, matched by age and study region. Recruitment was carried out 1992-1998. |
|  | HABCS | Hannover Breast Cancer Study (38) | Germany | Cases who received radiotherapy for breast cancer at Hannover Medical School between 1997-2003, unselected for age or family history | | Anonymous female blood bank donors at Hannover Medical School, collected from 8/2005-12/2005, with known age and ethnic background |
|  | HEBCS | Helsinki Breast Cancer Study (39;40) | Finland | (1) Consecutive cases (883) from the Department of Oncology, Helsinki University Central Hospital 1997-8 and 2000, (2) Consecutive cases (986) from the Department of Surgery, Helsinki University Central Hospital 2001 – 2004, (3) Familial breast cancer patients (536) from the Helsinki University Central Hospital, Departments of Oncology and Clinical Genetics (1995-). | | Healthy females from the same geographical region in Southern Finland in 2003. |
|  | HMBCS | Hannover-Minsk Breast Cancer Study (41) | Belarus | Cases from the Byelorussian Institute for Oncology and Medical Radiology Aleksandrov N.N. in Minsk or at one of 5 regional oncology centres in Gomel, Mogilev, Grodno, Brest or Vitebsk (2002-2008). | | Women attending general medical examination at gynecology clinics in Gomel, Mogilev, Grodno, Brest or Vitebsk; women attending the Institute for Inherited Diseases in Minsk; female blood donors in Minsk (2002-2008). |
|  | HUBCS | Hannover-Ufa Breast Cancer Study (42) | Russia | Consecutive Russian breast cancer patients aged 24-86 years ascertained at one of the two participating oncological centers in Bashkorstostan and Siberia through the years 2000-2008 | | Population controls aged 18-84 years recruited from a population study of different populations of Russia. Healthy volunteers (without any malignancy) were selected from the same geographical regions during the years 2002-2008. |
|  | KARBAC | Karolinska Breast Cancer Study (43;44) | Sweden | 1. Familial cases from Department of Clinical Genetics, Karolinska University Hospital, Stockholm. 2. Consecutive cases from Department of Oncology, Huddinge & Söder Hospital, Stockholm 1998-2000. | | Blood donors of mixed gender from same geographical region. Excess material was received from all blood donors over a 3 month period in 2004 (approximately 3000) and DNA was extracted from a random sample of 1500. |
|  | KBCP | Kuopio Breast Cancer Project (45) | Finland | Women seen at Kuopio University Hospital between 1990-1995 because of a breast lump, mammographic abnormality, or other breast symptom and who were found to have breast cancer. | | Selected from the National Population Register between 1990 and 1995; age and long-term area-of-residence matched to cases. |
|  | kConFab/  AOCS | Kathleen Cuningham Foundation Consortium for Research into Familial Breast Cancer / Australian Ovarian Cancer Study (46) | Australia | Index (youngest affected) cases from *BRCA1*- and *BRCA2*-mutation-negative multiple-case breast and breast-ovarian families recruited though family cancer clinics from across Australia and New Zealand from 1998-present. | | Identified from the electoral rolls from across Australia as part of the Australian Ovarian Cancer Study in 2002-2006. |
|  | LMBC | Leuven Multidisciplinary Breast Centre (47) | Belgium | All patients diagnosed with breast cancer and seen in the Multidisciplinary Breast Centre in Leuven (Gashuisberg) since June 2007 plus retrospective collection of cases diagnosed since 2000. | | Blood donors at Gasthuisberg Hospital (200-2008). |
|  | MARIE | Mammary Carcinoma Risk Factor Investigation (48) | Germany | Incident cases diagnosed from 2001-2005 in the study region Hamburg in Northern Germany, and from 2002-2005 in the study region Rhein-Neckar-Karlsruhe in Southern Germany. | | 2 controls per case were randomly drawn from population registries and frequency matched by birth year and study region to the case. Controls were recruited from 2002 to 2006. |
|  | MBCSG | Milan Breast Cancer Study Group (49) | Italy | Familial and/or early onset breast cancer patients (aged 22-87) negative for mutations in *BRCA1* and *BRCA2*, ascertained at two large cancer centres in Milan from 2000-present. | | Female blood donors recruited at two centres in Milan from 2004-present and 2007-present. |
|  | MCBCS | Mayo Clinic Breast Cancer Study (50) | U.S.A. | Incident cases residing in 6 states (MN, WI, IA, IL, ND, SD) seen at the Mayo Clinic in Rochester, MN from 2002-2010. | | Women presenting for general medical examination at the Mayo Clinic from 2002-2010; frequency matched to cases on age, ethnicity and county/state. |
|  | MCCS | Melbourne Collaborative Cohort Study (51) | Australia | Incident cases from the cohort of 24,469 women, diagnosed during the follow-up from baseline (1990-1994) to 2008. | | Random sample of the initial cohort. |
|  | MEC | Multiethnic Cohort (52) | USA | Incident cases identified from SEER cancer registries in Los Angeles County & State registries in California & Hawaii, USA from 1993-2002. Grouped by self-reported ethnicity. | | Women without cancer from the same States, recruited concurrently with cases & frequency matched to cases by age at blood-draw & self-reported ethnicity. |
|  | MSKCC | Memorial Sloan-Kettering Cancer Center Study (53) | USA | Incident and prevalent cases of histologically-confirmed breast cancer referred to the Clinical Genetics Service at MSKCC since July 1996. All cases tested negative for BRCA1/2 mutations. | | Women who have not been diagnosed with breast cancer, but who were referred to the Clinical Genetics Service at MSKCC. All controls tested negative for BRCA1/2 mutations. |
|  | MTLGEBCS | Montreal Gene-Environment Breast Cancer Study | Canada | All cases are postmenopausal women (47-75 years) living in Montreal with a primary invasive breast cancer and with no previous occurrence of any type of cancer. All cases were identified from 2007 to 2010 in 15 of 16 Montreal hospitals that treat breast cancer. | | Random sample from the universal Provincial Voter Registration List, approximately frequency-matched to cases on age (5-year bins) and living in Montreal. |
|  | NBCS* | Norwegian Breast Cancer Study (54) | Norway | Incidence cases from three different hospitals: Ullevål Univ. Hospital 1990-94, Norwegian Radium Hospital 1975-1986 and 1995-1998, Haukeland Univ. Hospital 1992-2001. | | Women residing in Tromsø and Bergen who attended the Norwegian Breast Cancer Screening Program. |
|  | NBHS_TN* | Nashville Breast Health Study (Triple Negative) | U.S.A. | Triple negative invasive breast cancer cases from a collection of Invasive breast cancer or ductal carcinoma in situ cases between the ages of 25 and 75 years (2001-2010). Cases were identified from participating hospitals in the Nashville Metropolitan area and the Tennessee Cancer Registry (TCR). | | Controls were recruited through random digit dialing. |
|  | NC-BCFR | Northern California Breast Cancer Family Registry (55) | USA | Cases included those enrolled in the NC-BCFR as part of Phase I and II recruitment. Incident cases aged <65 years diagnosed between 1995 and 2003 were identified through the SEER cancer registry of the Greater San Francisco Bay Area. All cases likely at increased genetic risk were eligible to enroll in the BCFR (dx at age <35 yrs, personal history of ovarian or childhood cancer, bilateral breast cancer with 1st dx at age <50, family history of breast or ovarian cancer in first-degree relatives). Cases not meeting these criteria were randomly sampled (2.5% of whites, 30% of African Americans, 28% of Hispanics, 38% of Asian Americans). | | Controls were identified through random digit dialing conducted from 1999-2000 in the same geographic region. Controls were frequency matched to cases on 5-year age group and race/ethnicity, at a ratio of 1 control per 2 cases. |
|  | NHS | Nurses Health Study (56) | USA | Incident cases arising in the sub-cohort of 32,826 cohort members who gave a blood specimen in 1989-1990 are included if they were diagnosed with breast cancer prior to July 1, 2000. | | Controls were women in this sub-cohort who were not diagnosed with breast cancer. Controls were matched to cases on age, postmenopausal status and postmenopausal hormone use. |
|  | OBCS | Oulu Breast Cancer Study (57) | Finland | Consecutive incident cases diagnosed at the Oulu University Hospital between 2000 and 2004. | | Female blood donors recruited in 2002 from the same geographical region in Northern Finland. |
|  | OFBCR | Ontario Familial Breast Cancer Registry55 | Canada | Invasive cases aged 20-54 years identified from the Ontario Cancer Registry from 1996-1998. All those at high genetic risk were eligible; random samples of women not meeting these criteria were also asked to participate. During 2001-2005, enrolment was limited to minority and high-risk families. | | Identified by calling randomly selected residential telephone numbers in the same geographical region from 1998-2001; frequency matched to cases by age in 5 year categories. |
|  | ORIGO | Leiden University Medical Centre Breast Cancer Study (58;59) | Netherlands | Consecutive case patients diagnosed 1996–2006 in 2 hospitals in South–West Netherlands (Leiden & Rotterdam). No selection for family history; Rotterdam case patients selected for diagnosis aged <70. Case patients with in situ carcinomas eligible. | | (1) Blood bank healthy donors from Southwest Netherlands recruited in 1996, 2000 or 2007; (2) People who married a person who was part of a family with high breast cancer risk (BRCA1/2/x). From the Southwest of the Netherlands, recruited 1990–1996; (3) Females tested at the local clinical genetics department for familial diseases, excluding familial cancer syndromes (no mutation found in gene(s) related to the disease being tested), recruited 1995–2007. |
|  | OSU* | The Stefanie Spielman Breast Bank and the Columbus Area Control Sample Bank | U.S.A. | Incident triple negative breast cancer cases enrolled at The Ohio State University James Comprehensive Cancer Centre between 2003 and 2011. | | Controls were chosen from an existing bank of individuals seen for routine health issues at primary care and internal medicine clinics, recruited 2007-2011. They were age and ethnicity frequency matched to cases. |
|  | PBCS | NCI Polish Breast Cancer Study (60) | Poland | Incident cases identified through a rapid identification system in participating hospitals covering ~ 90% of all eligible cases, and cancer registries in Warsaw and Łódź covering 100% of all eligible cases (2000-2003). | | Randomly selected from population lists of all residents of Poland from 2000-2003, stratified and frequency matched to cases on city and age in 5-year categories. |
|  | pKARMA | Karolinska Mammography Project for Risk Prediction of Breast Cancer - prevalent cases | Sweden | Incident cases from Jan 2001 – Dec 2008 from the Stockholm/Gotland area. Identified through the Stockholm breast cancer registry. | | Unmatched participants of the KARMA mammography screening study recruited between 2010 and 2011 from Helsingborg and Stockholm. |
|  | POSH | Prospective Study of Outcomes in Sporadic Versus Hereditary Breast Cancer (61;62) | UK | Cases aged 40 or younger at breast cancer diagnosis. Recruited across UK and diagnosed between January 2000 to December 2007 | | No in-house controls |
|  | RBCS | Rotterdam Breast Cancer Study (2) | Netherlands | Familial breast cancer patients selected from the clinical genetics centre at Erasmus Medical Centre between 1994 and 2005. | | Spouses or mutation-negative siblings of heterozygous Cystic Fibrosis mutation carriers selected from the clinical genetics centre at Erasmus Medical Centre between 1996 and 2006. |
|  | RPCI* | Roswell Park Cancer Institute | U.S.A | Triple negative invasive breast cancer cases from incident cases recruited to the RPCI Data Bank and Biorepository. | | Healthy controls identified from employee volunteers, and women recruited from community events. |
|  | SASBAC | Singapore and Sweden Breast Cancer Study(63) | Sweden | Women diagnosed in Sweden aged 50-74 in 1993-1995. | | Population-based controls frequency matched by age to the cases. |
|  | SBCS | Sheffield Breast Cancer Study (64) | U.K. | Women with breast cancer recruited in 1998-2005 at surgical outpatient clinics at the Royal Hallamshire Hospital, Sheffield. | | Unselected women attending the Sheffield Mammography Screening Service in 2000-2004 with no evidence of a breast lesion. |
|  | SEARCH | Study of Epidemiology & Risk Factors in Cancer Heredity (65) | U.K. | Identified through the Eastern Cancer Registration and Information Centre: (i) prevalent cases; diagnosed 1991-1996; under 55 years of age at diagnosis; recruited 1996-2002 (ii) incident cases; diagnosed since 1996; under 70 years of age at diagnosis; recruited 1996-present. | | (a) Women from the same geographic region selected from the EPIC-Norfolk cohort study, 1992-1994 (b) women attending GP practices, frequency matched to cases by age and geographic region (2003-2010). |
|  | SKKDKFZS* | Städtisches Klinikum Karlsruhe Deutsches Krebsforschungszentrum Study (66) | Germany | Women diagnosed with primary *in situ* or invasive breast cancer at the Städtisches Klinikum Karlsruhe from March 1993 to July 2005. Cases were 21-93 years of age. | | Controls selected from GC-HBOC |
|  | SZBCS | IHCC-Szczecin Breast Cancer Study (67) | Poland | Prospectively ascertained cases of invasive breast cancer patients diagnosed at the Regional Oncology Hospital (2002-2003 and 2006-2007) or the University Hospital (2002-2007), both in Szczecin, West Pomerania, Poland. | | Selected from a population-based study of the 1.3 million inhabitants of West Pomerania (2003-2004); matched to cases for year of birth, sex and region. |
|  | UKBGS | Breakthrough Generations Study (68) | UK | Cohort members who developed breast cancer or in situ breast cancer after entry into the Breakthrough Generations Study (cohort of >100,000 women followed up for breast cancer, recruited from the UK during 2003 -2010). | | Women who had not had breast cancer or in situ breast cancer selected by 1:1 matching to cases on date of birth, year of entry into the study (2003-2010), source of recruitment, availability of blood sample and ethnicity. |
|  | UCIBCS | UCI Breast Cancer Study (69;70) | USA | All cases diagnosed in Orange County, California, during one-year period beginning March 1, 1994. Ascertained through the population-based Cancer Surveillance Program of Orange County California (CSPOC) | | Female controls under age 75 years without history of cancer recruited using random digit dialing among Orange County residents & frequency matched to cases by age & race/ethnicity. Recruited from 1998-2003. |
|  | US3SS | US Three State Study (71) | USA | Eligible cases were all English-speaking female residents of Massachusetts (excluding metropolitan Boston), New Hampshire and Wisconsin, with a new diagnosis of invasive (aged 20–69 years) or in situ breast cancer (aged 20–74 years, MA and NH only) reported to each state's mandatory cancer registry during 1998-2001. | | Controls were randomly selected from driver’s license lists for women aged 20–64 years and from Medicare beneficiary lists for women aged 65–74 years in each state, and frequency-matched to cases by age in 5-year categories. Recruited 1998-2001. |
|  | USRT | US Radiologic Technologists Study  (72-75) | USA | Prevalent cases identified through mailed surveys in 1983-8 and 1994-8, incident cases between surveys; blood collected from 1999-2004; unselected for family cancer history or any other characteristics; most cases sampled more than 5 years after diagnosis. | | Selected from women within the cohort without breast cancer as of 1999, blood collected between 2000-2004; matched to cases on year of birth in 5-year strata. |

* CTS, NBCS and SKKDKFZS are studies in BCAC but genotyped as part of the triple negative consortium (TNBCC). Part of GENICA was also genotyped as part of TNBCC. DEMOKRITOS, NBHS_TN, OSU and RPCI were additional non-BCAC studies included as part of the TNBCC.

## Supplementary Table 2. Number of individuals and studies used for SNP*SNP interaction and PRS analyses

| **SNP*SNP interaction analysis** | | | | | | | | |  | **PRS analysis** | | | | | | | |  | |
| --- | --- | --- | --- | --- | --- | --- | --- | --- | --- | --- | --- | --- | --- | --- | --- | --- | --- | --- | --- |
| **iCOGS (set 1)** | **Cases** | **Controls** | **Total** |  | **BCAC**† **(set 2)** | **Cases** | **Controls** | **Total** |  | **iCOGS** | | **Cases** | | **Controls** | | | **Total** |  | |
| ABCFS | 790 | 551 | 1,341 |  | ABCFS | 504 | 166 | 670 |  | ABCFS | | 689 | | 490 | | | 1,179 | |
| ABCS | 1,325 | 1,429 | 2,754 |  | ABCS | 1,571 | 996 | 2,567 |  | ABCS | | 675 | | 1,400 | | | 2,075 | |
| BBCC | 564 | 458 | 1,022 |  | ABCTB | 1,205 | 0 | 1,205 |  | BBCC | | 546 | | 456 | | | 1,002 | |
| BBCS | 1,554 | 1,397 | 2,951 |  | BBCC | 1,423 | 1,015 | 2,438 |  | BIGGS | | 765 | | 704 | | | 1,469 | |
| BIGGS | 836 | 719 | 1,555 |  | BBCS | 1,372 | 1,046 | 2,418 |  | BSUCH | | 782 | | 934 | | | 1,716 | |
| BSUCH | 852 | 954 | 1,806 |  | BIGGS | 129 | 88 | 217 |  | CECILE | | 862 | | 956 | | | 1,818 | |
| CECILE | 1,019 | 999 | 2,018 |  | BSUCH | 314 | 872 | 1,186 |  | CGPS | | 2,682 | | 3,991 | | | 6,673 | |
| CGPS | 2,901 | 4,086 | 6,987 |  | CECILE | 36 | 36 | 72 |  | CTS | | 68 | | 70 | | | 138 | |
| CNIO-BCS | 902 | 876 | 1,778 |  | CGPS | 429 | 6,705 | 7,134 |  | DEMOKRITOS | | 407 | | 93 | | | 500 | |
| CTS | 68 | 70 | 138 |  | CNIO-BCS | 383 | 491 | 874 |  | ESTHER | | 461 | | 496 | | | 957 | |
| DEMOKRITOS | 413 | 95 | 508 |  | CTS | 1,208 | 1,190 | 2,398 |  | GENICA | | 432 | | 357 | | | 789 | |
| ESTHER | 478 | 502 | 980 |  | DBCSS | 949 | 0 | 949 |  | HMBCS | | 672 | | 110 | | | 782 | |
| GENICA | 465 | 427 | 892 |  | ESTHER | 20 | 2 | 22 |  | KBCP | | 389 | | 223 | | | 612 | |
| HEBCS | 1,664 | 1,234 | 2,898 |  | FBCS | 1,832 | 1,056 | 2,888 |  | LMBC | | 2,528 | | 1,342 | | | 3,870 | |
| HMBCS | 690 | 130 | 820 |  | GC-HBOC | 1,030 | 1,335 | 2,365 |  | MARIE | | 1,404 | | 1,553 | | | 2,957 | |
| KARBAC | 722 | 662 | 1,384 |  | GENICA | 527 | 532 | 1,059 |  | MCBCS | | 1,497 | | 1,890 | | | 3,387 | |
| KBCP | 445 | 251 | 696 |  | GESBC | 639 | 871 | 1,510 |  | MCCS | | 575 | | 467 | | | 1,042 | |
| kConFab/AOCS | 613 | 897 | 1,510 |  | HABCS | 1,108 | 1,001 | 2,109 |  | MEC | | 691 | | 721 | | | 1,412 | |
| LMBC | 2,671 | 1,388 | 4,059 |  | HEBCS | 723 | 7 | 730 |  | MTLGEBCS | | 466 | | 422 | | | 888 | |
| MARIE | 1,818 | 1,778 | 3,596 |  | HMBCS | 895 | 840 | 1,735 |  | NBCS | | 21 | | 67 | | | 88 | |
| MBCSG | 488 | 400 | 888 |  | HUBCS | 938 | 1,467 | 2,405 |  | NBHS_TN | | 122 | | 116 | | | 238 | |
| MCBCS | 1,862 | 1,931 | 3,793 |  | KARBAC | 35 | 98 | 133 |  | OBCS | | 470 | | 373 | | | 843 | |
| MCCS | 614 | 511 | 1,125 |  | KBCP | 50 | 265 | 315 |  | ORIGO | | 332 | | 323 | | | 655 | |
| MEC | 731 | 741 | 1,472 |  | LMBC | 166 | 96 | 262 |  | OSU | | 205 | | 203 | | | 408 | |
| MTLGEBCS | 489 | 436 | 925 |  | MARIE | 4 | 3 | 7 |  | PBCS | | 487 | | 397 | | | 884 | |
| NBCS | 22 | 70 | 92 |  | MBCSG | 255 | 945 | 1,200 |  | RPCI | | 46 | | 109 | | | 155 | |
| NBHS_TN | 125 | 118 | 243 |  | MCBCS | 243 | 460 | 703 |  | SASBAC | | 995 | | 1,222 | | 2,217 | | |
| OBCS | 507 | 414 | 921 |  | MCCS | 363 | 211 | 574 |  | SBCS | | 740 | | 826 | | 1,566 | | |
| OFBCR | 1,175 | 511 | 1,686 |  | MEC | 18 | 22 | 40 |  | SEARCH | | 8,752 | | 7,661 | | 16,413 | | |
| ORIGO | 357 | 327 | 684 |  | MSKCC | 495 | 455 | 950 |  | SKKDKFZS | | 110 | | 165 | | 275 | | |
| OSU | 207 | 203 | 410 |  | NBCS | 1,712 | 1,898 | 3,610 |  | SZBCS | | 291 | | 295 | | 586 | | |
| PBCS | 519 | 424 | 943 |  | NC-BCFR | 389 | 154 | 543 |  | UKBGS | | 406 | | 460 | | 866 | | |
| pKARMA | 5,434 | 5,537 | 10,971 |  | NHS | 1,216 | 1,690 | 2,906 |  | pKARMA | | 4,105 | | 4,489 | | 8,594 | | |
| RBCS | 664 | 699 | 1,363 |  | OBCS | 4 | 31 | 35 |  | **Total** | | **33,673** | | **33,381** | | **67,054** | | |
| RPCI | 136 | 126 | 262 |  | OFBCR | 75 | 7 | 82 |  |  | |  | |  | |  | | |
| SASBAC | 1,163 | 1,378 | 2,541 |  | ORIGO | 1,059 | 792 | 1,851 |  |  | |  | |  | |  | | |
| SBCS | 843 | 848 | 1,691 |  | PBCS | 1,688 | 1,926 | 3,614 |  |  | |  |  | | |  | | |
| SEARCH | 9,347 | 8,069 | 17,416 |  | POSH | 919 | 0 | 919 |  |  | |  |  | | |  | | |
| SKKDKFZS | 136 | 168 | 304 |  | RBCS | 56 | 3 | 59 |  |  | |  |  | | |  | | |
| SZBCS | 365 | 315 | 680 |  | SASBAC | 40 | 108 | 148 |  |  | |  |  | |  | | | |
| UKBGS | 476 | 470 | 946 |  | SBCS | 371 | 421 | 792 |  |  | |  |  | |  | | | |
| **Total** | **46,450** | **42,599** | **89,049** |  | SEARCH | 760 | 926 | 1,686 |  |  | |  |  | |  | | | |
|  |  |  |  |  | SKKDKFZS | 1,512 | 0 | 1,512 |  |  | |  |  | |  | | | |
|  |  |  |  |  | SZBCS | 906 | 1,001 | 1,907 |  |  |  | |  | |  | | | |
|  |  |  |  |  | UCIBCS | 938 | 543 | 1,481 |  |  |  | |  | |  | | | |
|  |  |  |  |  | UKBGS | 2,355 | 2,342 | 4,697 |  |  |  | |  | |  | | | |
|  |  |  |  |  | US3SS | 1,642 | 1,274 | 2,916 |  |  |  | |  | |  | | | |
|  |  |  |  |  | USRT | 857 | 1,053 | 1,910 |  |  |  | |  | |  | | | |
|  |  |  |  |  | kConFab/AOCS | 127 | 84 | 211 |  |  |  | |  | |  | | | |
|  |  |  |  |  | **Total** | **35,490** | **36,524** | **72,014** |  |  |  | |  | |  | | | |

†Additional studies from BCAC not included in iCOGS replication

## Supplementary Table 3: Summary of cases and controls included in SNP*SNP interaction analysis and PRS analysis

|  | **All breast cancers** | | | **ER-positive** | | | **ER-negative** | | |
| --- | --- | --- | --- | --- | --- | --- | --- | --- | --- |
|  | **No. Women** | **No. studies** | **Age (years)§** | **No. Women** | **No.**  **Studies** | **Age**  **(years)** | **No. women** | **No.**  **Studies** | **Age**  **(years)** |
| **SNP*SNP analyses**† | | | | | | | | | |
| **Cases** | **46,450** | **41** | **56(16)** | **27,074** | **34** | **58(15)** | **7,413** | **40** | **54(17)** |
| **Controls** | **42,599** | **41** | **55(16)** | **41,776** | **34** | **56(16)** | **42,202** | **40** | **55(16)** |
| **PRS analyses‡** | | | | | | | | | |
| **Cases** | **33,673** | **33** | **57(16)** | **21,365** | **26** | **58(16)** | **5,738** | **31** | **55(17)** |
| **Controls** | **33,381** | **33** | **56(15)** | **32,558** | **26** | **56(15)** | **32,984** | **31** | **56(15)** |

No, number; PRS, Polygenic Risk Score

†For SNP*SNP analyses, cases and controls were of European origin, and studies which oversampled for cases with a family history were not excluded. 2,441 (2·7%) of cases were ductal carcinomas in-situ and 849 (0·95%) were of unknown histopathology.

**‡**For PRS analyses, cases and controls were of European origin, and studies which oversampled for cases with a family history were excluded. However, 5,793 women from 24 studies not oversampling for family history reported positive family history in one or more first degree relative and were included in the analyses. 31,902 women from 23 studies reported no family history of breast cancer. Cases had all been diagnosed with invasive breast cancer.

**§**Age (years) is age at diagnosis for cases and age at interview for controls. Median (Inter-quartile ranges) are shown.

## Supplementary Table 4. SNPs included in SNP*SNP analysis† and PRS analysis and effect sizes for association with breast cancer or subtypes of the disease‡

| **SNP** | **Alleles** | **Locus** | **Chromosome** | **All breast cancers** | **ER-positive disease** | **ER-negative disease** | **SNP** | **Alleles** | **Locus** | **Chromosome** | **All breast cancers** | **ER-positive disease** | **ER-negative disease** |
| --- | --- | --- | --- | --- | --- | --- | --- | --- | --- | --- | --- | --- | --- |
| **rs78540526** | C/T | *CCND1* | 11 | 1.1761 | 1.2342 | 0.9049 | **rs4849887** | C/T | 2q14.2 | 2 | 0.9187 | 0.9128 | 0.9174 |
| **rs75915166** | C/A | *CCND1* | 11 | 1.0239 | 1.0189 | 1.1326 | **rs2016394** | G/A | 2q31.1 | 2 | 0.9504 | 0.9340 | 0.9966 |
| **rs554219** | C/G | *CCND1* | 11 | 1.1238 | 1.1402 | 1.0094 | **rs1550623** | A/G | *CDCA7* | 2 | 0.9445 | 0.9477 | 0.9417 |
| **rs7726159** | C/A | *TERT* | 5 | 1.0359 | 1.0442 | 1.0306 | **rs6762644** | A/G | 3p26.2 | 3 | 1.0661 | 1.0681 | 1.0256 |
| **rs10069690** | C/T | *TERT* | 5 | 1.0242 | 0.9948 | 1.1200 | **rs12493607** | G/C | *TGFBR2* | 3 | 1.0529 | 1.0642 | 1.0040 |
| **rs2736108** | C/T | *TERT* | 5 | 0.9379 | 0.9507 | 0.8964 | **rs9790517** | C/T | *TET2* | 4 | 1.0481 | 1.0524 | 1.0208 |
| **rs2588809** | C/T | *RAD51L1* | 14 | 1.0667 | 1.0829 | 1.0117 | **rs6828523** | C/A | *ADAM29* | 4 | 0.9056 | 0.8753 | 1.0205 |
| **rs999737** | C/T | *RAD51L1* | 14 | 0.9239 | 0.9189 | 0.9688 | **rs10472076** | T/C | *RAB3C* | 5 | 1.0419 | 1.0312 | 1.0452 |
| **rs10759243** | C/A | 9q31.2 | 9 | 1.0542 | 1.0732 | 1.0085 | **rs1353747** | T/G | *PDE4D* | 5 | 0.9213 | 0.9355 | 0.9158 |
| **rs865686** | T/G | 9q31 | 9 | 0.8985 | 0.8723 | 0.9891 | **rs1432679** | A/G | *EBF1* | 5 | 1.0670 | 1.0672 | 1.0699 |
| **rs2981579** | G/A | *FGFR2* | 10 | 1.2524 | 1.3175 | 1.0170 | **rs11242675** | T/C | *FOXQ1* | 6 | 0.9429 | 0.9459 | 0.9300 |
| **rs11199914** | C/T | 10q26.12 | 10 | 0.9400 | 0.9203 | 1.0166 | **rs204247** | A/G | *RANBP1* | 6 | 1.0503 | 1.0645 | 1.0044 |
| **rs7072776** | G/A | *DNAJC1* | 10 | 1.0581 | 1.0835 | 0.9362 | **rs17529111** | A/G | 6q14.1 | 6 | 1.0457 | 1.0452 | 1.0370 |
| **rs11814448** | A/C | *DNAJC1* | 10 | 1.2180 | 1.2020 | 1.2365 | **rs720475** | G/A | 7q35 | 7 | 0.9452 | 0.9312 | 0.9991 |
| **rs13387042** | A/G | 2q35 | 2 | 0.8794 | 0.8592 | 0.9581 | **rs9693444** | C/A | 8p21.1 | 8 | 1.0730 | 1.0706 | 1.0870 |
| **rs16857609** | C/T | 2q35 | 2 | 1.0721 | 1.0720 | 1.0831 | **rs6472903** | T/G | 8q21.11 | 8 | 0.9124 | 0.9123 | 0.9327 |
| **rs11552449** | C/T | 1p13.2 | 1 | 1.0810 | 1.0889 | 1.0534 | **rs2943559** | A/G | *HNF4G* | 8 | 1.1334 | 1.1384 | 1.1009 |
| **rs11249433** | A/G | 1p11.2 | 1 | 1.0993 | 1.1226 | 0.9996 | **rs11780156** | C/T | 8q24.21 | 8 | 1.0691 | 1.0842 | 1.0368 |
| **rs1045485** | G/C | *CASP8* | 2 | 0.9644 | 0.9685 | 0.9549 | **rs7904519** | A/G | *TCF7L2* | 10 | 1.0584 | 1.0505 | 1.0625 |
| **rs4973768** | C/T | *SLC4A7* | 3 | 1.0938 | 1.1022 | 1.0605 | **rs3903072** | G/T | 11q13.1 | 11 | 0.9442 | 0.9404 | 0.9780 |
| **rs10941679** | A/G | 5p12 | 5 | 1.1198 | 1.1559 | 1.0253 | **rs11820646** | C/T | 11q24.3 | 11 | 0.9563 | 0.9553 | 0.9552 |
| **rs889312** | A/C | *MAP3K1* | 5 | 1.1176 | 1.1473 | 1.0538 | **rs12422552** | G/C | 12p13.1 | 12 | 1.0327 | 1.0371 | 1.0222 |
| **rs12662670** | T/G | *ESR1* | 6 | 1.1392 | 1.1200 | 1.1816 | **rs17356907** | A/G | *NTN4* | 12 | 0.9078 | 0.9008 | 0.9295 |
| **rs2046210** | G/A | *ESR1* | 6 | 1.0471 | 1.0273 | 1.1209 | **rs11571833** | A/T | *BRCA2* | 13 | 1.2609 | 1.2490 | 1.4619 |
| **rs13281615** | A/G | 8q24 | 8 | 1.0950 | 1.1112 | 1.0185 | **rs2236007** | G/A | *PAX9* | 14 | 0.9203 | 0.9052 | 0.9524 |
| **rs1011970** | G/T | *CDKN2A/B* | 9 | 1.0502 | 1.0379 | 1.1159 | **rs941764** | A/G | *CCDC88C* | 14 | 1.0636 | 1.0736 | 1.0355 |
| **rs2380205** | C/T | *ANKRD16* | 10 | 0.9771 | 0.9604 | 1.0004 | **rs17817449** | T/G | *FTO* | 16 | 0.9300 | 0.9341 | 0.8845 |
| **rs10995190** | G/A | *ZNF365* | 10 | 0.8563 | 0.8495 | 0.8740 | **rs13329835** | A/G | *CDYL2* | 16 | 1.0758 | 1.0883 | 1.0139 |
| **rs704010** | C/T | *ZMIZ1* | 10 | 1.0699 | 1.0801 | 1.0424 | **rs527616** | G/C | 18q11.2 | 18 | 0.9573 | 0.9577 | 0.9787 |
| **rs3817198** | T/C | *LSP1* | 11 | 1.0744 | 1.0824 | 1.0559 | **rs1436904** | T/G | *CHST9* | 18 | 0.9466 | 0.9362 | 0.9925 |
| **rs10771399** | A/G | *PTHLH* | 12 | 0.8629 | 0.8814 | 0.8381 | **rs4808801** | A/G | *SSBP4* | 19 | 0.9349 | 0.9354 | 0.9227 |
| **rs1292011** | A/G | 12q24 | 12 | 0.9219 | 0.9082 | 0.9825 | **rs3760982** | G/A | 19q13.31 | 19 | 1.0553 | 1.0538 | 1.0503 |
| **rs3803662** | G/A | *TOX3* | 16 | 1.2257 | 1.2458 | 1.1520 | **rs132390** | T/C | 22q12.2 | 22 | 1.1091 | 1.1132 | 1.0515 |
| **rs6504950** | G/A | *COX11* | 17 | 0.9340 | 0.9304 | 0.9676 | **rs6001930** | T/C | *MKL1* | 22 | 1.1345 | 1.1313 | 1.1214 |
| **rs8170** | G/A | 19p13 | 19 | 1.0314 | 0.9955 | 1.1415 | **rs4245739** | A/C | *MDM4* | 1 | 1.0291 | 0.9961 | 1.1640 |
| **rs2363956** | G/T | 19p13 | 19 | 1.0264 | 0.9954 | 1.1256 | **rs6678914** | G/A | *LGR6* | 1 | 0.9890 | 1.0058 | 0.9146 |
| **rs2823093** | G/A | *NRIP1* | 21 | 0.9274 | 0.9152 | 0.9857 | **rs12710696** | G/A | 2p24.1 | 2 | 1.0387 | 1.0128 | 1.1054 |
| **rs17879961** | A/G | *CHEK2* I157T | 22 | 1.3632 | 1.5081 | 0.9215 | **rs11075995** | A/T | *FTO* | 16 | 1.0368 | 1.0204 | 1.1016 |
| **rs616488** | A/G | *PEX14* | 1 | 0.9417 | 0.9674 | 0.8910 |  |  |  |  |  |  |  |

† In addition to the SNPs shown in this Table two SNPs, rs614367 in *CCND1* on Chromosome 11 and rs2981582 in on Chromosome 10, were used only in SNP*SNP analysis but not included in the PRS

**‡**The effect sizes are provided as Odds Ratios (OR). The ORs refer to the second allele. Linked SNPs: In CCND1: rs78540526 rs75915166 r2 =0.64; rs75915166 rs554219 r2 =0.38, rs78540526 rs554219 r2 =0.60. In TERT, rs7726159 rs10069690 r2 =0.45; rs10069690 rs2736108 r2 =0.01; rs7726159 rs2736108, r2 =0.01. In ESR1, rs12662670 rs2046210, r2 =0.12. Joint effects have also been estimated for some other SNP pairs in the same regions, but in low LD.

## Supplementary Table 5. SNP*SNP interaction tests in iCOGS†

| **Case-control analyses with p < 0.01** | | | | | **Case-only analyses with p < 0.01 and control-only results p > 0.01** | | | | |
| --- | --- | --- | --- | --- | --- | --- | --- | --- | --- |
| **snp1** | **snp2** | **Case-control** | **Case-only** | **Control-only** | **snp1** | **snp2** | **Case-only** | **Control-only** | **Case-control** |
| rs2981582 | rs10941679 | 0.00017 | 0.045 | 0.001 | rs1436904 | rs527616 | 0.00001 | 0.037 | 0.12 |
| rs2736108 | rs7072776 | 0.0004 | 0.006 | 0.05 | rs4849887 | rs999737 | 0.0005 | 0.91 | 0.005 |
| rs704010 | rs10995190 | 0.0005 | 0.002 | 0.09 | rs6001930 | rs10941679 | 0.001 | 0.61 | 0.06 |
| rs3903072 | rs1353747 | 0.0008 | 0.10 | 0.004 | rs17529111 | rs6504950 | 0.001 | 0.96 | 0.03 |
| rs6678914 | rs1011970 | 0.001 | 0.01 | 0.06 | **rs11199914** | **rs6828523** | **0.001** | **0.42** | **0.01** |
| rs3903072 | rs10472076 | 0.001 | 0.16 | 0.002 | **rs704010** | **rs10995190** | **0.002** | **0.09** | **0.0005** |
| rs2981579 | rs10941679 | 0.001 | 0.07 | 0.006 | rs4973768 | rs12662670 | 0.002 | 0.56 | 0.13 |
| rs1436904 | rs9693444 | 0.002 | 0.004 | 0.11 | **rs941764** | **rs11571833** | **0.002** | **0.05** | **0.003** |
| rs616488 | rs704010 | 0.002 | 0.02 | 0.05 | rs132390 | rs865686 | 0.003 | 0.29 | 0.24 |
| rs11820646 | rs1292011 | 0.002 | 0.03 | 0.006 | rs11075995 | rs720475 | 0.004 | 0.44 | 0.16 |
| rs2588809 | rs17356907 | 0.002 | 0.005 | 0.24 | **rs1436904** | **rs9693444** | **0.004** | **0.13** | **0.002** |
| rs554219 | rs1432679 | 0.003 | 0.45 | 0.009 | rs11814448 | rs17879961 | 0.004 | 0.80 | 0.9 |
| rs941764 | rs11571833 | 0.003 | 0.002 | 0.05 | rs2736108 | rs1011970 | 0.004 | 0.77 | 0.11 |
| rs13329835 | rs2588809 | 0.003 | 0.13 | 0.010 | rs720475 | rs614367 | 0.005 | 0.33 | 040 |
| rs9693444 | rs8170 | 0.003 | 0.01 | 0.20 | **rs2016394** | **rs999737** | **0.005** | **0.20** | **0.003** |
| rs2016394 | rs999737 | 0.003 | 0.005 | 0.20 | **rs2588809** | **rs17356907** | **0.005** | **0.24** | **0.002** |
| rs1432679 | rs614367 | 0.004 | 0.42 | 0.009 | rs2588809 | rs1432679 | 0.005 | 0.83 | 0.02 |
| rs11242675 | rs4973768 | 0.004 | 0.07 | 0.03 | **rs11814448** | **rs7726159** | **0.006** | **0.26** | **0.01** |
| rs889312 | rs10069690 | 0.004 | 0.09 | 0.01 | rs2236007 | rs10771399 | 0.006 | 0.69 | 0.08 |
| rs204247 | rs2380205 | 0.004 | 0.01 | 0.25 | rs11075995 | rs1436904 | 0.006 | 0.92 | 0.05 |
| rs4808801 | rs2380205 | 0.004 | 0.04 | 0.05 | rs12493607 | rs7726159 | 0.006 | 0.98 | 0.09 |
| rs4849887 | rs999737 | 0.005 | 0.0005 | 0.91 | **rs204247** | **rs2380205** | **0.006** | **0.25** | **0.004** |
| rs1436904 | rs11820646 | 0.005 | 0.10 | 0.05 | **rs2736108** | **rs7072776** | **0.006** | **0.05** | **0.0004** |
| rs7904519 | rs6504950 | 0.005 | 0.10 | 0.04 | rs4973768 | rs2046210 | 0.007 | 0.60 | 0.04 |
| rs11780156 | rs6828523 | 0.005 | 0.01 | 0.13 | rs2981582 | rs6828523 | 0.007 | 0.56 | 0.02 |
| rs616488 | rs2380205 | 0.006 | 0.06 | 0.02 | rs11249433 | rs75915166 | 0.007 | 0.61 | 0.04 |
| rs999737 | rs12022378 | 0.006 | 0.09 | 0.06 | rs6762644 | rs4849887 | 0.007 | 0.38 | 0.19 |
| rs13281615 | rs75915166 | 0.006 | 0.23 | 0.004 | rs13329835 | rs2380205 | 0.007 | 0.54 | 0.18 |
| rs204247 | rs999737 | 0.007 | 0.01 | 0.31 | rs12710696 | rs2380205 | 0.007 | 0.83 | 0.04 |
| rs999737 | rs10995190 | 0.007 | 0.07 | 0.19 | rs1011970 | rs7726159 | 0.008 | 0.37 | 0.02 |
| rs6762644 | rs1011970 | 0.007 | 0.10 | 0.07 | rs2380205 | rs1292011 | 0.008 | 0.33 | 0.25 |
| rs12710696 | rs10472076 | 0.007 | 0.04 | 0.02 | rs12710696 | rs10941679 | 0.008 | 0.89 | 0.06 |
| rs720475 | rs6504950 | 0.007 | 0.09 | 0.07 | rs9790517 | rs10941679 | 0.008 | 0.98 | 0.04 |
| rs6678914 | rs12493607 | 0.008 | 0.04 | 0.09 | rs2588809 | rs1011970 | 0.008 | 0.41 | 0.02 |
| rs12710696 | rs4808801 | 0.008 | 0.01 | 0.07 | rs10759243 | rs9693444 | 0.008 | 0.39 | 0.04 |
| rs1353747 | rs616488 | 0.008 | 0.03 | 0.11 | rs10995190 | rs75915166 | 0.009 | 0.90 | 0.10 |
| rs2588809 | rs7726159 | 0.009 | 0.18 | 0.04 | rs11571833 | rs1011970 | 0.009 | 0.83 | 0.30 |
| rs12662670 | rs12022378 | 0.009 | 0.02 | 0.17 | **rs204247** | **rs999737** | **0.009** | **0.31** | **0.007** |
| rs11571833 | rs7726159 | 0.009 | 0.18 | 0.02 | rs12422552 | rs1045485 | 0.009 | 0.82 | 0.04 |
| rs11814448 | rs7726159 | 0.009 | 0.006 | 0.26 | rs527616 | rs12422552 | 0.009 | 0.80 | 0.08 |
| rs17879961 | rs10941679 | 0.009 | 0.04 | 0.06 | rs2046210 | rs10995190 | 0.009 | 0.80 | 0.06 |
| rs17356907 | rs7072776 | 0.01 | 0.39 | 0.02 | rs1550623 | rs704010 | 0.01 | 0.91 | 0.06 |
| rs1432679 | rs2046210 | 0.01 | 0.23 | 0.02 | rs2736108 | rs527616 | 0.01 | 0.11 | 0.73 |
| rs11199914 | rs6828523 | 0.01 | 0.001 | 0.42 | rs11249433 | rs12022378 | 0.01 | 0.43 | 0.04 |
| rs1436904 | rs3903072 | 0.01 | 0.22 | 0.03 | rs7072776 | rs17879961 | 0.01 | 0.72 | 0.11 |

Interaction tests were Wald tests (two-sided)

In case-only analyses, tests in which case-control p-value are p < 0.01 are shown in bold

## Supplementary Table 6. SNP*SNP interaction case-control analyses (p<0.01): using alternative methods

| **Method 1**† | | |  | **Method 2‡** | | |  | **Method 3§** | | | | |
| --- | --- | --- | --- | --- | --- | --- | --- | --- | --- | --- | --- | --- |
| **SNP1** | **SNP2** | **P||** |  | **SNP1** | **SNP2** | **P||** |  | **SNP1** | **SNP2** | **P||** | **pHeter** | **I2** |
| rs2981582 | rs10941679 | 0.0002 |  | rs2981582 | rs10941679 | 0.0002 |  | rs2981582 | rs10941679 | 0.0001 | 0.09 | 24.0% |
| rs2736108 | rs7072776 | 0.0004 |  | rs2736108 | rs7072776 | 0.0004 |  | rs704010 | rs10995190 | 0.0003 | 0.91 | 0.0% |
| rs704010 | rs10995190 | 0.0005 |  | rs704010 | rs10995190 | 0.0005 |  | rs3903072 | rs1353747 | 0.0008 | 0.91 | 0.0% |
| rs3903072 | rs1353747 | 0.0008 |  | rs3903072 | rs1353747 | 0.0008 |  | rs2736108 | rs7072776 | 0.001 | 0.30 | 9.2% |
| rs6678914 | rs1011970 | 0.001 |  | rs6678914 | rs1011970 | 0.001 |  | rs1436904 | rs9693444 | 0.001 | 0.33 | 7.7% |
| rs3903072 | rs10472076 | 0.001 |  | rs3903072 | rs10472076 | 0.001 |  | rs2981579 | rs10941679 | 0.001 | 0.39 | 4.5% |
| rs2981579 | rs10941679 | 0.001 |  | rs2981579 | rs10941679 | 0.001 |  | rs9693444 | rs8170 | 0.001 | 0.93 | 0.0% |
| rs1436904 | rs9693444 | 0.002 |  | rs1436904 | rs9693444 | 0.002 |  | rs3903072 | rs10472076 | 0.002 | 0.007 | 38.9% |
| rs616488 | rs704010 | 0.002 |  | rs616488 | rs704010 | 0.002 |  | rs6678914 | rs1011970 | 0.002 | 0.74 | 0.0% |
| rs11820646 | rs1292011 | 0.002 |  | rs2588809 | rs17356907 | 0.002 |  | rs2588809 | rs17356907 | 0.002 | 0.08 | 24.8% |
| rs2588809 | rs17356907 | 0.003 |  | rs11820646 | rs1292011 | 0.002 |  | rs1436904 | rs11820646 | 0.002 | 0.66 | 0.0% |
| rs554219 | rs1432679 | 0.003 |  | rs554219 | rs1432679 | 0.003 |  | rs11820646 | rs1292011 | 0.002 | 0.003 | 41.5% |
| rs941764 | rs11571833 | 0.003 |  | rs941764 | rs11571833 | 0.003 |  | rs889312 | rs10069690 | 0.002 | 0.28 | 10.8% |
| rs13329835 | rs2588809 | 0.003 |  | rs13329835 | rs2588809 | 0.003 |  | rs2016394 | rs999737 | 0.002 | 0.01 | 37.0% |
| rs9693444 | rs8170 | 0.003 |  | rs9693444 | rs8170 | 0.003 |  | rs616488 | rs704010 | 0.003 | 0.55 | 0.0% |
| rs2016394 | rs999737 | 0.003 |  | rs2016394 | rs999737 | 0.003 |  | rs941764 | rs11571833 | 0.003 | 0.71 | 0.0% |
| rs1432679 | rs614367 | 0.004 |  | rs1432679 | rs614367 | 0.004 |  | rs204247 | rs999737 | 0.003 | 0.46 | 0.4% |
| rs11242675 | rs4973768 | 0.004 |  | rs11242675 | rs4973768 | 0.004 |  | rs11242675 | rs4973768 | 0.003 | 0.13 | 19.9% |
| rs889312 | rs10069690 | 0.004 |  | rs889312 | rs10069690 | 0.004 |  | rs7904519 | rs6504950 | 0.003 | 0.10 | 22.9% |
| rs204247 | rs2380205 | 0.004 |  | rs4808801 | rs2380205 | 0.004 |  | rs11780156 | rs6828523 | 0.004 | 0.15 | 18.5% |
| rs4808801 | rs2380205 | 0.004 |  | rs204247 | rs2380205 | 0.004 |  | rs12710696 | rs4808801 | 0.004 | 0.49 | 0.0% |
| rs4849887 | rs999737 | 0.005 |  | rs4849887 | rs999737 | 0.005 |  | rs616488 | rs2380205 | 0.004 | 0.78 | 0.0% |
| rs1436904 | rs11820646 | 0.005 |  | rs1436904 | rs11820646 | 0.005 |  | rs1432679 | rs2046210 | 0.005 | 0.13 | 20.6% |
| rs7904519 | rs6504950 | 0.005 |  | rs7904519 | rs6504950 | 0.005 |  | rs999737 | rs12022378 | 0.006 | 0.13 | 20.5% |
| rs11780156 | rs6828523 | 0.005 |  | rs11780156 | rs6828523 | 0.006 |  | rs6678914 | rs12493607 | 0.006 | 0.54 | 0.0% |
| rs616488 | rs2380205 | 0.006 |  | rs616488 | rs2380205 | 0.006 |  | rs6762644 | rs1011970 | 0.006 | 0.34 | 7.4% |
| rs999737 | rs12022378 | 0.006 |  | rs999737 | rs12022378 | 0.006 |  | rs2823093 | rs2363956 | 0.006 | 0.44 | 1.7% |
| rs13281615 | rs75915166 | 0.006 |  | rs204247 | rs999737 | 0.007 |  | rs12662670 | rs12022378 | 0.006 | 0.49 | 0.0% |
| rs204247 | rs999737 | 0.007 |  | rs999737 | rs10995190 | 0.007 |  | rs720475 | rs6504950 | 0.006 | 0.20 | 15.3% |
| rs999737 | rs10995190 | 0.007 |  | rs6762644 | rs1011970 | 0.007 |  | rs12710696 | rs10472076 | 0.007 | 0.68 | 0.0% |
| rs6762644 | rs1011970 | 0.007 |  | rs720475 | rs6504950 | 0.007 |  | rs554219 | rs1432679 | 0.007 | 0.85 | 0.0% |
| rs12710696 | rs10472076 | 0.007 |  | rs12710696 | rs10472076 | 0.007 |  | rs13329835 | rs2588809 | 0.007 | 0.66 | 0.0% |
| rs720475 | rs6504950 | 0.007 |  | rs12710696 | rs4808801 | 0.008 |  | rs4808801 | rs2380205 | 0.007 | 0.02 | 32.7% |
| rs6678914 | rs12493607 | 0.008 |  | rs6678914 | rs12493607 | 0.008 |  | rs17356907 | rs6472903 | 0.008 | 0.71 | 0.0% |
| rs12710696 | rs4808801 | 0.008 |  | rs1353747 | rs616488 | 0.008 |  | rs2981582 | rs6828523 | 0.009 | 0.41 | 3.3% |
| rs1353747 | rs616488 | 0.008 |  | rs12662670 | rs12022378 | 0.008 |  | rs17879961 | rs10941679 | 0.009 | 0.96 | 0.0% |
| rs2588809 | rs7726159 | 0.009 |  | rs11571833 | rs7726159 | 0.008 |  | rs999737 | rs10995190 | 0.009 | 0.26 | 11.6% |
| rs12662670 | rs12022378 | 0.009 |  | rs2588809 | rs7726159 | 0.009 |  | rs204247 | rs2380205 | 0.009 | 0.90 | 0.0% |
| rs11571833 | rs7726159 | 0.009 |  | rs11814448 | rs7726159 | 0.009 |  | - | - | - | - | - |
| rs11814448 | rs7726159 | 0.009 |  | rs1432679 | rs2046210 | 0.01 |  | - | - | - | - | - |
| rs17879961 | rs10941679 | 0.009 |  | rs17356907 | rs7072776 | 0.01 |  | - | - | - | - | - |
| rs17356907 | rs7072776 | 0.01 |  | rs11199914 | rs6828523 | 0.01 |  | - | - | - | - | - |
| rs1432679 | rs2046210 | 0.01 |  | - | - | - |  | - | - | - | - | - |
| rs11199914 | rs6828523 | 0.01 |  | - | - | - |  | - | - | - | - | - |

† For method 1, both main effects were considered as categorical variables, and regression adjusted for study

**‡** For method 2, both main effects were considered as continuous variables, and regression adjusted for study

**§** For method 3, both main effects were considered as categorical variables and studies meta-analysed

**||** Interaction tests were Wald tests (two-sided)

pheter test for heterogeneity based on Cochran's Q statistic; *I*2, describes the percentage of total variation across studies that is due to heterogeneity rather than chance. I2 for study heterogeneity in the meta-analyses were generally low.

## Supplementary Table 7. SNP*SNP interaction tests in the iCOGS-BCAC meta-analysis†

| **Case-control analyses with p < 0.01** | | | | | **Case-only analyses with p < 0.01 and control-only results p > 0.01** | | | | |
| --- | --- | --- | --- | --- | --- | --- | --- | --- | --- |
| **Snp1** | **Snp2** | **Case-only** | **Control-only** | **Case-control** | **Snp1** | **Snp2** | **Case-only** | **Control-only** | **Case-control** |
| rs2981582 | rs10941679 | 0.03 | 0.001 | 0.0002 | rs1436904 | rs527616 | 0.00001 | 0.04 | 0.12 |
| rs2736108 | rs7072776 | 0.006 | 0.05 | 0.0004 | rs2380205 | rs1292011 | 0.0002 | 0.12 | 0.13 |
| rs3903072 | rs1353747 | 0.10 | 0.004 | 0.0008 | **rs4849887** | **rs999737** | **0.0005** | **0.91** | **0.005** |
| rs6678914 | rs1011970 | 0.01 | 0.06 | 0.001 | rs4973768 | rs12662670 | 0.0005 | 0.30 | 0.224 |
| rs3903072 | rs10472076 | 0.16 | 0.002 | 0.001 | rs6001930 | rs10941679 | 0.001 | 0.61 | 0.06 |
| rs2981579 | rs10941679 | 0.07 | 0.006 | 0.001 | rs4973768 | rs2046210 | 0.001 | 0.71 | 0.03 |
| rs1436904 | rs9693444 | 0.004 | 0.11 | 0.002 | rs17529111 | rs6504950 | 0.001 | 0.96 | 0.03 |
| rs616488 | rs704010 | 0.02 | 0.05 | 0.002 | **rs11199914** | **rs6828523** | **0.001** | **0.42** | **0.01** |
| rs11820646 | rs1292011 | 0.03 | 0.006 | 0.002 | rs941764 | rs11571833 | 0.002 | 0.05 | 0.003 |
| rs2588809 | rs17356907 | 0.005 | 0.24 | 0.002 | rs132390 | rs865686 | 0.003 | 0.29 | 0.24 |
| rs554219 | rs1432679 | 0.45 | 0.01 | 0.003 | rs11075995 | rs720475 | 0.004 | 0.44 | 0.16 |
| rs941764 | rs11571833 | 0.002 | 0.05 | 0.003 | **rs1436904** | **rs9693444** | **0.004** | **0.11** | **0.002** |
| rs13329835 | rs2588809 | 0.13 | 0.01 | 0.003 | rs11814448 | rs17879961 | 0.004 | 0.80 | 0.186 |
| rs9693444 | rs8170 | 0.01 | 0.20 | 0.003 | rs3803662 | rs13281615 | 0.004 | 0.78 | 0.03 |
| rs2016394 | rs999737 | 0.005 | 0.20 | 0.003 | rs2736108 | rs1011970 | 0.004 | 0.77 | 0.11 |
| rs1432679 | rs614367 | 0.42 | 0.01 | 0.004 | rs720475 | rs614367 | 0.005 | 0.33 | 0.40 |
| rs11242675 | rs4973768 | 0.07 | 0.03 | 0.004 | rs3817198 | rs12662670 | 0.005 | 0.83 | 0.33 |
| rs889312 | rs10069690 | 0.09 | 0.01 | 0.004 | **rs2016394** | **rs999737** | **0.005** | **0.20** | **0.003** |
| rs204247 | rs2380205 | 0.006 | 0.25 | 0.004 | **rs2588809** | **rs17356907** | **0.005** | **0.24** | **0.002** |
| rs4808801 | rs2380205 | 0.04 | 0.05 | 0.004 | rs2588809 | rs1432679 | 0.005 | 0.83 | 0.02 |
| rs4849887 | rs999737 | 0.0005 | 0.91 | 0.005 | **rs11814448** | **rs7726159** | **0.006** | **0.26** | **0.009** |
| rs1436904 | rs11820646 | 0.10 | 0.05 | 0.005 | rs2236007 | rs10771399 | 0.006 | 0.70 | 0.08 |
| rs7904519 | rs6504950 | 0.10 | 0.04 | 0.005 | rs11075995 | rs1436904 | 0.006 | 0.92 | 0.05 |
| rs11780156 | rs6828523 | 0.01 | 0.13 | 0.005 | rs12493607 | rs7726159 | 0.006 | 0.98 | 0.09 |
| rs616488 | rs2380205 | 0.06 | 0.02 | 0.006 | **rs204247** | **rs2380205** | **0.006** | **0.25** | **0.004** |
| rs999737 | rs12022378 | 0.09 | 0.06 | 0.006 | rs704010 | rs1045485 | 0.006 | 0.16 | 0.14 |
| rs204247 | rs999737 | 0.009 | 0.31 | 0.007 | **rs2736108** | **rs7072776** | **0.006** | **0.05** | **0.0004** |
| rs704010 | rs10995190 | 0.10 | 0.06 | 0.007 | rs2981582 | rs6828523 | 0.007 | 0.56 | 0.02 |
| rs6762644 | rs1011970 | 0.10 | 0.07 | 0.007 | rs6762644 | rs4849887 | 0.007 | 0.38 | 0.19 |
| rs3803662 | rs2046210 | 0.17 | 0.02 | 0.007 | rs13329835 | rs2380205 | 0.007 | 0.54 | 0.18 |
| rs999737 | rs10995190 | 0.07 | 0.32 | 0.007 | rs12710696 | rs2380205 | 0.007 | 0.83 | 0.04 |
| rs12710696 | rs10472076 | 0.04 | 0.02 | 0.007 | rs1011970 | rs7726159 | 0.008 | 0.37 | 0.02 |
| rs720475 | rs6504950 | 0.09 | 0.07 | 0.007 | rs12710696 | rs10941679 | 0.008 | 0.89 | 0.06 |
| rs3803662 | rs12662670 | 0.37 | 0.007 | 0.007 | rs9790517 | rs10941679 | 0.008 | 0.98 | 0.04 |
| rs6678914 | rs12493607 | 0.04 | 0.09 | 0.008 | rs2588809 | rs1011970 | 0.008 | 0.41 | 0.02 |
| rs12710696 | rs4808801 | 0.01 | 0.07 | 0.008 | rs10759243 | rs9693444 | 0.008 | 0.39 | 0.04 |
| rs1353747 | rs616488 | 0.03 | 0.11 | 0.008 | rs11571833 | rs1011970 | 0.009 | 0.83 | 020 |
| rs2588809 | rs7726159 | 0.18 | 0.04 | 0.009 | rs204247 | rs999737 | 0.009 | 0.31 | 0.007 |
| rs12662670 | rs12022378 | 0.02 | 0.17 | 0.009 | rs12422552 | rs1045485 | 0.009 | 0.2 | 0.04 |
| rs11571833 | rs7726159 | 0.15 | 0.02 | 0.009 | rs527616 | rs12422552 | 0.009 | 0.80 | 0.07 |
| rs11814448 | rs7726159 | 0.006 | 0.26 | 0.009 | rs1550623 | rs704010 | 0.01 | 0.10 | 0.06 |
| rs17356907 | rs7072776 | 0.39 | 0.02 | 0.01 | rs2736108 | rs527616 | 0.01 | 0.11 | 0.73 |
| rs1432679 | rs2046210 | 0.23 | 0.02 | 0.01 | rs11249433 | rs12022378 | 0.01 | 0.43 | 0.04 |
| rs11199914 | rs6828523 | 0.001 | 0.42 | 0.01 | rs7072776 | rs17879961 | 0.01 | 0.72 | 0.11 |

† Interaction tests were Wald tests (two-sided)

In case-only analyses, tests in which case-control p-value are p < 0.01 are shown in bold

**Supplementary Table 8.** **Association between the PRS and breast cancer risk: theoretical and observed Odds ratios and 95% Confidence Intervals**

|  | **All breast cancers** | | **ER-positive disease** | | **ER-negative disease** | | |
| --- | --- | --- | --- | --- | --- | --- | --- |
|  | **OR** | **95%CI** | **OR** | **95%CI** | **OR** | **95%CI** |
| **Theoretical** | | | | | | | |
| <1% | 0.31 | 0.29 - 0.32 | 0.27 | 0.26 - 0.28 | 0.36 | 0.34 - 0.39 |
| 1-5% | 0.43 | 0.41 - 0.44 | 0.39 | 0.37 - 0.40 | 0.48 | 0.46 - 0.50 |
| 5-10% | 0.52 | 0.51 - 0.54 | 0.49 | 0.47 - 0.50 | 0.57 | 0.55 - 0.59 |
| 10-20% | 0.63 | 0.62 - 0.64 | 0.59 | 0.58 - 0.61 | 0.67 | 0.65 - 0.69 |
| 20-40% | 0.79 | 0.78 - 0.80 | 0.77 | 0.76 - 0.78 | 0.82 | 0.80 - 0.83 |
| 40-60% | 1.00 | - | 1.00 | - | 1.00 | - |
| 60-80% | 1.27 | 1.26 - 1.28 | 1.30 | 1.29 - 1.32 | 1.23 | 1.21 - 1.24 |
| 80-90% | 1.59 | 1.57 - 1.62 | 1.68 | 1.65 - 1.71 | 1.50 | 1.45 -1.53 |
| 90-95% | 1.91 | 1.86 - 1.95 | 2.05 | 2.00 - 2.10 | 1.75 | 1.68 - 1.81 |
| 95-99% | 2.35 | 2.28 - 2.42 | 2.59 | 2.50 - 2.68 | 2.09 | 1.99 - 2.19 |
| >99% | 3.31 | 3.18 - 3.45 | 3.80 | 3.62 - 3.98 | 2.81 | 2.62 - 3.00 |
| **Observed** | | | | | | | |
| <1% | 0.31 | 0.24 - 0.39 | 0.34 | 0.26 - 0.44 | 0.42 | 0.27 - 0.66 |
| 1-5% | 0.42 | 0.37 - 0.46 | 0.39 | 0.34 - 0.45 | 0.39 | 0.31 - 0.50 |
| 5-10% | 0.49 | 0.45 - 0.54 | 0.43 | 0.38 - 0.49 | 0.50 | 0.41 - 0.61 |
| 10-20% | 0.61 | 0.57 - 0.66 | 0.57 | 0.52 - 0.62 | 0.64 | 0.56 - 0.73 |
| 20-40% | 0.79 | 0.75 - 0.83 | 0.79 | 0.74 - 0.84 | 0.83 | 0.75 - 0.92 |
| 40-60% | 1.00 | - | 1.00 | - | 1.00 | - |
| 60-80% | 1.27 | 1.21 - 1.33 | 1.31 | 1.24 - 1.39 | 1.15 | 1.04 - 1.26 |
| 80-90% | 1.44 | 1.36 - 1.52 | 1.59 | 1.49 - 1.70 | 1.36 | 1.22 - 1.51 |
| 90-95% | 1.85 | 1.72 - 1.99 | 2.02 | 1.87 - 2.19 | 1.62 | 1.42 - 1.84 |
| 95-99% | 2.34 | 2.17 - 2.52 | 2.62 | 2.41 - 2.85 | 2.00 | 1.75 - 2.28 |
| >99% | 3.36 | 2.95 - 3.83 | 3.73 | 3.24 - 4.30 | 2.80 | 2.26 - 3.46 |

OR, Odds Ratio; Confidence Intervals, CI; PRS, Polygenic risk score

## Supplementary Table 9. SNPs included in Polygenic Risk Score analysis of pKARMA study and effect sizes for association with breast cancer

| SNP |  | All Studies in iCOGS | |  | All studies excluding pKARMA | |  | pKARMA only | |
| --- | --- | --- | --- | --- | --- | --- | --- | --- | --- |
|  |  | OR | 95% CI |  | OR | 95% CI |  | OR | 95% CI |
| rs17817449 |  | 0.93 | 0.91 - 0.95 |  | 0.94 | 0.92 - 0.96 |  | 0.88 | 0.83 - 0.94 |
| rs17356907 |  | 0.91 | 0.89 - 0.93 |  | 0.91 | 0.89 - 0.93 |  | 0.89 | 0.83 - 0.94 |
| rs16857609 |  | 1.07 | 1.05 - 1.10 |  | 1.07 | 1.04 - 1.10 |  | 1.08 | 1.01 - 1.15 |
| rs13387042 |  | 0.88 | 0.86 - 0.90 |  | 0.89 | 0.87 - 0.91 |  | 0.84 | 0.79 - 0.88 |
| rs13329835 |  | 1.08 | 1.05 - 1.10 |  | 1.08 | 1.05 - 1.11 |  | 1.06 | 0.99 - 1.14 |
| rs13281615 |  | 1.10 | 1.07 - 1.12 |  | 1.09 | 1.07 - 1.12 |  | 1.09 | 1.03 - 1.16 |
| rs12662670 |  | 1.14 | 1.13 - 1.22 |  | 1.14 | 1.09 - 1.20 |  | 1.11 | 0.99 - 1.26 |
| rs11552449 |  | 1.08 | 1.05 - 1.11 |  | 1.09 | 1.06 - 1.12 |  | 1.04 | 0.96 - 1.12 |
| rs11814448 |  | 1.22 | 1.17 - 1.35 |  | 1.23 | 1.13 - 1.33 |  | 1.20 | 0.99 - 1.44 |
| rs11780156 |  | 1.07 | 1.04 - 1.10 |  | 1.07 | 1.04 - 1.10 |  | 1.07 | 0.98 - 1.16 |
| rs11249433 |  | 1.10 | 1.08 - 1.12 |  | 1.10 | 1.08 - 1.13 |  | 1.09 | 1.03 - 1.16 |
| rs10995190 |  | 0.86 | 0.83 - 0.88 |  | 0.85 | 0.83 - 0.88 |  | 0.87 | 0.81 - 0.95 |
| rs10941679 |  | 1.12 | 1.09 - 1.15 |  | 1.11 | 1.08 - 1.14 |  | 1.15 | 1.08 - 1.23 |
| rs10771399 |  | 0.86 | 0.83 - 0.89 |  | 0.86 | 0.83 - 0.89 |  | 0.88 | 0.81 - 0.96 |
| rs9693444 |  | 1.07 | 1.05 - 1.10 |  | 1.07 | 1.04 - 1.10 |  | 1.08 | 1.01 - 1.14 |
| rs7904519 |  | 1.06 | 1.04 - 1.08 |  | 1.06 | 1.04 - 1.09 |  | 1.04 | 0.98 - 1.10 |
| rs7072776 |  | 1.06 | 1.03 - 1.08 |  | 1.05 | 1.02 - 1.08 |  | 1.10 | 1.03 - 1.17 |
| rs6828523 |  | 0.91 | 0.88 - 0.94 |  | 0.90 | 0.87 - 0.93 |  | 0.93 | 0.85 - 1.02 |
| rs6762644 |  | 1.07 | 1.04 - 1.09 |  | 1.06 | 1.04 - 1.09 |  | 1.08 | 1.02 - 1.15 |
| rs6504950 |  | 0.93 | 0.91 - 0.96 |  | 0.93 | 0.91 - 0.96 |  | 0.95 | 0.89 - 1.01 |
| rs6472903 |  | 0.91 | 0.89 - 0.94 |  | 0.91 | 0.88 - 0.94 |  | 0.92 | 0.86 - 0.99 |
| rs6001930 |  | 1.13 | 1.10 - 1.17 |  | 1.14 | 1.10 - 1.18 |  | 1.12 | 1.03 - 1.23 |
| rs4973768 |  | 1.09 | 1.07 - 1.12 |  | 1.09 | 1.07 - 1.12 |  | 1.11 | 1.05 - 1.18 |
| rs4808801 |  | 0.93 | 0.91 - 0.96 |  | 0.93 | 0.91 - 0.95 |  | 0.95 | 0.90 - 1.01 |
| rs3903072 |  | 0.94 | 0.92 - 0.96 |  | 0.95 | 0.93 - 0.97 |  | 0.92 | 0.87 - 0.98 |
| rs3817198 |  | 1.07 | 1.05 - 1.10 |  | 1.07 | 1.04 - 1.10 |  | 1.11 | 1.04 - 1.18 |
| rs3803662 |  | 1.23 | 1.20 - 1.26 |  | 1.23 | 1.19 - 1.26 |  | 1.23 | 1.15 - 1.31 |
| rs2981579 |  | 1.25 | 1.22 - 1.28 |  | 1.25 | 1.22 - 1.28 |  | 1.23 | 1.16 - 1.31 |
| rs2943559 |  | 1.13 | 1.09 - 1.18 |  | 1.14 | 1.09 - 1.19 |  | 1.09 | 0.98 - 1.22 |
| rs2823093 |  | 0.93 | 0.91 - 0.95 |  | 0.94 | 0.92 - 0.97 |  | 0.85 | 0.80 - 0.91 |
| rs2236007 |  | 0.92 | 0.90 - 0.94 |  | 0.92 | 0.89 - 0.95 |  | 0.93 | 0.86 - 0.99 |
| rs2046210 |  | 1.05 | 1.02 - 1.07 |  | 1.05 | 1.03 - 1.08 |  | 1.01 | 0.94 - 1.07 |
| rs1432679 |  | 1.07 | 1.04 - 1.09 |  | 1.08 | 1.05 - 1.10 |  | 1.02 | 0.96 - 1.08 |
| rs1353747 |  | 0.92 | 0.89 - 0.96 |  | 0.91 | 0.88 - 0.95 |  | 1.00 | 0.90 - 1.11 |
| rs1292011 |  | 0.92 | 0.90 - 0.94 |  | 0.93 | 0.91 - 0.96 |  | 0.85 | 0.80 - 0.90 |
| rs999737 |  | 0.92 | 0.90 - 0.94 |  | 0.92 | 0.90 - 0.95 |  | 0.88 | 0.82 - 0.94 |
| rs889312 |  | 1.12 | 1.09 - 1.14 |  | 1.11 | 1.08 - 1.14 |  | 1.17 | 1.09 - 1.24 |
| rs865686 |  | 0.90 | 0.88 - 0.92 |  | 0.9 | 0.88 - 0.92 |  | 0.91 | 0.86 - 0.96 |
| rs704010 |  | 1.07 | 1.05 - 1.09 |  | 1.07 | 1.05 - 1.10 |  | 1.06 | 1.00 - 1.13 |
| rs616488 |  | 0.94 | 0.92 - 0.96 |  | 0.94 | 0.92 - 0.96 |  | 0.95 | 0.89 - 1.01 |
| rs554219 |  | 1.26 | 1.22 - 1.30 |  | 1.26 | 1.22 - 1.30 |  | 1.27 | 1.17 - 1.38 |
| rs2363956 |  | 1.03 | 1.00 - 1.05 |  | 1.02 | 1.00 - 1.05 |  | 1.05 | 0.99 - 1.11 |
| rs1011970 |  | 1.05 | 1.02 - 1.08 |  | 1.06 | 1.02 - 1.09 |  | 1.02 | 0.94 - 1.10 |

## Supplementary Table 10: Odds ratio for family history of breast cancer in any first degree relative stratified by percentiles of the PRS

|  | **Controls** | |  | **Cases** | |  |  |
| --- | --- | --- | --- | --- | --- | --- | --- |
| Percentiles of the PRS | **N** | **FH (%yes)** |  | **N** | **FH (%yes)** | **OR*** | **95%CI** |
| **<1%** | 149 | 8.1% |  | 65 | 16.9% | 3.18 | 1.20 - 8.44 |
| **1-5%** | 599 | 10.4% |  | 310 | 14.2% | 1.48 | 0.91 - 2.42 |
| **5-10%** | 748 | 8.6% |  | 478 | 15.3% | 1.77 | 1.17 - 2.69 |
| **10-20%** | 1496 | 8.6% |  | 1166 | 13.8% | 1.57 | 1.18 - 2.08 |
| **20-40%** | 2992 | 10.3% |  | 2984 | 15.9% | 1.57 | 1.32 - 1.87 |
| **40-60%** | 2992 | 10.2% |  | 3911 | 17.6% | 1.99 | 1.70 - 2.34 |
| **60-80%** | 2992 | 12.3% |  | 4915 | 19.0% | 1.61 | 1.39 - 1.85 |
| **80-90%** | 1496 | 12.6% |  | 2805 | 19.5% | 1.70 | 1.40 - 2.07 |
| **90-95%** | 748 | 13.8% |  | 1627 | 20.2% | 1.60 | 1.22 - 2.09 |
| **95-99%** | 599 | 15.2% |  | 1839 | 22.2% | 1.72 | 1.31 - 2.25 |
| **>99%** | 149 | 19.5% |  | 670 | 24.9% | 1.48 | 0.90 - 2.43 |

FH, family history; OR, Odds Ratio; CI, Confidence Intervals; PRS, polygenic risk score

N, number of women with information on family history in each quintile of the PRS.

*Odds Ratio for developing breast cancer for women with a family history of breast cancer in a first degree relative compared with women without a family history.

**Supplementary Table 11. Examples of power calculations for SNP*SNP analyses**†

| **SNP1** | | |  | **SNP2** | | |  | **Interaction OR** | | | |
| --- | --- | --- | --- | --- | --- | --- | --- | --- | --- | --- | --- |
| SNP | Allele frequency | per-allele OR |  | SNP | Allele frequency | per-allele OR |  | 1.05 | 1.1 | 1.15 | 1.2 |
| rs999737 | 0.22 | 0.92 |  | rs9790517 | 0.23 | 1.05 |  | 3% | 65% | 100% | 100% |
| rs6828523 | 0.11 | 0.91 |  | rs6762644 | 0.4 | 1.07 |  | 1% | 42% | 96% | 100% |
| rs6828523 | 0.11 | 0.91 |  | rs6001930 | 0.11 | 1.14 |  | 0% | 5% | 36% | 80% |
| rs7904519 | 0.46 | 1.06 |  | rs3760982 | 0.47 | 1.05 |  | 19% | 99% | 100% | 100% |
| rs4849887 | 0.09 | 0.92 |  | rs2981579 | 0.43 | 1.25 |  | 1% | 29% | 88% | 100% |
| rs11814448 | 0.02 | 1.23 |  | rs2981579 | 0.43 | 1.25 |  | 0% | 1% | 7% | 27% |
| rs10771399 | 0.11 | 0.86 |  | rs2981579 | 0.43 | 1.25 |  | 1% | 40% | 95% | 100% |
| rs3760982 | 0.47 | 1.05 |  | rs2981579 | 0.43 | 1.25 |  | 18% | 99% | 100% | 100% |
| rs13387042 | 0.48 | 0.88 |  | rs2981579 | 0.43 | 1.25 |  | 18% | 99% | 100% | 100% |

†Power was calculated using the computer program QUANTO (see Methods). Parameters inputed were allele frequency and OR for marginal effect of each SNP, sample size of 40,000 cases and 40,000 controls, significance level 0.00016, and breast cancer population prevalence of 0.08.

## References

(1) Michailidou K, Hall P, Gonzalez-Neira A et al. Large-scale genotyping identifies 41 new loci associated with breast cancer risk. Nat Genet 2013;45(4):353-2.

(2) Easton DF, Pooley KA, Dunning AM et al. Genome-wide association study identifies novel breast cancer susceptibility loci. Nature 2007;447(7148):1087-93.

(3) Turnbull C, Ahmed S, Morrison J et al. Genome-wide association study identifies five new breast cancer susceptibility loci. Nat Genet 2010;42(6):504-7.

(4) Antoniou AC, Easton DF. Polygenic inheritance of breast cancer: Implications for design of association studies. Genet Epidemiol 2003;25(3):190-202.

(5) Yang XR, Chang-Claude J, Goode EL et al. Associations of breast cancer risk factors with tumor subtypes: a pooled analysis from the Breast Cancer Association Consortium studies. J Natl Cancer Inst 2011;103(3):250-63.

(6) Bojesen SE, Pooley KA, Johnatty SE et al. Multiple independent variants at the TERT locus are associated with telomere length and risks of breast and ovarian cancer. Nat Genet 2013;45(4):371-2.

(7) French JD, Ghoussaini M, Edwards SL et al. Functional variants at the 11q13 risk locus for breast cancer regulate cyclin D1 expression through long-range enhancers. Am J Hum Genet 2013;92(4):489-503.

(8) Ahmed S, Thomas G, Ghoussaini M et al. Newly discovered breast cancer susceptibility loci on 3p24 and 17q23.2. Nat Genet 2009;41(5):585-90.

(9) Cox A, Dunning AM, Garcia-Closas M et al. A common coding variant in CASP8 is associated with breast cancer risk. Nat Genet 2007;39(3):352-8.

(10) Ghoussaini M, Song H, Koessler T et al. Multiple loci with different cancer specificities within the 8q24 gene desert. J Natl Cancer Inst 2008;100(13):962-6.

(11) Ghoussaini M, Fletcher O, Michailidou K et al. Genome-wide association analysis identifies three new breast cancer susceptibility loci. Nat Genet 2012;44(3): 312:318.

(12) Turnbull C, Ahmed S, Morrison J et al. Genome-wide association study identifies five new breast cancer susceptibility loci. Nat Genet 2010;42(6):504-7.

(13) Howie BN, Donnelly P, Marchini J. A Flexible and Accurate Genotype Imputation Method for the Next Generation of Genome-Wide Association Studies. PLoS Genet 2009;5(6):e1000529. doi: 10.1371/journal.pgen.1000529.

(14) Gauderman WJ, Morrison JM. QUANTO 1.1: A computer program for power and sample size calculations for genetic-epidemiology studies. 2006.

(15) Gauderman WJ. Sample size requirements for association studies of gene-gene interaction. Am J Epidemiol 2002;155(5):478-84.

(16) Pharoah PD, Antoniou A, Bobrow M, Zimmern RL, Easton DF, Ponder BA. Polygenic susceptibility to breast cancer and implications for prevention. Nat Genet 2002;31(1):33-6.

(17) Antoniou AC, Pharoah PP, Smith P, Easton DF. The BOADICEA model of genetic susceptibility to breast and ovarian cancer. Br J Cancer 2004;91(8):1580-90.

(18) Curado M, Edwards B, Shin HR et al. Cancer Incidence in Five Continents. IX. 2007. Lyon, France, Lyon: IARC Scientific Publications.

(19) Dite GS, Jenkins MA, Southey MC et al. Familial risks, early-onset breast cancer, and BRCA1 and BRCA2 germline mutations. J Natl Cancer Inst 2003;%19;95(6):448-57.

(20) Schmidt MK, Tollenaar RA, de Kemp SR et al. Breast cancer survival and tumor characteristics in premenopausal women carrying the CHEK2*1100delC germline mutation. J Clin Oncol 2007;25(1):64-9.

(21) Schrauder M, Frank S, Strissel PL et al. Single nucleotide polymorphism D1853N of the ATM gene may alter the risk for breast cancer. J Cancer Res Clin Oncol 2008;134(8):873-82.

(22) Fletcher O, Johnson N, Palles C et al. Inconsistent association between the STK15 F31I genetic polymorphism and breast cancer risk. J Natl Cancer Inst 2006;%19;98(14):1014-8.

(23) Colleran G, McInerney N, Rowan A et al. The TGFBR1*6A/9A polymorphism is not associated with differential risk of breast cancer. Breast Cancer Res Treat 2010;119(2):437-42.

(24) Yang R, Dick M, Marme F et al. Genetic variants within miR-126 and miR-335 are not associated with breast cancer risk. Breast Cancer Res Treat 2011;127(2):549-54.

(25) Villeneuve S, Fevotte J, Anger A et al. Breast cancer risk by occupation and industry: analysis of the CECILE study, a population-based case-control study in France. Am J Ind Med 2011;54(7):499-509.

(26) Weischer M, Bojesen SE, Tybjaerg-Hansen A, Axelsson CK, Nordestgaard BG. Increased risk of breast cancer associated with CHEK2*1100delC. J Clin Oncol 2007;25(1):57-63.

(27) Milne RL, Ribas G, Gonzalez-Neira A et al. ERCC4 associated with breast cancer risk: a two-stage case-control study using high-throughput genotyping. Cancer Res 2006;66(19):9420-7.

(28) Seal S, Thompson D, Renwick A et al. Truncating mutations in the Fanconi anemia J gene BRIP1 are low-penetrance breast cancer susceptibility alleles. Nat Genet 2006;38(11):1239-41.

(29) Fostira F, Tsitlaidou M, Papadimitriou C et al. Prevalence of BRCA1 mutations among 403 women with triple-negative breast cancer: implications for genetic screening selection criteria: a Hellenic Cooperative Oncology Group Study. Breast Cancer Res Treat 2012;134(1):353-62.

(30) Widschwendter M, Apostolidou S, Raum E et al. Epigenotyping in peripheral blood cell DNA and breast cancer risk: a proof of principle study. PLoS One 2008;3(7):e2656.

(31) Rahman N, Seal S, Thompson D et al. PALB2, which encodes a BRCA2-interacting protein, is a breast cancer susceptibility gene. Nat Genet 2007;39(2):165-7.

(32) Tchatchou S, Riedel A, Lyer S et al. Identification of a DMBT1 polymorphism associated with increased breast cancer risk and decreased promoter activity. Hum Mutat 2010;31(1):60-6.

(33) Frank B, Wiestler M, Kropp S et al. Association of a common AKAP9 variant with breast cancer risk: a collaborative analysis. J Natl Cancer Inst 2008;100(6):437-42.

(34) Frank B, Hemminki K, Wappenschmidt B et al. Association of the CASP10 V410I variant with reduced familial breast cancer risk and interaction with the CASP8 D302H variant. Carcinogenesis 2006;27(3):606-9.

(35) Justenhoven C, Pierl CB, Haas S et al. The CYP1B1_1358_GG genotype is associated with estrogen receptor-negative breast cancer. Breast Cancer Res Treat 2008;111(1):171-7.

(36) Pesch B, Ko Y, Brauch H et al. Factors modifying the association between hormone-replacement therapy and breast cancer risk. Eur J Epidemiol 2005;20(8):699-711.

(37) Chang-Claude J, Eby N, Kiechle M, Bastert G, Becher H. Breastfeeding and breast cancer risk by age 50 among women in Germany. Cancer Causes Control 2000;11(8):687-95.

(38) Dork T, Bendix R, Bremer M et al. Spectrum of ATM gene mutations in a hospital-based series of unselected breast cancer patients. Cancer Res 2001;61(20):7608-15.

(39) Leu M, Humphreys K, Surakka I et al. NordicDB: a Nordic pool and portal for genome-wide control data. Eur J Hum Genet 2010;18(12):1322-6.

(40) Li J, Humphreys K, Heikkinen T et al. A combined analysis of genome-wide association studies in breast cancer. Breast Cancer Res Treat 2011; 126(3): 717:27.

(41) Bogdanova NV, Antonenkova NN, Rogov YI, Karstens JH, Hillemanns P, Dork T. High frequency and allele-specific differences of BRCA1 founder mutations in breast cancer and ovarian cancer patients from Belarus. Clin Genet 2010;78(4):364-72.

(42) Bogdanova N, Cybulski C, Bermisheva M et al. A nonsense mutation (E1978X) in the ATM gene is associated with breast cancer. Breast Cancer Res Treat 2009;118(1):207-11.

(43) Lindblom A, Rotstein S, Larsson C, Nordenskjold M, Iselius L. Hereditary breast cancer in Sweden: a predominance of maternally inherited cases. Breast Cancer Res Treat 1992;24(2):159-65.

(44) Margolin S, Werelius B, Fornander T, Lindblom A. BRCA1 mutations in a population-based study of breast cancer in Stockholm County. Genet Test 2004;8(2):127-32.

(45) Hartikainen JM, Tuhkanen H, Kataja V et al. An autosome-wide scan for linkage disequilibrium-based association in sporadic breast cancer cases in eastern Finland: three candidate regions found. Cancer Epidemiol Biomarkers Prev 2005;14(1):75-80.

(46) Beesley J, Jordan SJ, Spurdle AB et al. Association between single-nucleotide polymorphisms in hormone metabolism and DNA repair genes and epithelial ovarian cancer: results from two Australian studies and an additional validation set. Cancer Epidemiol Biomarkers Prev 2007;16(12):2557-65.

(47) De ML, Van LE, De NK et al. Does estrogen receptor negative/progesterone receptor positive breast carcinoma exist? J Clin Oncol 2008;26(2):335-6.

(48) Flesch-Janys D, Slanger T, Mutschelknauss E et al. Risk of different histological types of postmenopausal breast cancer by type and regimen of menopausal hormone therapy. Int J Cancer 2008;123(4):933-41.

(49) Catucci I, Verderio P, Pizzamiglio S et al. SNPs in ultraconserved elements and familial breast cancer risk. Carcinogenesis 2009;30(3):544-5.

(50) Olson JE, Ma CX, Pelleymounter LL et al. A comprehensive examination of CYP19 variation and breast density. Cancer Epidemiol Biomarkers Prev 2007;16(3):623-5.

(51) Giles GG, English DR. The Melbourne Collaborative Cohort Study. IARC Sci Publ 2002;156:69-70.

(52) Zheng W, Long J, Gao YT et al. Genome-wide association study identifies a new breast cancer susceptibility locus at 6q25.1. Nat Genet 2009;41(3):324-8.

(53) Comen E, Balistreri L, Gonen M et al. Discriminatory accuracy and potential clinical utility of genomic profiling for breast cancer risk in BRCA-negative women. Breast Cancer Res Treat 2011;127(2):479-87.

(54) Nordgard SH, Johansen FE, Alnaes GI et al. Genome-wide analysis identifies 16q deletion associated with survival, molecular subtypes, mRNA expression, and germline haplotypes in breast cancer patients. Genes Chromosomes Cancer 2008;47(8):680-96.

(55) John EM, Hopper JL, Beck JC et al. The Breast Cancer Family Registry: an infrastructure for cooperative multinational, interdisciplinary and translational studies of the genetic epidemiology of breast cancer. Breast Cancer Res 2004;6(4):R375-R389.

(56) Hankinson SE, Willett WC, Manson JE et al. Plasma sex steroid hormone levels and risk of breast cancer in postmenopausal women. J Natl Cancer Inst 1998;90(17):1292-9.

(57) Erkko H, Xia B, Nikkila J et al. A recurrent mutation in PALB2 in Finnish cancer families. Nature 2007;446(7133):316-9.

(58) Huijts PE, Vreeswijk MP, Kroeze-Jansema KH et al. Clinical correlates of low-risk variants in FGFR2, TNRC9, MAP3K1, LSP1 and 8q24 in a Dutch cohort of incident breast cancer cases. Breast Cancer Res 2007;9(6):R78.

(59) de Bock GH, Schutte M, Krol-Warmerdam EM et al. Tumour characteristics and prognosis of breast cancer patients carrying the germline CHEK2*1100delC variant. J Med Genet 2004;41(10):731-5.

(60) Garcia-Closas M, Brinton LA, Lissowska J et al. Established breast cancer risk factors by clinically important tumour characteristics. Br J Cancer 2006;95(1):123-9.

(61) Eccles D, Gerty S, Simmonds P, Hammond V, Ennis S, Altman DG. Prospective study of Outcomes in Sporadic versus Hereditary breast cancer (POSH): study protocol. BMC Cancer 2007;7:160.

(62) Tapper W, Hammond V, Gerty S et al. The influence of genetic variation in 30 selected genes on the clinical characteristics of early onset breast cancer. Breast Cancer Res 2008;10(6):R108.

(63) Wedren S, Lovmar L, Humphreys K et al. Oestrogen receptor alpha gene haplotype and postmenopausal breast cancer risk: a case control study. Breast Cancer Res 2004;6(4):R437-R449.

(64) MacPherson G, Healey CS, Teare MD et al. Association of a common variant of the CASP8 gene with reduced risk of breast cancer. J Natl Cancer Inst 2004;96(24):1866-9.

(65) Lesueur F, Pharoah PD, Laing S et al. Allelic association of the human homologue of the mouse modifier Ptprj with breast cancer. Hum Mol Genet 2005;14(16):2349-56.

(66) Rashid MU, Jakubowska A, Justenhoven C et al. German populations with infrequent CHEK2*1100delC and minor associations with early-onset and familial breast cancer. Eur J Cancer 2005;41(18):2896-903.

(67) Jakubowska A, Jaworska K, Cybulski C et al. Do BRCA1 modifiers also affect the risk of breast cancer in non-carriers? Eur J Cancer 2009;45(5):837-42.

(68) Swerdlow AJ, Jones ME, Schoemaker MJ et al. The Breakthrough Generations Study: design of a long-term UK cohort study to investigate breast cancer aetiology. Br J Cancer 2011;105(7):911-7.

(69) Anton-Culver H, Cohen PF, Gildea ME, Ziogas A. Characteristics of BRCA1 mutations in a population-based case series of breast and ovarian cancer. Eur J Cancer 2000;36(10):1200-8.

(70) Ziogas A, Gildea M, Cohen P et al. Cancer risk estimates for family members of a population-based family registry for breast and ovarian cancer. Cancer Epidemiol Biomarkers Prev 2000;9(1):103-11.

(71) Garcia-Closas M, Egan KM, Newcomb PA et al. Polymorphisms in DNA double-strand break repair genes and risk of breast cancer: two population-based studies in USA and Poland, and meta-analyses. Hum Genet 2006;119(4):376-88.

(72) Bhatti P, Doody MM, Alexander BH et al. Breast cancer risk polymorphisms and interaction with ionizing radiation among U.S. radiologic technologists. Cancer Epidemiol Biomarkers Prev 2008;17(8):2007-11.

(73) Rajaraman P, Bhatti P, Doody MM et al. Nucleotide excision repair polymorphisms may modify ionizing radiation-related breast cancer risk in US radiologic technologists. Int J Cancer 2008;123(11):2713-6.

(74) Sigurdson AJ, Bhatti P, Chang SC et al. Polymorphisms in estrogen biosynthesis and metabolism-related genes, ionizing radiation exposure, and risk of breast cancer among US radiologic technologists. Breast Cancer Res Treat 2009;118(1):177-84.

(75) Doody MM, Freedman DM, Alexander BH et al. Breast cancer incidence in U.S. radiologic technologists. Cancer 2006;106(12):2707-15.
